# Mediterranean-style dietary interventions in adults with cancer: a systematic review of the methodological approaches, feasibility, and preliminary efficacy

**DOI:** 10.1038/s41430-024-01426-8

**Published:** 2024-03-08

**Authors:** Aoife McHugh, Ellie O’Connell, Bridie Gurd, Paige Rae, Elena S. George, Amber S. Kleckner, Brenton J. Baguley

**Affiliations:** 1 School of Exercise and Nutrition Sciences, Deakin University, Burwood, VIC Australia; 2 Institute for Physical Activity and Nutrition, Deakin University, Geelong, VIC Australia; 3 Department of Pain and Translational Symptom Science, University of Maryland School of Nursing, Baltimore, MD USA

**Keywords:** Nutrition, Cancer

## Abstract

**Background:**

Cancer and its treatments can lead to excess body fat, decreases in lean mass, cardiotoxicity, and other side effects. The Mediterranean diet (MED-diet) has the potential to improve clinical and supportive care outcomes. The aim of this systematic review was to evaluate the feasibility, safety, and efficacy of the MED-diet on health outcomes in adults with cancer.

**Methods:**

Three databases were searched from inception to February 2023. Eligible studies included randomised controlled trials testing a MED-diet intervention among adults with cancer. Within- and between-group differences for adherence, dietary intake and health outcomes were extracted.

**Results:**

Fifteen studies describing fourteen interventions were included, and there were considerable differences in study design and implementation of the MED-diet. Studies were predominately in women with a history of breast cancer. The MED-diet was safe with no adverse events reported, and feasible with high adherence and/or increases in MED-diet-compliant foods. The MED-diet when applied with an energy restriction below estimated requirements for weight loss demonstrated reductions in body weight (range: −3.9 kg to −0.7 kg). Interventions that showed significant reductions in body weight also improved quality of life. There is limited evidence to evaluating the MED-diet on cardiovascular and inflammatory markers, and heterogenous MED-diet prescriptions impede definitive conclusions on these health outcomes.

**Conclusion:**

The MED-diet was feasible and safe for adults with cancer. There were reported benefits for weight loss following a MED-diet when an energy restriction was applied, however further evaluation to determine the effects on cardiometabolic biomarkers and other outcomes are required.

## Introduction

Globally, 19.3 million people were diagnosed with cancer in 2020 and cancer is now the leading cause of morbidity and mortality worldwide [1]. After cancer treatment, many adults are faced with adverse treatment-related side effects that accelerate aging, increase risk of co-morbidities, and reduce quality of life [2]. These side effects include cardiomyopathy, persistent fatigue, reduced muscle mass with simultaneous increases in fat mass, cognitive impairment, and early menopause for women with breast cancer (which includes risks for bone health and cardiovascular disease) [2–4]. Several guidelines suggest nutrition and exercise interventions are important strategies to address persistent treatment-related toxicities and late side effects from cancer treatment [5, 6]. However, despite clear evidence for nutritional interventions in adults with cancer who are at risk of malnutrition [7], the optimal nutrition prescription to address other treatment-related toxicities (i.e., body composition, cardiovascular and metabolic health) after cancer treatment is yet to be determined.

The Mediterranean style diet (MED-diet) is a high-quality pattern of eating, with consistent observational evidence associating the MED-diet with reduced risk of chronic disease [8, 9]. There are many forms of the MED-diet, but it is typically characterised by (i) a high intake of fish, vegetables, fruits, legumes, nuts, and extra virgin olive oil; (ii) moderate intake of dairy products and red wine, and (iii) low consumption of added sugar, processed foods, and red meats [10]. Traditional versions of the MED-diet specify that dairy products are to be fermented, however, recent interpretations have been updated to include ‘low-fat’ dairy products [11]. The high antioxidant and anti-inflammatory properties of the MED-diet are proposed to offer synergistic benefits to cardiometabolic and body composition health [12, 13]. Observational evidence suggests the MED-diet can extend cancer survivorship, where high adherence to a MED-diet has shown a 22% and 13% reduction in prostate cancer and breast cancer mortality, respectively [14, 15]. The survivorship benefits from the MED-diet are potentially clinically important given the negative cardiometabolic and body composition changes from hormone therapy in breast and prostate cancer [16, 17]. Nutrition interventions in general, usually in combination with physical exercise, have shown promising results, particularly for patients with breast or prostate cancer by reducing body weight and fat mass [18] and improving quality of life [19]. However, despite several studies indicating the MED-diet may offer improvements to body composition and cardiometabolic health predominately after cancer treatment, the potential health benefits from the MED-diet in adults with cancer is yet to be systematically evaluated [13]. The aim of this review was to determine the feasibility (i.e., prescription, intervention design and support, participant adherence, safety) of delivering a MED-diet in adults with cancer, during or after treatment, and synthesise health outcomes from MED-diet intervention trials.

## Methods

### Protocol and registration

This systematic review was conducted in compliance with the Preferred Reporting Items for Systematic Reviews and Meta-Analyses (PRISMA) guidelines [20]. The protocol was registered in the PROSPERO database (registration ID: 376985).

### Information sources and search strategy

Embase, CINAHL, and Scopus databases were systematically searched on 17/02/2023. The search strategy was based on the Population Intervention Comparison and Outcomes (PICO) framework [21] and the specific search strategy was adjusted according to each database outlined in Supplementary Material 1. Each search concept included search terms relating to: (i) Mediterranean-style diet, (ii) adults with cancer, and (iii) randomised controlled trials. To summarise the evidence as a collective, outcome measures were not included in the search term strategy. Keyword search terms and Medical Subject Headings (MeSH) were applied to all search engines where applicable. Reference lists of identified articles were manually searched. There were no search restrictions on dates or language.

### Eligibility criteria

The inclusion criteria follows the PICO framework: (i) population: participants aged ≥18 years, of any sex, had a histological cancer diagnosis (any cancer type) prior to the intervention, and underwent any type of cancer treatment or were about to start treatment; (ii) intervention: any lifestyle intervention that included the MED-diet prescribed alone or in conjunction with exercise and/or psychosocial support; (iii) study design: randomised controlled trials where the control group received either no intervention or another intervention, a different dietary intervention, or a supplement, and (iv) reported the adherence, dietary intake, and/or any health-related outcome. Importantly, the nutrition intervention had to self-identify as the MED-diet or be based on the MED-diet principles outlined in the methods. Articles with similar dietary goals or nutritional targets to the MED-diet, yet did not explicitly mention the MED-diet, were excluded. Articles were also excluded if the intervention included the MED-diet plus a dietary supplement or medication, and/or both the intervention and control group received the MED-diet.

### Study selection

Four independent reviewers screened titles and abstracts for inclusion (AM, ES, BG, and PR). Studies that were not clearly excluded progressed to full text screening where the study was reviewed in detail against the inclusion criteria. Where discrepancies in inclusion occurred between reviewers, a fifth reviewer was sourced (BB) and issues were reconciled with discussion. Covidence systematic review software (Veritas Health Innovation, Melbourne, Australia, available at www.covidence.org) was used to organize the paper identification and complete data extraction.

### Data extraction

Data extracted included author(s), sample characteristics, trial length and design, data collection time points, control group, intervention methodology, and MED-diet prescription (macro and micronutrients and food groups). The primary outcome was extracted along with the intent of the MED-diet intervention on body weight. Descriptive data were extracted to report the feasibility of the MED-diet interventions (i.e., completion, consult attendance). Mean, standard deviation (SD) and/or 95% confidence interval for baseline values (where applicable), change within the intervention group, and between-group differences were extracted for changes in diet, health-related outcomes (weight and body composition), cardiovascular biomarkers, and quality of life.

### Quality assessment

The methodological quality was assessed using the Cochrane Risk of Bias Tool [21]. This tool looks at risk of bias in the following domains: sequence generation, allocation concealment, blinding, incomplete outcome data, selective outcome reporting, and other potential sources of systematic bias. Each study was rated against these domains and ranked as low, high, or unclear risk of bias. Blinding of participants to the intervention was assessed as “low risk” for all studies because it is not possible for the participant to be blinded to a dietary intervention supported by a health professional.

### Data synthesis

Continuous variables are presented as means and deviation from the mean (i.e., SD, Standard Error of Mean, range). Feasibility data are reported by the percentage of completion and attendance. Outcome variables (MED-diet adherence, body composition, biomarkers, quality of life) are reported as either an increase or decrease and whether statistically significant. Statistical significance was deemed where the *p*-value was <0.05.

## Results

**Fig. 1:**
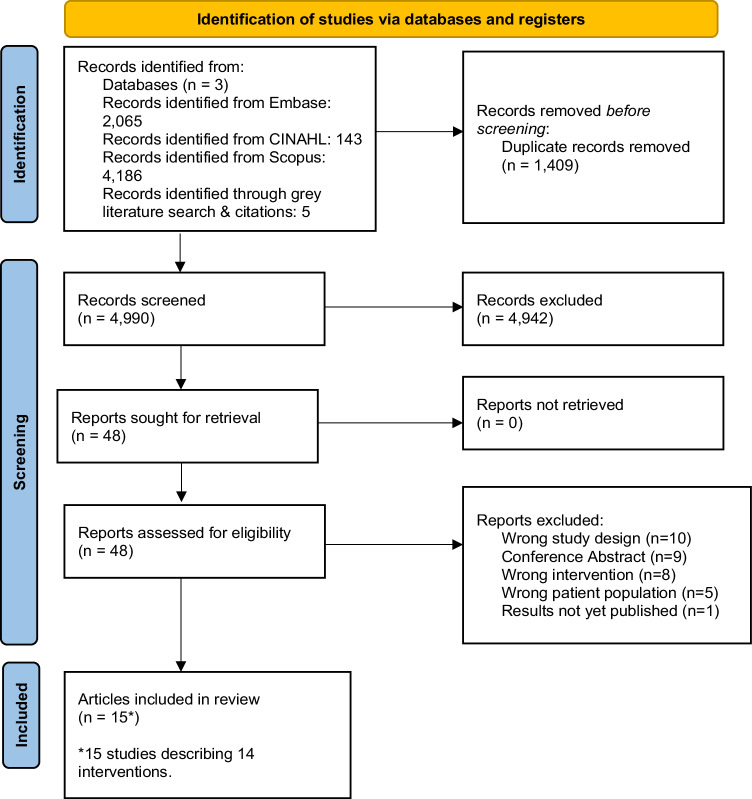
PRISMA flow diagram showing the study selection process. Preferred reporting items for systematic review and meta-analysis (PRISMA) statement flow diagram.

Fifteen articles [22–36] reporting fourteen interventions were included in this systematic review (Fig. 1). One intervention was reported in two articles [22, 23], and subsequently data from both articles were pooled to report the one intervention. In addition, Harvie et al. [28] evaluated two separate Mediterranean diet groups (delivered as home versus community group consultations) compared to a usual care group, and for the purpose of this systematic review these were treated as two separate intervention groups. The sample sizes ranged from 23 [22] to 1542 [25] participants; the average age of participants ranged from 41 [29] to 66 [22] years (Table 1).

**Table 1:** Study characteristics of Mediterranean-style dietary interventions in adults with cancer.

Reference (country)	Sample characteristics	Trial length & design, data collection time points	Control group	Intervention methodology	Mediterranean diet prescription	Primary outcome and intent of MED-diet on body weight
Baguley et al. 2020 & 2022 [22, 23] (Australia)	23 men, prostate cancer (12-int, 11-con) Treatment: Androgen Deprivation Therapy Intervention timing in relation to treatment: ≥ 3-months of Androgen Deprivation Therapy. Details of other treatment NR. BMI (Mean ± SD): 28.9 ± 3.4 Age (Mean ± SD): 65.9 ± 7.8	20-week RCT (pilot) Data collection: • Baseline • 8-weeks • 12-weeks • 20 weeks	Usual care, no dietary intervention	30–45 min consult with a dietitian during Androgen Deprivation Therapy every 2-weeks. Weeks 12–20 completed 3 HIIT sessions per week.	Energy: individual requirements via Harris Benedict equation CHO: 45–65% Protein: 15–25% Fat: 20–35% SFA: <10% total energy MUFA: NR PUFA: NR Refined carbs: limit consumption Fibre: 30 g/day Fruit: 2 serves/day Vegetables: 2 serve/day Lentils: 1 cup/day Lean meats: 3–4 times/week Meats with carcinogenic: reduce/eliminate Oily fish: 2–3 serves/week	Primary outcome: Feasibility, cancer-related fatigue, quality of life and body weight and composition. Intent: Weight loss. A dietary energy reduction of 2000–4000 kJ/day (480–950 kcal/d) was emphasized in the MED-diet prescription if BMI was classified as overweight (≥25 kg/m2)
Braakhuis et al. 2017 [24] (New Zealand)	50 women, breast cancer (stage I-III) (17- int, 16-LF int, 17-con) Treatment: Chemotherapy & surgery Intervention timing in relation to treatment: ≥ 3-years post chemotherapy, with/without present or past hormone therapy. BMI (Mean ± SD): 29.31 ± 5.62 Age (Mean ± SD): 54.71 ± 6.20	6-month RCT Data collection: • Baseline • 6-month	Usual care (no dietary intervention)	MED-diet plus olive oil life extract had monthly group education sessions with an unspecified instructor, for 6-months. Low fat diet group had the same monthly group educations, plus rice bran oil.	Energy: Ad libitum CHO: NR Protein: NR Fat: NR SFA: NR MUFA: NR PUFA: NR Recommended foods: wholegrains, fruit, fish, nuts, vegetables (specifically onion, leek, tomato and garlic) Discouraged foods: Red meat, butter, margarine, cream, carbonated drinks, sweets, chocolate and baked goods.	Primary outcome: Body weight Intent: Weight loss. Education on label reading, portion control, barriers to lifestyle changes.
Bruno et al. 2021 [25] (Italy)	1542 women, breast cancer (769-int, 773-con) Treatment: Hormonal/chemotherapy Intervention timing in relation to treatment: Not undergoing treatment. BMI (Mean ± SD): 26.7 ± 5.0 Age (Mean ± SD): 52.0 ± 8.5	5-year RCT Data collection: • Baseline • 1-year *(Years 2-5 currently in progress)*	Provided with World Cancer Research Fund/American Institute for Cancer Research 2007 recommendations for cancer prevention	MED-diet & macrobiotic principles delivered through monthly dietary activities (cooking classes, dietary reinforcement meetings), led by a nutritionist, based on MED-diet principles	Energy: NR CHO: NR Protein: NR Fat: NR SFA: NR MUFA: NR PUFA: NR Olive oil: Main source of fat Recommended foods: Wholegrains, legumes, high fibre vegetables, fruit, nuts, seeds & fish rich in omega-3 Discouraged foods: High glycaemic foods including refined starches, sugar & milk, red/processed meat, trans fats & animal protein Fermented macrobiotics: Soy sauce, miso & tempeh	Primary outcome: Body weight and metabolic syndrome. Intent: Weight loss. Calorie reduction through increase consumption of highly satiating, nutrient dense foods.
Cho et al. 2022 [26] (South Korea)	44 women, breast cancer (stage I–III) (23-int, 21-con) Treatment: Surgery/surgery + chemotherapy/radiotherapy, hormone therapy Intervention timing in relation to treatment: Post treatment. Mean BMI: 26.3 Mean age: 60.4	2-month RCT Data collection: • Baseline • 2-month	Dietary advice from the 2015 Dietary Reference Intakes for Koreans, energy intake target of 1,500 kcal & targeted macronutrient intakes	Dietary advice from a nutritionist plus naltrexone/ bupropion.	Energy: NR CHO: NR Protein: NR Fat: NR SFA: NR MUFA: NR PUFA: NR Recommended foods: NR	Primary outcome: Body weight and quality of life. Intent: Weight loss focused. Aimed for energy intake of 1500 kcal/day. Each meal limited to 500 kcal.
Gioxari et al. 2021 [27] (Greece)	30 (16-M, 14-F) SCLC & NSCLC (stage III-IV) (15-int, 15-con) Treatment: Chemotherapy, radiotherapy, immunotherapy, surgery >3-months Intervention timing in relation to treatment: During or post treatment BMI (Mean ± SD): 26.8 ± 5.5 Age (Mean ± SD): 52.2 ± 27.3	3-month RCT (pilot) Data collection: • Baseline • 3-month	15-day phone interviews to provide general nutritional guidelines based on American Cancer Society Guidelines on Nutrition & Physical Activity for Cancer Prevention	Personalised MED-diet intervention, individual counselling every 15-days with a dietitian.	Energy: Harris Benedict CHO: NR Fibre: 20–30 g/day Protein: 1.0–1.5 g/kg of body weight Fat: 30**%** SFA: < 10% MUFA: 10% PUFA: 10% Olive oil: olive oil over other plant-based oils Whole grains/unrefined cereals: consumed daily Herbal tea: 2–3 cups/day Fruit: NR Vegetables: Serves NR. Emphasised seasonal with high antioxidant capacity, consumed daily Fish, legumes & eggs: ≥ 1/week Meal preparation: trained on preparation techniques & promoted use of traditional Mediterranean herbs	Primary outcome: Inflammation and nutrition status. Intent: Weight maintenance.
Harvie et al. 2019 [28] (United Kingdom)	409 women breast cancer (134-home, 137-comm, 138-con), Treatment: Chemotherapy/hormone therapy/radiotherapy Intervention timing in relation to treatment: ≤12-weeks post-surgery with/without current adjuvant chemotherapy BMI (Mean ± SD): 26.9 ± 4.8-home, 27.0 ± 5.1-comm Age (Mean ± SD): 54.6 ± 11.2-home, 54.0 ± 9.2-comm	3-month RCT Data collection: • Baseline • 6-month • 12-month	General weight loss control advice & 150 min PA/week	**Home group:** MED-diet advice, consultation with a dietitian & home PA program. Fortnightly phone calls & goal summaries in weeks in between. 3-monthly trial newsletter. **Community group:** Same as Home group, plus 12- in person weekly PA & diet education.	Energy: NR CHO: 45% low-GI sources Protein: 25% lean meats Fat: 30% SFA: 7% MUFA: 15% PUFA: 8% Food groups: 5–7 serves of fruit & vegetables, nuts and seeds, whole grain cereals, olive oil, fish and seafood. Moderate dairy consumption. Discouraged food: Processed meat and lean meat <400 g/week.	Primary outcome: Body weight, hip and waist circumference, blood pressure, biochemical markers. Intent: Weight loss. Achieve ≥5% body weight loss by −25% EER.
Jalali et al. 2018 [29] (Iran)	50 (29-M, 21-F) Acute Myeloid Leukaemia (25-int, 25-con) Treatment: Chemotherapy Intervention timing in relation to treatment: Undergoing chemotherapy. Other treatment NR. BMI (Mean ± SD): 24 ± 3 Age (Mean ± SD): 41.2 ± 14.2	1-month RCT Data collection: • Baseline • 1-month	Neutropenic Diet. Calorie intake same as intervention group.	Supervision from nutritionists in hospital oncology department to follow MED-diet.	Energy: Mifflin formula CHO: 47% Protein: 15% Fat: 38% SFA: NR MUFA: 24% PUFA: NR Olive oil: 30 ml/day Recommended foods: Cooked foods and vegetables. Banana, orange, boiled water, pasturised and packed dairy products, well-cooked meat and egg.	Primary outcome: Nutrition status Intent: Weight maintenance to meet EER.
Kleckner et al. 2022 [30] (United States)	33 (2-M, 31-F), Breast cancer, other cancers (23-int, 10-con) Treatment: Chemotherapy Intervention timing in relation to treatment: Undergoing chemotherapy. Details of other treatment NR. BMI (Mean ± SD): 29.4 ± 6.8 Age (Mean ± SD): 51 ± 14.6	2-month RCT (pilot) Data collection: • Baseline • 1-month • 2-month	Usual care, no dietary intervention	Food provided for the first month via home delivery and 750 mL container to reheat frozen meals, information packet describing the MED-diet, cookbook specific to the program, 16 bags of walnuts, MED-diet pyramid, and exchange list. One session on behavioural change (goal setting, stimulus control, self-monitoring) delivered via phone or face-to-face in week three for 20-60 min with nutrition scientist.	Energy: NR CHO: NR Protein: NR Fat: NR SFA: NR MUFA: NR PUFA: NR Frozen, pre-packaged meals and fresh ingredients for the first 4 weeks and ad libitum MED-diet thereafter based on MED-Diet principles incorporated into daily diet.	Primary outcome: Cancer-related fatigue. Intent: NR
Long Parma et al. 2021 [31] (United States)	153 women, breast cancer (stage 0-III) (76-int, 77-con) Treatment: chemotherapy Intervention timing in relation to treatment: > 2-months post chemotherapy. Details of other treatment NR. BMI (Median): 31.9 Age (Mean ± SD): 55.28 ± 9.85	12-month RCT Data collection: • Baseline • 6-month • 12-month	Monthly American Institute for Cancer Research information brochures, & non-motivational phone calls prior to appointments.	Monthly, chef-led MED-diet culinary workshops for 6-months, motivational phone interviews with research staff, for 12-months. Also provided tailored newsletters and personal goal setting. Use of a nutritionist/dietitian to deliver the MED-diet not reported.	Energy: NR CHO: NR Protein: NR Fat: NR SFA: NR MUFA: NR PUFA: NR Recommended foods: Anti-inflammatory foods emphasised: Herbs & spices, marine fish, cruciferous vegetables, fruit, dark chocolate, green & black tea, & olive oil	Primary outcome: Quality of life. Intent: NR
Papandreou et al. 2021 [32] (Greece)	55 women breast cancer (stage I–IIA) (27- int, 28-con) Treatment: Surgery & hormone Intervention timing in relation to treatment: post-surgery and during hormone therapy BMI (Mean ± SD): 28.8 ± 5.6 Age (Mean ± SD): 49.7 ± 8.1	3-month RCT Data collection: • Baseline • 3-months	General lifestyle advice.	Individualised consultation on the MED-diet and lifestyle change with a dietitian (phone) at baseline and further facilitated using a clinical decision support system to adhere to the MED-diet.	Energy: Harris-Benedict CHO: NR Protein: 1.0–1.5 g/kg Fat: 30% SFA: < 10% MUFA: 10% PUFA: 10% Fibre: 20–30 g/day Food groups: NR	Primary outcome: Body weight Intent: Weight loss. Hypocaloric <500 kcal of TEE/day if BMI > 25 kg/m^2^
Ruiz-Vozmediano et al. 2020 [33] (Spain)	75 women breast cancer (stage IIA–IIB) (36- int, 36-con), Treatment: NR Intervention timing in relation to treatment: All treatment completed >12 months prior BMI (Mean ± SD): NR (eligibility BMI ≥ 30 kg/m2) Age (Mean ± SD): 48.33 ± 7.7	6-month RCT Data collection: • Baseline • 6-months	Usual care, no dietary intervention	3 × 5-hour MED-diet education workshops based on the European Code Against Cancer, benefits of MED-diet & dietary options for 2-weeks. Use of a nutritionist/dietitian to deliver the MED-diet not reported. Additional intervention components: Physical activity 3 × 60 min classes/week and mindfulness training.	Energy: NR CHO: NR Protein: NR Fat: NR SFA: NR MUFA: NR PUFA: NR Foods emphasised: Fruits, vegetables, nuts, grains, legumes, fish & dairy products. Foods to minimise: Red/processed meats, high fat products & salt, sugary & alcoholic beverages.	Primary outcome: Quality of life. Intent: weight loss.
Skouroliakou et al. 2017 [34] (Greece)	70 women, breast cancer (stage I–IIA) (35-int, 35-con) Treatment: NR Intervention timing in relation to treatment: during or post chemotherapy, radiotherapy and hormone therapy BMI (Mean ± SD): 29.2 ± 3.63 Age (Mean ± SD): NR	6-month RCT Data collection: • Baseline • 3-month • 6-months	Educated on American Cancer Society Guidelines on Nutrition & PA for Cancer Prevention	Individualised counselling with dietitians every 15-days to follow the MED-diet. Supporting material includes recipes, education booklets, cooking techniques.	Energy: NR CHO: NR Protein: NR Fat: NR SFA: NR MUFA: NR PUFA: NR Flaxseed: 1 TBSP of oil or 4 TBSP of ground flaxseed/day Green tea: 3 cups/day Fruit/vegetables: Seasonal with high antioxidant capacity	Primary outcome: Antioxidant biomarkers, body composition. Intent: Weight loss. Aim to decrease weight by 10% at 6 months when body fat percentage was above 33% (20-40 years of age) or 34% (40-60 years of age).
Villarini et al. 2012 [35] (Italy)	96 women, breast cancer (48-int, 48-con) Treatment: Surgery & chemotherapy Intervention timing in relation to treatment: Post surgery and undergoing current adjuvant chemotherapy BMI (Mean ± SD): 24.7 ± 4.5 Age (Mean ± SD): 52.7 ± 10.8	~3 month RCT Data collection: • Baseline • End of 1st chemo cycle • End of chemo (~3 months)	General recommendations for cancer prevention, baseline kitchen course	MED-diet & macrobiotic foods to prevent gastrointestinal symptoms during chemotherapy, delivered through cooking classes & communal meals twice per week throughout treatment. Use of a nutritionist/dietitian to deliver the MED-diet not reported.	Energy: NR CHO: NR Protein: NR Fat: NR SFA: NR MUFA: NR PUFA: NR Foods emphasised: Whole grains, vegetables, legumes, fruit, fish & olive oil, Macrobiotic: Miso, tamari, seaweeds & tofu Foods to minimise: Meat & cheese	Primary outcome: Change in body weight. Intent: Weight loss. Aimed to include highly satiating, nutrient dense foods that inadvertently decrease energy intake by approximately 250 kcal/day.
Zuniga et al. 2019 [36] (United States)	153 women, breast cancer (stage 0–III) (60-int, 65-con), Treatment: Surgery/chemotherapy/radiation/hormonal therapy/antibody therapy/reconstruction Intervention timing in relation to treatment: All treatment completed >2 months prior to intervention. BMI (Mean ± SD): 31.2 ± 4.1 Age (Mean ± SD): 55.3 ± 10.3	6-month RCT Data collection: • Baseline • 6-months	Monthly American Institute for Cancer Research brochures, 2 telephone calls prior to appointments, & no navigational services.	MED-diet guidelines & behaviour-change cues delivered during monthly nutrition workshops, motivational interviews via phone, patient navigators, & newsletters. Use of a nutritionist/dietitian to deliver the MED-diet not reported.	Energy: NR CHO: NR Protein: NR Fat: NR SFA: NR MUFA: NR PUFA: NR Recommended foods: NR	Primary outcome: Adherence to the MED-diet. Intent: NR

*AI* anti-inflammatory, *BMI* body mass index, *CHO* carbohydrate, *Comm* community group, *Con* control group, *EER* estimated energy requirement, *GI* glycaemic index, *HIIT* high intensity interval training, *Int* intervention group, *LF* low fat, *MED-diet* Mediterranean diet, *MUFA* mono-unsaturated fatty acids, *NR* not recorded, *NSCLC* non-small cell lung cancer, *PA* physical activity, *PUFA* poly-unsaturated fatty acids, *RCT* randomised control trial, *SCLC* small cell lung cancer, *SFA* saturated fatty acids, *TEE* total energy expenditure, *TEI* total energy intake.

Based on the Cochran risk-of-bias assessment, 12 of the 14 interventions scored positive [22, 24, 27–29, 33, 34, 36] (Supplementary Material 2). Jaleli [29] and Zuniga [36] had incomplete outcome data, which resulted in an unclear rating. Allocation concealment was the least positively (42%) scored outcome in the risk of bias tool.

### Study characteristics

Ten interventions included women with breast cancer [24–26, 28, 31–36] and one intervention included a mix of cancers however were mostly women with breast cancer [30]. Other interventions included people with each of the following cancer types: prostate cancer [22, 23], acute myeloid leukaemia [29], and lung cancer [27]. Five interventions included participants undergoing active treatment [22, 23, 29, 30, 32, 35] and five interventions commenced after cancer treatment [24, 26, 31, 33, 36]; that included two months [31, 36], three months [24], or twelve months [33] after treatment. Three interventions included participants either during or post-treatment [27, 28, 34], and one intervention commenced within 5 years post-diagnosis [25]. The intervention length ranged from one month [29] to twelve months [24, 25, 31, 33–36].

### Mediterranean diet characteristics

Details on the MED-diet intervention design was reported in all studies. Nine interventions were administered through a trained nutrition professional [22, 24–28, 30, 32, 34], six used individualised consultation to deliver the MED-diet [22, 27–29, 32, 34], five used education webinars/seminars [25, 31, 33, 35, 36], and two provided the MED-diet ingredients/meals to participants with consultation support [26, 30]. Variations in the MED-diet prescription were seen (Table 1), with nutrient or food group targets prescribed in seven of 14 interventions [22, 26–29, 32, 36]. Collectively, nutrient targets ranged from 20 to 40% of total energy from fat, 4 to 10% from polyunsaturated fat, 10 to 24% from monounsaturated fat, 45 to 65% from carbohydrates, and 15 to 25% from protein. Five interventions recommended food groups [22, 26, 28, 36]: 1.5 to 3 servings/day of fruit, 2 to 7 servings/day of vegetables, 2 to 4 servings/week of fish or seafood, 3 to 5 servings/week of nuts, and 15 to 30 ml/day of olive oil. Others indicated to increase or decreased food groups without specific targets. In addition, some interventions added olive leaf extract [24], emphasised fermented macrobiotics [25, 35], or flaxseed and green tea [34] to the MED-diet prescription.

Most interventions aimed to achieve weight loss [22, 24–26, 28, 32, 34, 35], whilst two aimed to prevent weight gain or maintain weight [27, 29], and others focused on reducing cancer-related fatigue [30] or pro-inflammatory cytokines [31], or improving diet quality [36], and did not report desired changes in body weight. Variations were seen in energy restriction applied to the MED-diet prescription across interventions. For participants classified as overweight or obese (BMI ≥ 25 kg/m^2^) energy restrictions ranged from −2000 to −4000 kJ/day [22], −250 kcal/day [35], −500 kcal/day [32], −25% estimated energy requirements [28], and total energy intake restricted to 1500 kcal/day [26]. Others aimed to achieve energy deficits through portion control [24], increasing satiating foods [25], or restricting energy by an unknown deficit [34]. MED-diet interventions that aimed to maintain body weight were prescribed to match estimated energy requirements to prevent malnutrition during treatment [27, 29].

### MED-diet adherence and dietary change

Eight of the 14 interventions reported adherence to the MED-diet [24, 26, 27, 30–33, 36], of which five used the Mediterranean Diet Adherence Score (MEDAS) tool [22, 23, 26, 30, 31, 33]. Of the eight interventions, six showed a significant improvement in adherence compared to the control group [24, 26, 27, 31, 34, 36]; whilst seven showed a significant increase in adherence within the MED-diet group only [23, 26, 27, 30–33]. No adverse events were attributed to the MED-diet in any of the 14 interventions.

Change in energy and nutrients from the MED-diet is represented in Table 2. Food Frequency Questionnaires and 3-day or 7-day food diaries were the predominant tool used to quantify energy and nutrient intake to compare to the MED-diet targets within each study. Eight interventions reported energy intake [22, 26, 28–31, 33, 36], only two reported a significant between-group reduction [31, 36]. Nine reported total fat and saturated fat (SFA) intake [22, 27, 29, 31–34], with two [26, 29] and three studies [22, 23, 27, 32], respectively, showing a between-group reduction following a MED-diet intervention. Five reported fibre intake [22, 27, 30–32] with two showing a between-group increase in fibre intake from the MED-diet intervention [22, 27].

**Table 2:** The effects of Mediterranean-style dietary interventions on health-related outcomes in adults with cancer.

Author (country)	Feasibility (completion, consult attendance, adverse events)	Dietary measure & adherence within the MED-diet intervention	Between-group effects on health outcomes		
			Anthropometrics (weight & body composition)	Biomarkers	Quality of Life
Baguley et al. 2020 & 2022 [22, 23] (Australia)	Completion: 82% Attendance: 100% Adverse events: Nil	MEDAS: ↑ Energy: ↓ Fibre: ↑ Protein: - Total fat: - SFA: ↓ MUFA: - PUFA: - LCN3FA: -	12wk: ↓ Body mass ↓ Fat mass ↓ Lean mass 20wk: ↓ Body mass - Fat mass - Lean mass	12wk: - IL-6 - IL-8 20wk: - IL-6 - IL-8	12wk: FACT ↑ General ↓ Fatigue 20wk: FACT - General - Fatigue ↑ Mental Health Composite
Braakhuis et al. 2017 [24] (New Zealand)	Completion: 80% Attendance: NR Adverse events: NR	MEDAS: ↑	6 m: - Weight ↓ BMI ↓ WC	6 m: - Total Cholesterol - HDL - LDL - TG - HbA1c	6 m: FACT-B - Total
Bruno et al. 2021 [25] (Italy)	Completion: 87% Attendance: NR Adverse events: NR	Dietary compliance index: ↑ Recommended foods: ↑ Discouraged foods: ↓	12 m: ↓ Body mass ↓ BMI ↓ WC	12 m: - HDL ↓ TG ↓ Fasting glucose ↓ BP	NR
Cho et al. 2022 [26] (South Korea)	Completion: 74% Attendance: NR Adverse events: NR	MEDAS: – Energy: ↓ MUFA/SFA ration: ↓ Fibre: ↓	8w: - Body mass - BMI - Fat mass - Lean mass	8w: - Total cholesterol - HDL - LDL ↓ TG - CRP - Fasting glucose - Insulin - WBC	NR
Gioxari et al. 2021 [27] (Greece)	Completion: 80% Attendance: NR Adverse events: NR	Adherence: ↑ Fibre: ↑ SFA: ↓ MUFA: ↑ PUFA: – Vitamin C: ↑	12wk: - Body mass - BMI - Fat mass	12wk: - Total cholesterol - HDL - LDL - TG - CRP ↓ ALI ↓ Glucose	NR
Harvie et al. 2019 [28] (United Kingdom)	Completion: 88%-home, 95%-community Attendance: 85%-home 64%-community Adverse events: NR	Home: Energy: – Fat: ↓ SFA: ↓ CHO: ↓ Community: Energy: ↓ Fat: ↓ SFA: ↓	6 m (home): ↓ Body mass ↓ Fat mass - Lean mass ↓ WC ↓ HC 12 m (home): ↓ Body mass ↓ Fat mass ↓ Lean mass ↓ WC ↓ HC 6 m (community): ↓ Body mass ↓ Fat mass - Lean mass ↓ WC ↓ HC 12 m (community): ↓ Body mass ↓ Fat mass ↓ Lean mass ↓ WC ↓ HC	6 m (home): - Total cholesterol - LDL - HDL - TG - Insulin - Glucose 12 m (home): - Total cholesterol - LDL - HDL - TG - Insulin - Glucose 6 m (community): ↓ Total cholesterol ↓ LDL - HDL - TG - Insulin - Glucose 12 m (community): ↓ Total cholesterol ↓ LDL - HDL ↓ TG ↓ Insulin - Glucose	6 m (home): FACT - Breast - Fatigue 12 m (home): - Breast - Fatigue 6 m (community): ↓ Breast - Fatigue 12 m (community): ↓ Breast - Fatigue
Jalali et al. 2018 [29] (Iran)	Completion: NR Attendance: NR Adverse events: NR	Energy: ↑ Carbohydrate: ↑ Protein: ↑ Total fat: ↑	4w: - Body mass - BMI	4w: - Albumin	NR
Kleckner et al. 2022 [30] (United States)	Completion: 100% Attendance: N/A Adverse events: Nil	MEDAS: ↑ Energy: – Fibre: – Protein: – Total fat: – CHO: – PUFA: – Magnesium: ↑ Wholegrains: ↑	8w: - Body mass	8w: - LDL - HDL - TG	8w: FACIT-F: ↑ Total ↑ Physical ↑ Fatigue BFI: ↑ Total: ↓ Usual fatigue ↓ Worse fatigue SI: ↑ Symptoms + QoL
Long Parma et al. 2021 [31] (United States)	Completion: 80% Attendance: NR Adverse events: NR	MEDAS: ↑ Energy: ↓ % CHO: – Fibre: – % Protein: – % Total fat: – % SFA: – Herbs & spices: ↑ Sodium: –	NR	NR	6 m: - FACT-G - BCS - Perceived stress scale 12 m: - FACT-G - BCS ↑ Perceived stress scale
Papandreou et al. 2021 [32] (Greece)	Completion: 80% Attendance: NR Adverse events: NR	MEDAS: ↑ Fibre: ↑ SFA: ↓ MUFA: ↑ Vitamin C: ↑ Vitamin D: –	3 m: ↓ Body weight ↓ BMI ↓ WC ↓ Fat mass	3 m: - Total Cholesterol ↑ HDL - LDL - TG - Glucose	3 m: EORTC-QLQ-C30, ↑ Role: ↑ Emotional ↑ Global HADS - Depression - Anxiety
Ruiz-Vozmediano et al. 2020 [33] (Spain)	Completion: 87% Attendance: 90% Adverse events: NR	MEDAS: ↑ Energy: – Protein: – Total fat: –	6 m: ↓ Body weight ↓ BMI	6 m: - Total cholesterol ↑ HDL - LDL ↓ TG - Glucose	6 m: EORTC-QLQ-C30 ↑ Physical: ↑ Role: ↑ Cognitive: - Global health
Skouroliakou et al. 2017 [34] (Greece)	Completion: 64% Attendance: NR Adverse events: NR	MEDAS: ↑ SFA: – MUFA: – PUFA: ↓ Vitamin A: ↑ Vitamin C: ↑ a-Tocopherol: ↓	3 m: ↓ Body weight ↓ BMI ↓ WC ↓ Fat mass	3 m: - Total cholesterol ↑ HDL - LDL - TG ↓ Glucose	NR
Villarini et al. 2012 [35] (Italy)	Completion: 98% Attendance: NR Adverse events: NR	Fruit/Vegetables: – Whole grains: ↑ Legumes: ↑ Sugar: ↓ Dairy: ↓ Processed meat: ↓ Refined cereals: ↓ Dairy: ↓	TP1: ↓ Body mass ↓ BMI ↓ Fat mass ↓ Fat free mass ↓ WC ↓ HC TP2: - Body mass - BMI - Fat mass - Fat free mass - WC - HC	NR	NR
Zuniga et al. 2019 [36] (United States)	Completion: 81% Attendance: NR Adverse events: NR	MEDAS: ↑ Energy: ↓ % CHO: – Fibre: – % Protein: – % Total fat: – % SFA: – Sodium: – Fruit/vegetables: –	NR NR	NR	NR

↑ denotes significant increase between MED-diet and control groups at endpoint.

↓ denotes significant decrease between MED-diet and control groups at endpoint.

– denotes non-significant changes both within group and between groups.

Data for Long Parma et al. represented in this table is at the 6-month timepoint. Data for Harvie et al. is comparing between-group differences in community vs. control and home vs. control.

*ALI* advanced lung cancer inflammation index, *BCS* breast cancer scale, *BFI* brief fatigue inventory, *BFM* body fat mass, *BMI* body mass index, *BMR* basal metabolic rate, *BP* blood pressure, *CHO* carbohydrate, *CoQ10* Coenzyme Q10, *CRP* C-reactive protein, *EORTC QLQ-C30 & BR23* The European Organisation for Research and Treatment – Quality of Life – Cancer & Breast cancer specific, *FACIT* functional assessment of chronic illness therapy, *FACT TOI-BC* functional assessment of cancer therapy – trial outcome indicator for breast cancer, *FACT-G* functional assessment of cancer therapy – general, *FFM* fat free mass, *FM* fat mass, *FFQ* food frequency questionnaire, *HADS* Hospital anxiety and depression scale, *HC* hip circumference, *HDL* high density lipo-protein, *IL-6 & IL-8* interlukin-6 & −8, *LCN3FA* long chain n-3 fatty acids, *LDL* low density lipo-protein, *LMM* lean muscle mass, *MDA* plasma malondialdehyde, *MEDAS* Mediterranean diet adherence score, *MUFA* mono-unsaturated fatty acid, *NR* not reported, *PUFA* poly-unsaturated fatty acids, *QoL* quality of life, *RBC* red blood cells, *SFA* saturated fatty acids, *SI* symptom inventory, *TG* triglyceride, *TP1* end of first chemotherapy cycle, *TP2* end of chemotherapy, *WBC* white blood cell, *WC* waist circumference.

### Anthropometric data

Thirteen interventions reported body weight at baseline and endpoint (Table 2). Six of the thirteen interventions reported a significant between-group decrease in body weight in favour of the MED-diet [22, 25, 28, 32, 33, 35]. Body mass index (BMI) was recorded in 11 interventions [22, 24, 26, 27, 29, 32–34, 36], seven reported a significant between-group decrease in BMI [24, 25, 32–35]. Additionally, body composition was (fat mass or lean mass) was reported in seven interventions [22, 26–28, 32, 34, 35]; five reported a significant between-group decrease [22, 28, 32, 34, 35], and three reported a significant between-group decrease in lean mass [22, 28, 35].

### Biomarkers

Twelve of the 14 interventions reported the effects of the MED-diet on biomarkers [22, 24–30, 32–35] including glucose metabolism, cardiovascular risk factors, protein stores, and inflammatory markers (Table 2). Three interventions reported a significant decrease in blood glucose levels [25–27, 34], four showed a significant decrease in triglycerides [25, 26, 28, 33], three increased HDL [32–34], and Harvie et al’s community-group showed a decrease in LDL and total cholesterol [28]. Individual trials reported a significant decrease interleukin-8 [22], and a significant increase in albumin [29].

### Quality of life

Quality of life measurements were assessed using either the Functional Assessment of Chronic Illness Therapy (FACT) [37] or the European Organisation for Research and Treatment of Cancer – Quality of Life Questionnaire (EORTC-QLQ-C30) [38]. Seven interventions reported the effects of the MED-diet on quality of life [22, 24, 28, 30–33]. Four interventions reported significant between-group improvements in quality of life in favour of the MED-diet [22, 28, 30, 32], whilst two showed significant improvements in fatigue [22, 30]. Other domains of quality of life that include role [32, 33], physical [30, 33], emotional [32], and cognitive health composite [23] were inconsistently improved across interventions (Table 2).

## Discussion

This review showed that the MED-diet is safe (zero adverse events), feasible, and well adhered to, however heterogeneity in the prescription of the MED-diet and intervention design precludes identifying the optimal approach to supporting individual health outcomes for adult cancer survivors. Hypocaloric MED-diets show particularly promising results for reducing body weight (range: −3.9 kg to −0.7 kg) in overweight or obese adults who have finished cancer treatment or currently treated with hormone therapy for breast or prostate cancer. Interventions with the goal to maintain weight showed promising findings in preventing weight loss during chemotherapy, however, both studies were small in sample size and individualised to manage nutritional impact symptoms (i.e., nausea and vomiting). Whilst evidence for weight loss from the MED-diet is in accordance with several guidelines advocating for healthy body weight post-treatment to reduce the risk of mortality and morbidity [5, 6], the current evidence for the MED-diet in patients with cancer is limited to mostly women who have finished breast cancer treatment. Despite the evidence to support the MED-diet for preventing and managing chronic diseases, translation of this evidence to cancer survivors, where chronic disease risk is high, requires further investigation.

### Feasibility of Mediterranean diet interventions

The MED-diet interventions in this review were safe (zero adverse events), feasible with a high completion rate (range: 64% to 98%), and showed high attendance to consultations or educational workshops (>75%). These factors likely contributed to the high adherence to the MED-diet food groups across interventions in this review. Most studies provided dietary goals for food groups that participants could follow and all studies incorporated guidance or accountability check-ins with trained staff, though the frequency of informative workshops or interaction with a trained nutrition professional. More intense intervention delivery may account for the improvement in MED-diet adherence scores amongst these studies [22, 23, 27, 28, 36], as behaviour change is more likely when dietary advice is paired with improved nutritional literacy [39]. Interventions delivered by a nutrition professional, with accompanied education material (i.e., recipes and cooking demonstrations), offers the chance to build rapport and iteratively change dietary behaviours across consultations, and is likely a contributing factor to the high adherence seen in these studies.

### Body weight and composition

This review indicates that the MED-diet when prescribed with an energy reduction has weight loss benefits (range: −3.9 kg to −0.7 kg). Similar reductions in weight were reported in a meta-analysis that evaluated the effects of MED-diet interventions compared to low fat diets (−4.1 to 10.1 kg vs. 2.9 to −5.0 kg) in overweight or obese adults [40]. Whilst MED-diet interventions have shown long term weight loss (≥12 months) in overweight or obese adults at risk of chronic disease [40, 41], the long term effect in cancer survivors are limited. Two 12-month MED-diet interventions prescribed by a nutrition specialist has demonstrated significant weight loss in women with breast cancer that are treated with hormone therapy (−2.4 kg vs. −0.9 kg) [25] or that are post-surgery and treated with adjuvant hormone and/or chemotherapy (home-based: −1.5 kg vs. 0.8 kg and community-based: −1.6 kg vs. 0.8 kg) [28]. The present review also suggests the MED-diet can prevent weight gain from hormone therapy in prostate cancer [22], however this study was underpowered in sample size. Whilst most studies in the present review were designed to promote weight loss, the methodological differences in intervention prescription, behaviour change support techniques, and reporting of dietary adherence varied. As such it is plausible that improvements in diet quality from the MED-diet, characterised by an increased consumption of vegetables, fruits, and whole grains led to weight loss.

Interventions that measured body composition showed reductions in fat mass but also lean mass. Whilst reductions in weight and fat mass is beneficial for optimising body composition, the reductions in lean mass seen in some studies in concerning for maintaining strength, physical function, and quality of life following or during treatment. Nonetheless, reductions in lean mass from weight loss interventions are expected [42], and our findings are in line with a previous meta-analysis evaluating nutrition and exercise interventions on body composition in adults with cancer reporting muscle loss (mean difference −0.58 kg) from weight-loss focused interventions [18]. Efforts to attenuate muscle loss in cancer survivorship is a research priority given most cancer treatments are associated with reduced lean mass. Increased protein intake (1.0–1.5 g/kg/day), and/or supplemental approaches (i.e., β-hydroxy β-methylbutyrate at 3 g/day) may protect muscle or increase muscle when paired with exercise [43]. Yet whether these approaches protect muscle mass within a MED-diet that intends to reduce body weight and fat mass requires investigation.

### Cardiometabolic health

The MED-diet promotes improvements in cardiometabolic health by encouraging foods high in monounsaturated and polyunsaturated fats and fibre, which play an important role in lowering low-density lipoprotein (LDL) and triglycerides and increasing HDL cholesterols [8, 9, 44]. A limited number of studies showed improvements in cardiovascular biomarkers, however results were inconsistent and varying intervention components preclude identifying whether the benefits in cardiovascular health were primarily attributable to the MED-diet. For example, studies varied in the MED-diet prescription, with some promoting a specified amount of olive leaf extract [24], flaxseed, and green tea consumption per day [34], or including macrobiotic foods like soy and miso [25], whilst others included an exercise program with the MED-diet [28, 33]. Secondly, most interventions ranged between 12 and 26 weeks, which may not be a sufficient duration needed for the MED-diet to see changes to cardiometabolic biomarkers. Whilst a larger body of evidence supports the use of the MED-diet in reducing cardiovascular biomarkers (LDL, triglycerides and cholesterol) in adults who already have chronic disease(s) [39, 45, 46], the benefits of the MED-diet in adults with cancer who are at a higher risk of chronic diseases however may not have clinically elevated biomarkers at baseline may be less sensitive to change in a short timeframe and therefore requires further investigation.

Chronic inflammation and oxidative stress are known predictive risk factors of cardiovascular disease [47]. The MED-diet effects on cardiometabolic health are theorised to be associated with dietary properties high in bioactive nutrients and phenolic compounds, which combat oxidation and lower circulating inflammatory markers [48–50]. Several biomarkers are used to identify endothelial cell damage and systemic inflammation, for example, interleukins (IL-6, IL-8, IL-1β), however, the types of inflammatory markers measured from the MED-diet in adults with cancer in this review varied and were often secondary or tertiary outcome measures. Inflammatory pathways and networks are complex and can be influenced by cancer treatment. As such, studies that are powered to investigate the effects of the MED-diet on inflammatory markers, considering covariates (i.e., medications and treatment), are needed before definitive conclusions can be made.

### Quality of life

This review provides preliminary evidence for quality-of-life benefits from the MED-diet in adults with cancer and corroborates previous reports of mixed benefits to quality of life from lifestyle interventions in cancer [51–53]. The studies that demonstrate high adherence to the MED-diet and improved global and domains of quality of life (i.e., fatigue, physical and cognitive functioning) also had improvements to body weight and composition (free fat mass, weight, BMI) [22, 23, 28, 33]. Our results suggest that weight loss from the MED-diet might have additional benefits in improving mental domains of quality of life and body image, which is a research priority highlighted elsewhere [54]. Most MED-diet interventions that were delivered by a dietitian showed improvements in quality of life or domains of quality of life (i.e., role, emotional). These findings may be attributable to the benefits of interaction with a health care professional, which offers individualisation of dietary preferences, social and environmental influences on dietary intake. The utilisation of behaviour change strategies within a consultation, where participants are empowerment to be actively involved in their cancer health, may contribute to the improvements seen in quality of life [55]. Whilst the MED-diet is likely to have improved quality of life, weight loss and interactions with a health professional may be mediators, and further research is needed to optimize the components of a MED-diet intervention for quality-of-life outcomes.

### Future directions and clinical implications

The results of this systematic review should be considered in light of its strengths and limitations. This review followed the PRISMA guidelines in reporting a systematic review and provides a high level of evidence for the benefits of the MED-diet compared to usual care across health outcomes. Whilst this review demonstrates that hypocaloric MED-diets may reduce weight for people with cancer that are overweight or obese, the effects on body composition (i.e., fat mass and muscle mass) are limited. The heterogeneity in MED-diet prescription, the intent of each intervention on body weight, and the use of co-interventions (i.e., exercise) precluded pooling the mean difference in a meta-analysis. This highlights the importance for future interventions to report MED-diet prescriptions using calculating estimated energy requirements, nutrient targets along with food groups to identify components of the MED-diet to health outcomes. Translation of the MED-diet in patients with cancer are limited to reducing or maintaining body weight. However, whether the benefits in weight control are attributable to the energy restriction or the MED-diet itself is unknown. Future longer term RCTs should focus on reducing the risk of, or managing, cardiovascular or metabolic disease after cancer treatment to improve the potential clinical implications of the MED-diet.

## Conclusion

This systematic review showed that the MED-diet is safe, feasible, and well adhered to among adult cancer survivors during and after treatment. Collectively, there was considerable variation in the MED-diet prescription (i.e., macronutrients, micronutrients, and food groups), health outcomes measured, resulting in mixed findings from the heterogeneity in intervention design. Interventions that prescribed an energy restriction in addition to the MED-diet showed reductions in body weight, however, whether the benefits in weight loss can be attributable to the MED-diet over and above the energy restriction requires further investigation. Whilst the MED-diet is consistent with dietary recommendations for cancer survivors, there is limited evidence to indicate that the MED-diet offers benefits to managing side effects, chronic disease prevention, or improving quality of life. Attention to reporting the MED-diet prescription and adherence on other important outcomes, such as cardiometabolic function, is important for evidence-based recommendations across multiple health outcomes for cancer survivors.

### Supplementary information

Supplementary Material 1

Supplementary Material 2
