# Supplementary material for: Mediterranean-style dietary interventions in adults with cancer: a systematic review of the methodological approaches, feasibility, and preliminary efficacy
Source: Eur J Clin Nutr. 2024 Mar 8;78(6):463–76. doi: 10.1038/s41430-024-01426-8 (PMC11182747; doi:10.1038/s41430-024-01426-8)
Supplement: Supplementary file 1 — Supplementary Material 1 [file 41430_2024_1426_MOESM1_ESM.docx]

**McHugh et al., Mediterranean-style dietary interventions in adults with cancer: A systematic review of the methodological approaches, feasibility, and preliminary efficacy**

**Suppl. Material 1:** Search Strategy

**Scopus**

( ( TITLE-ABS-KEY ( med* AND diet* ) ) OR ( TITLE-ABS ( med* AND diet* ) ) OR ( TITLE-ABS ( diet*, AND mediterranean ) ) ) AND ( ( TITLE-ABS ( cancer AND surviv* ) ) OR ( TITLE-ABS ( cancer* AND patient* ) ) OR ( TITLE-ABS ( cancer* AND surviv* ) ) OR ( TITLE-ABS ( cancer* ) ) OR ( TITLE-ABS ( neoplas* ) ) OR ( TITLE-ABS ( cancer AND prostate OR breast OR colorectal ) ) OR ( TITLE-ABS ( cancer* AND prostate OR breast OR colorectal ) ) OR ( TITLE-ABS ( tumo?r* ) ) OR ( TITLE-ABS ( malign* ) ) OR ( TITLE-ABS ( carcinoma ) ) ) AND ( ( TITLE-ABS ( intervention* ) ) OR ( TITLE-ABS ( trial* ) ) OR ( TITLE-ABS ( randomi?* AND control* AND trial* ) ) OR ( TITLE-ABS ( rct ) ) ) AND ( LIMIT-TO ( SRCTYPE , "j" ) ) AND ( LIMIT-TO ( DOCTYPE , "ar" ) ) AND ( LIMIT-TO ( LANGUAGE , "english" ) ) AND ( LIMIT-TO ( PUBYEAR , 2022 ) )

**Embase**

1. ‘med* diet*;ab,ti
2. ‘diet*, mediterranean’ab;ti
3. diet*;ab,ti
4. mediterranean;ab,ti
5. #1 OR #2 OR #3 OR #4
6. ‘cancer* surviv*;ab,ti
7. ‘cancer* patient*;ab,ti
8. cancer*;ab,ti
9. neoplas*;ab,ti
10. cancer* AND breast OR prostate OR colorectal;ab,ti
11. tumo?r*;ab,ti
12. malign*;ab,ti
13. carcinoma;ab,ti
14. #6 OR #7 OR #8 OR #9 OR #10 OR #11 OR #12 OR #13
15. intervention*;ab,ti
16. trial*;ab,ti
17. randomi?* AND control* AND trial*;ab,ti
18. rct;ab,ti
19. #15 OR #16 OR #17 OR #18
20. #5 AND #14 AND #19 AND ([embase]/lim OR [pubmed-not-medline]/lim)
21. #20 AND ('case control study'/de OR 'clinical trial'/de OR 'cohort analysis'/de OR 'comparative study'/de OR 'controlled clinical trial'/de OR 'controlled study'/de OR 'cross sectional study'/de OR 'crossover procedure'/de OR 'double blind procedure'/de OR 'human'/de OR 'human experiment'/de OR 'in vivo study'/de OR 'intervention study'/de OR 'major clinical study'/de OR 'multicenter study'/de OR 'normal human'/de OR 'observational study'/de OR 'prospective study'/de OR 'randomized controlled trial'/de OR 'randomized controlled trial topic'/de OR 'retrospective study'/de OR 'single blind procedure'/de) AND ('advanced cancer'/dm OR 'breast cancer'/dm OR 'cachexia'/dm OR 'colon cancer'/dm OR 'colorectal cancer'/dm OR 'digestive system cancer'/dm OR 'endometrium cancer'/dm OR 'esophagus cancer'/dm OR 'head and neck cancer'/dm OR 'inflammation'/dm OR 'liver cancer'/dm OR 'liver cell carcinoma'/dm OR 'lung cancer'/dm OR 'malignant neoplasm'/dm OR 'metastasis'/dm OR 'nausea'/dm OR 'neoplasm'/dm OR 'ovary cancer'/dm OR 'pancreas cancer'/dm OR 'prostate cancer'/dm OR 'stomach cancer'/dm)
22. #20 AND ('case control study'/de OR 'clinical trial'/de OR 'cohort analysis'/de OR 'comparative study'/de OR 'controlled clinical trial'/de OR 'controlled study'/de OR 'cross sectional study'/de OR 'crossover procedure'/de OR 'double blind procedure'/de OR 'human'/de OR 'human experiment'/de OR 'in vivo study'/de OR 'intervention study'/de OR 'major clinical study'/de OR 'multicenter study'/de OR 'normal human'/de OR 'observational study'/de OR 'prospective study'/de OR 'randomized controlled trial'/de OR 'randomized controlled trial topic'/de OR 'retrospective study'/de OR 'single blind procedure'/de) AND ('advanced cancer'/dm OR 'breast cancer'/dm OR 'cachexia'/dm OR 'colon cancer'/dm OR 'colorectal cancer'/dm OR 'digestive system cancer'/dm OR 'endometrium cancer'/dm OR 'esophagus cancer'/dm OR 'head and neck cancer'/dm OR 'inflammation'/dm OR 'liver cancer'/dm OR 'liver cell carcinoma'/dm OR 'lung cancer'/dm OR 'malignant neoplasm'/dm OR 'metastasis'/dm OR 'nausea'/dm OR 'neoplasm'/dm OR 'ovary cancer'/dm OR 'pancreas cancer'/dm OR 'prostate cancer'/dm OR 'stomach cancer'/dm) AND ([adult]/lim OR [young adult]/lim OR [middle aged]/lim OR [aged]/lim OR [very elderly]/lim)
23. #22 AND 2022:py

**CINAHL (EBSCOhost)**

1. TI "med* diet*" OR AB "med* diet*"
2. TI "diet*, mediterranean" OR AB "diet*, mediterranean"
3. AB diet* AND AB mediterranean
4. TI diet* AND TI mediterranean
5. S1 OR S2 OR S3 OR S4
6. TI cancer, surviv* OR AB cancer, surviv*
7. TI "cancer* patient*" OR AB "cancer* patient*"
8. TI cancer, patient* OR AB cancer, patient*
9. TI cancer* OR AB cancer*
10. TI neoplas* OR AB neoplas*
11. AB cancer* AND AB breast OR AB prostate OR AB colorectal
12. TI cancer* AND TI breast OR TI prostate OR TI colorectal
13. TI tumo?r* OR AB tumo?r*
14. TI malign* OR AB malign*
15. TI carcinoma OR AB carcinoma
16. S6 OR S7 OR S8 OR S9 OR S10 OR S11 OR S12 OR S13 OR S14 OR S15
17. TI intervention* OR AB intervention*
18. TI trial* OR AB trial*
19. TI "randomi?* control* trial*" OR AB "randomi?* control* trial*"
20. TI RCT OR AB RCT
21. S17 OR S18 OR S19 OR S20
22. S5 AND S16 AND S21
