# Supplementary material for: Mediterranean-style dietary interventions in adults with cancer: a systematic review of the methodological approaches, feasibility, and preliminary efficacy
Source: Eur J Clin Nutr. 2024 Mar 8;78(6):463–76. doi: 10.1038/s41430-024-01426-8 (PMC11182747; doi:10.1038/s41430-024-01426-8)
Supplement: Supplementary file 2 — Supplementary Material 2 [file 41430_2024_1426_MOESM2_ESM.docx]

| Study | Random sequence generation (selection bias) | Allocation concealment  (selection bias) | Blinding of participants and personnel (performance bias) | Blinding of outcome assessment (detection bias) | Incomplete outcome data  (attrition bias) | Selective reporting  (reporting bias) | Other sources of bias |
| --- | --- | --- | --- | --- | --- | --- | --- |
| Baguley et al. (2020 & 2022) ^(22,23)^ | 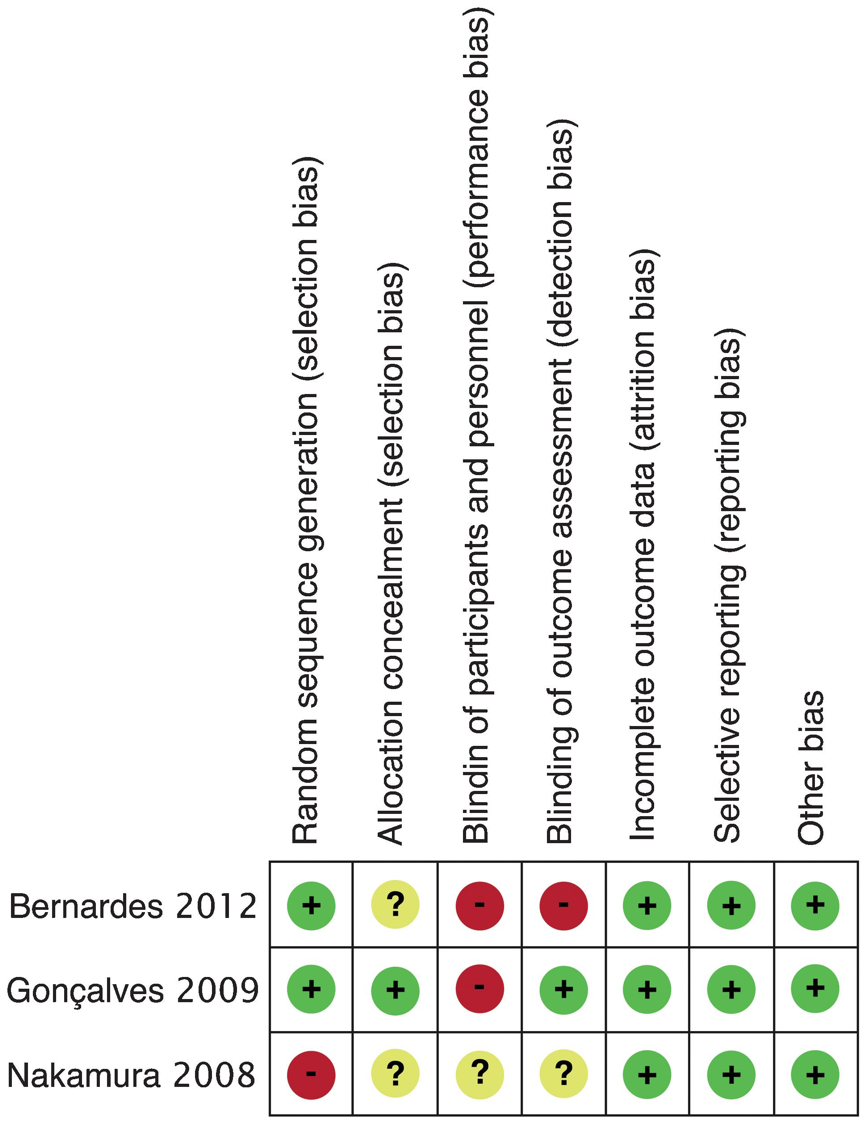 | 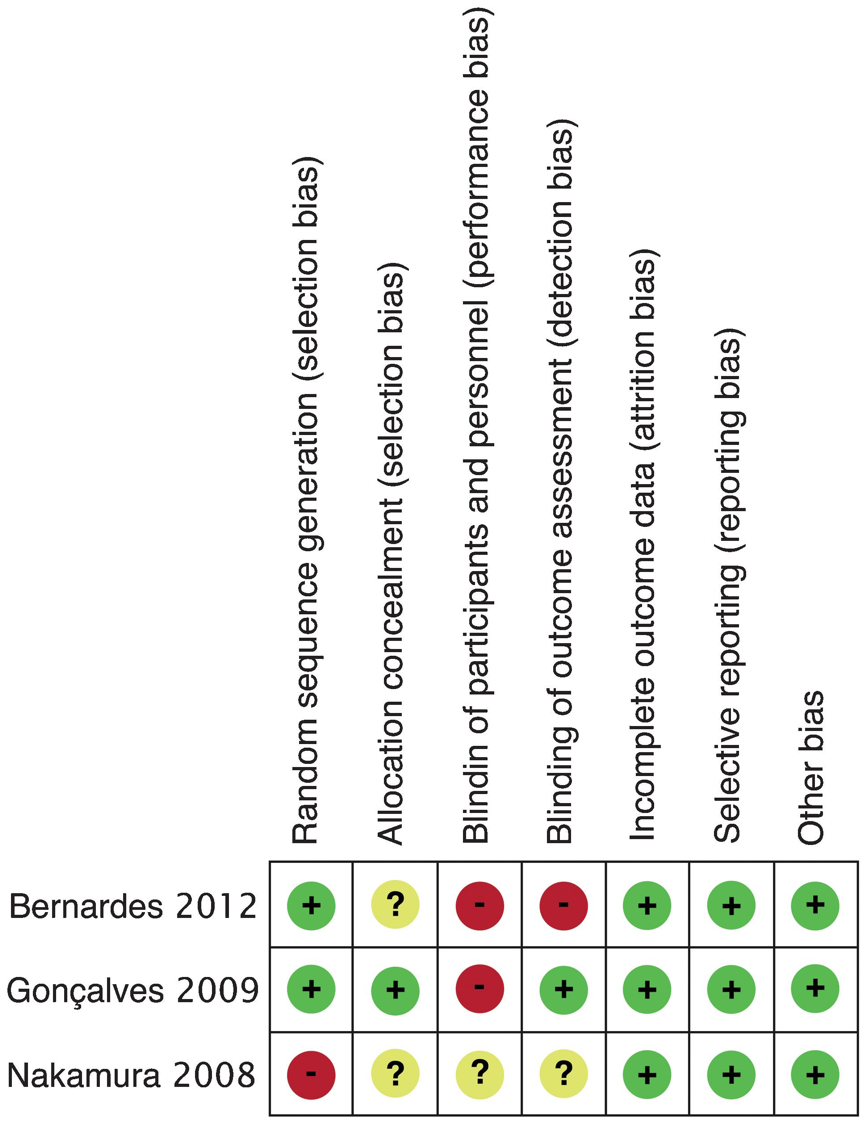 | 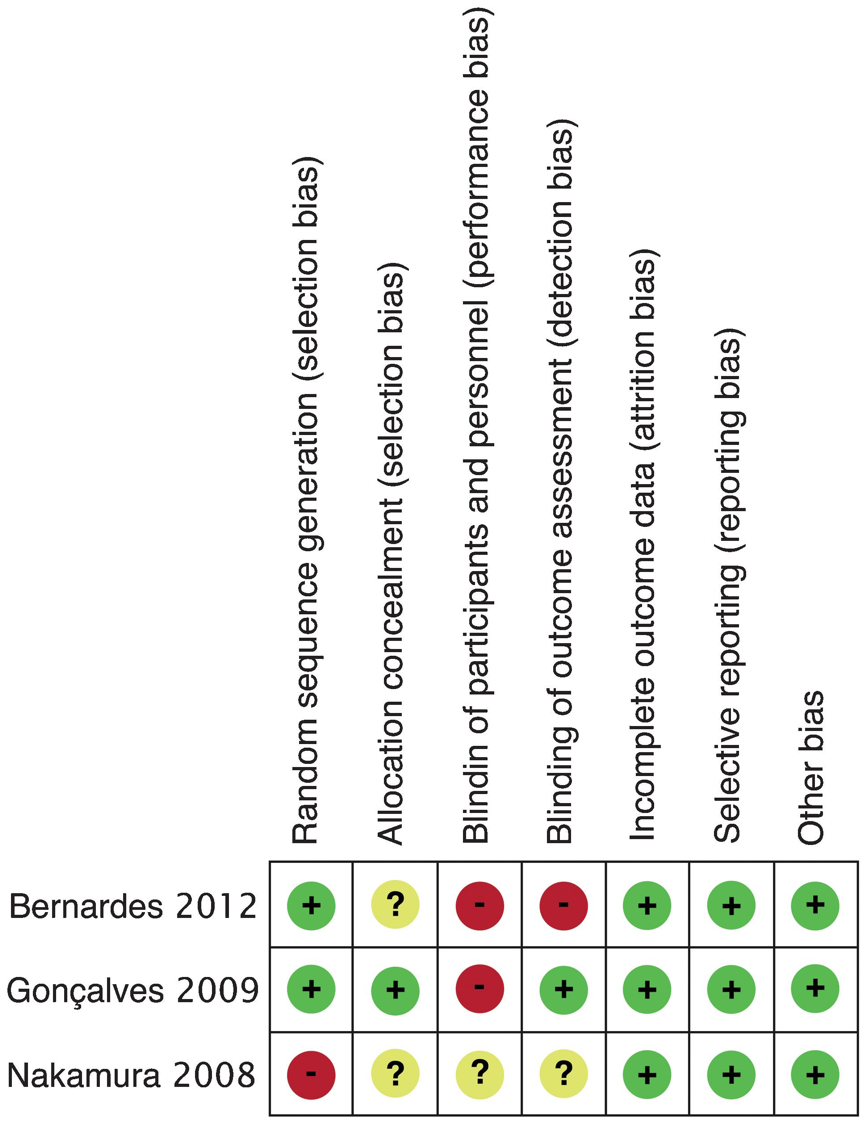 | 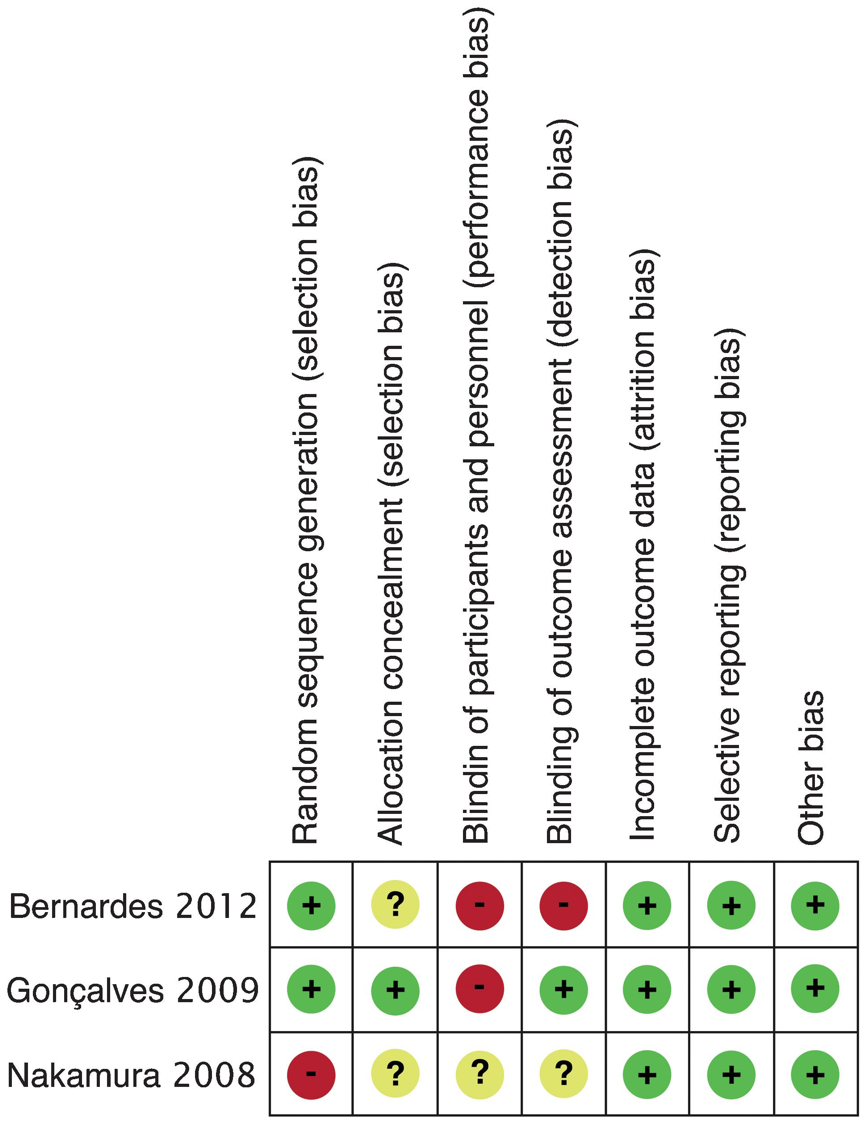 | 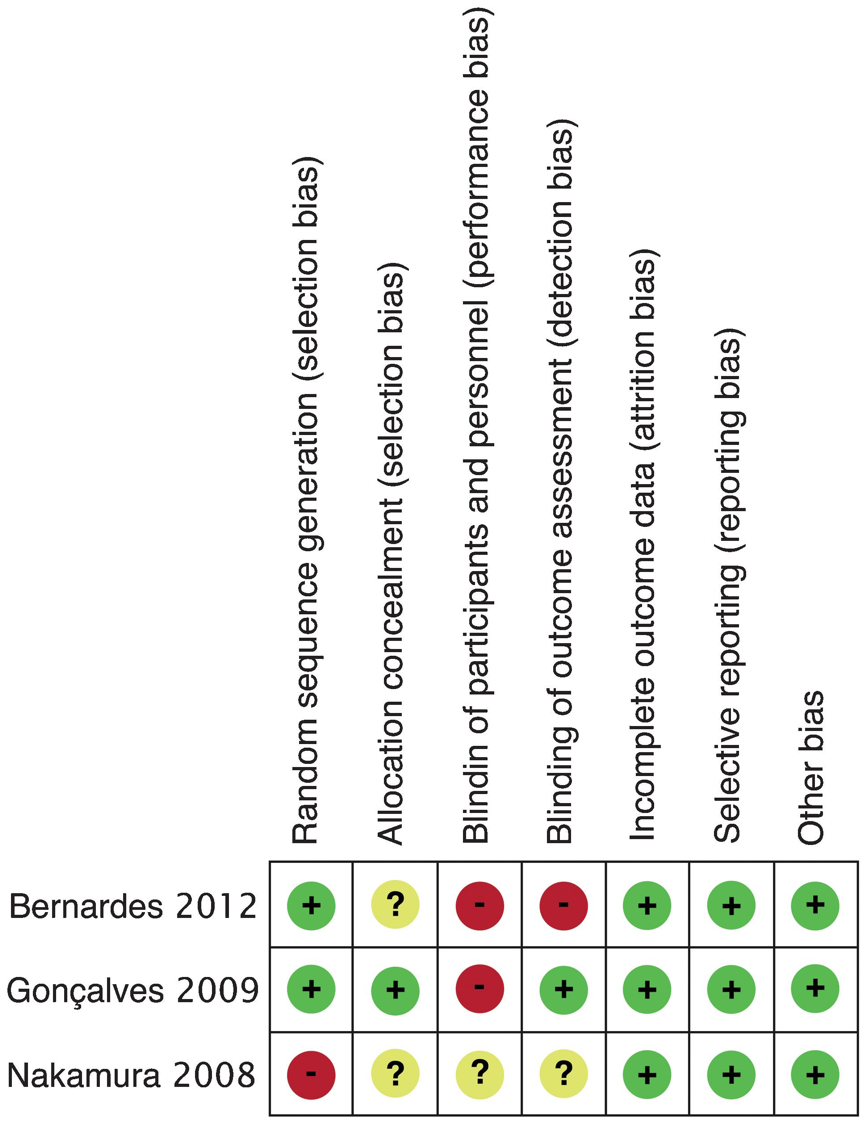 | 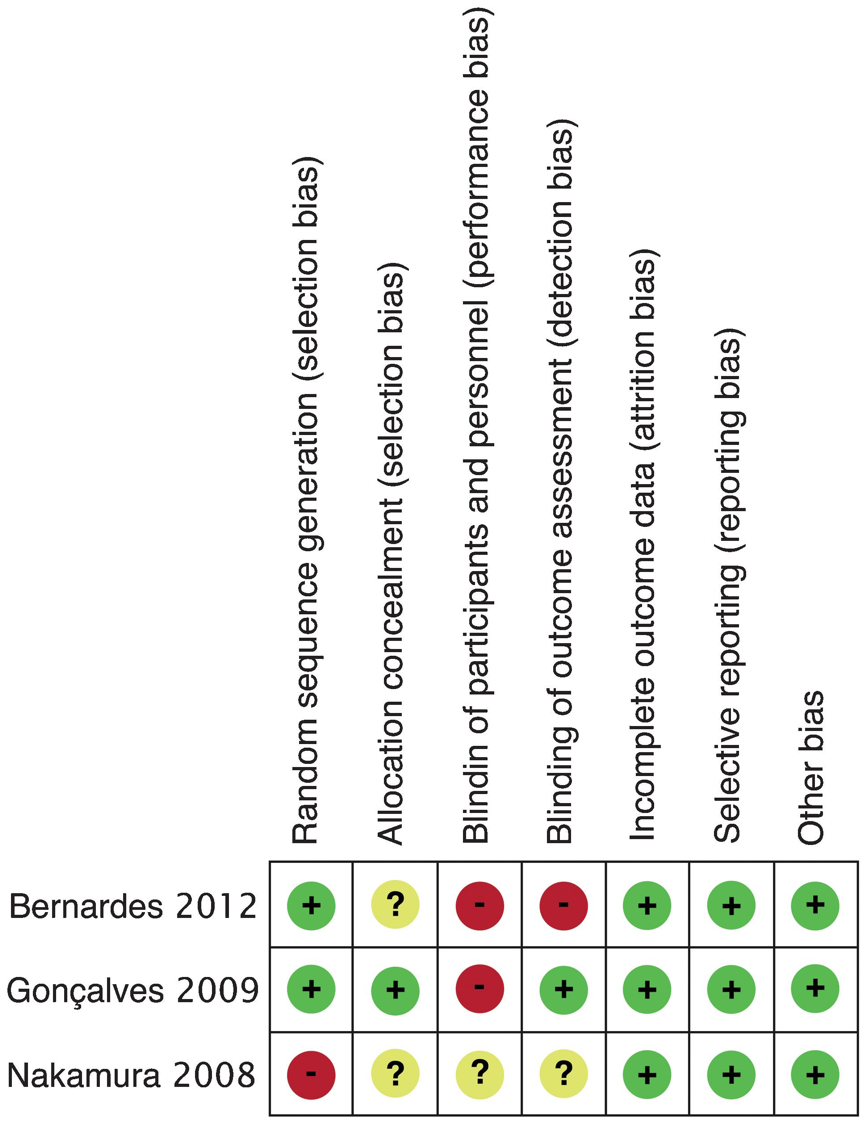 | 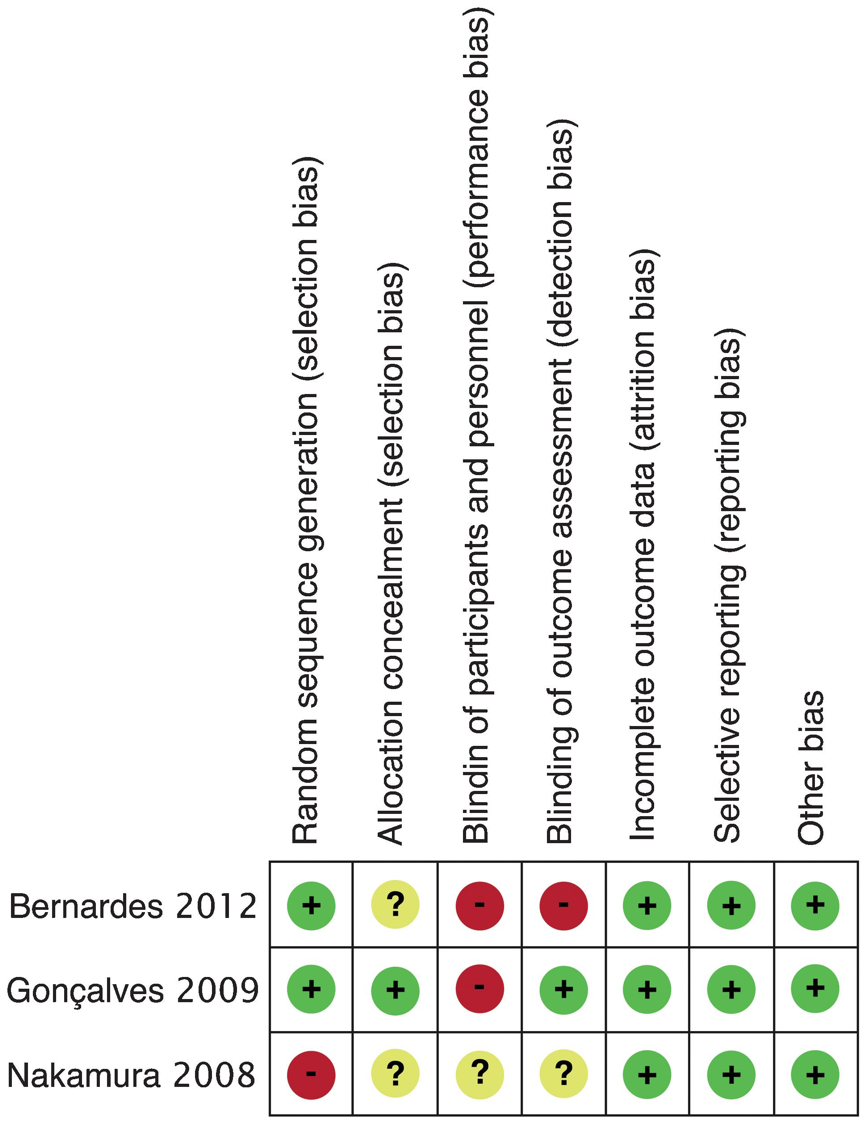 |
| Braakhuis et al. (2017) ^(24)^ | 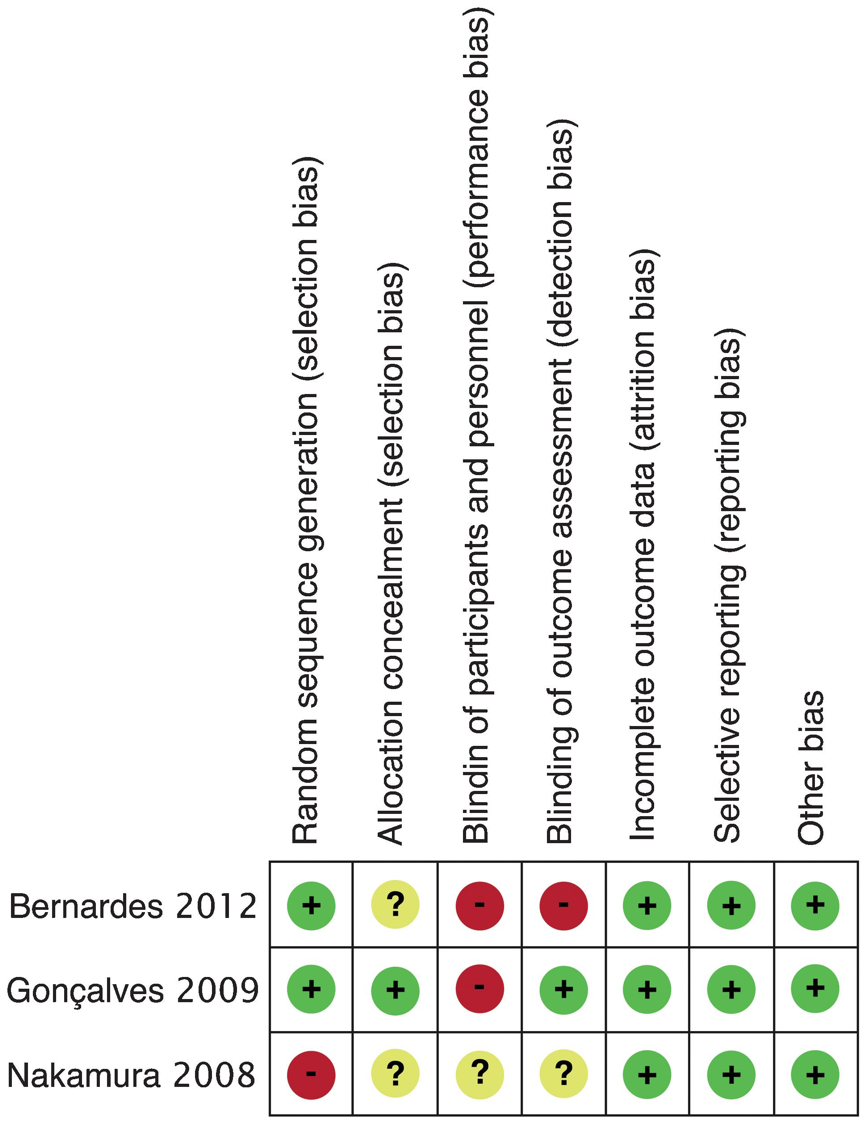 | 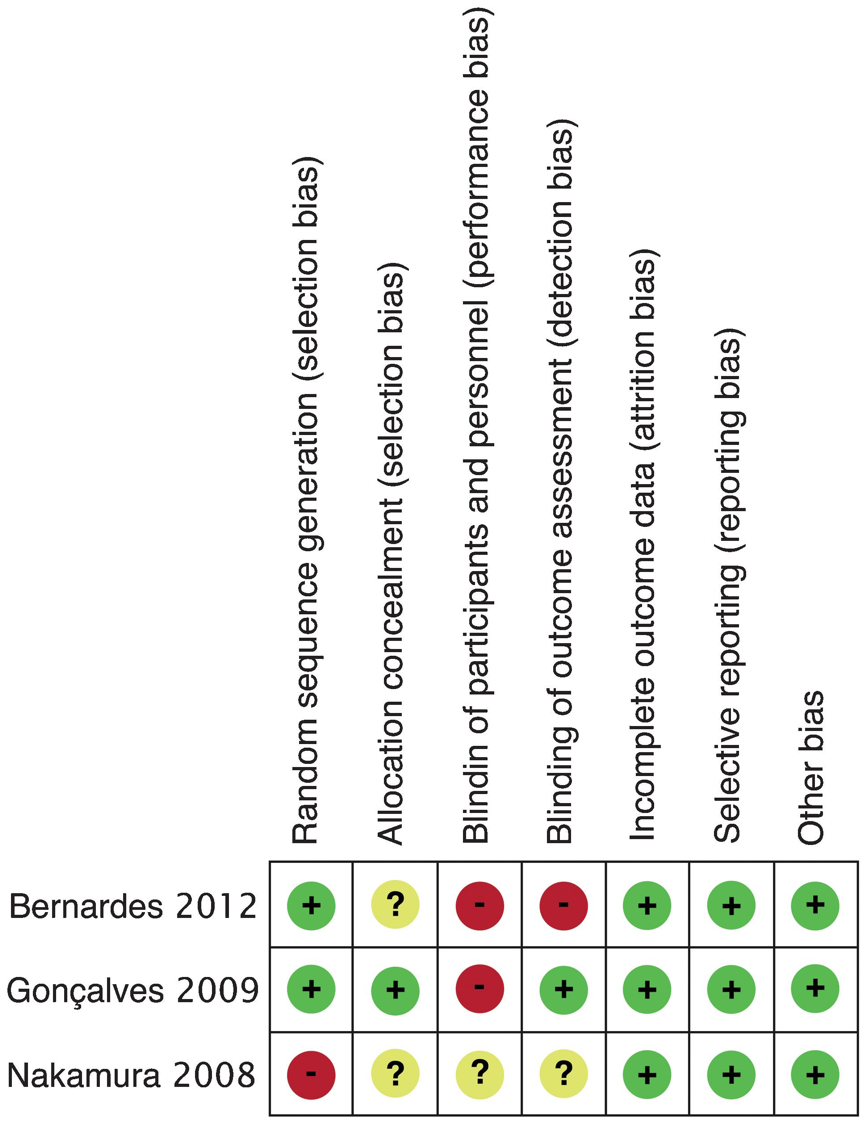 | 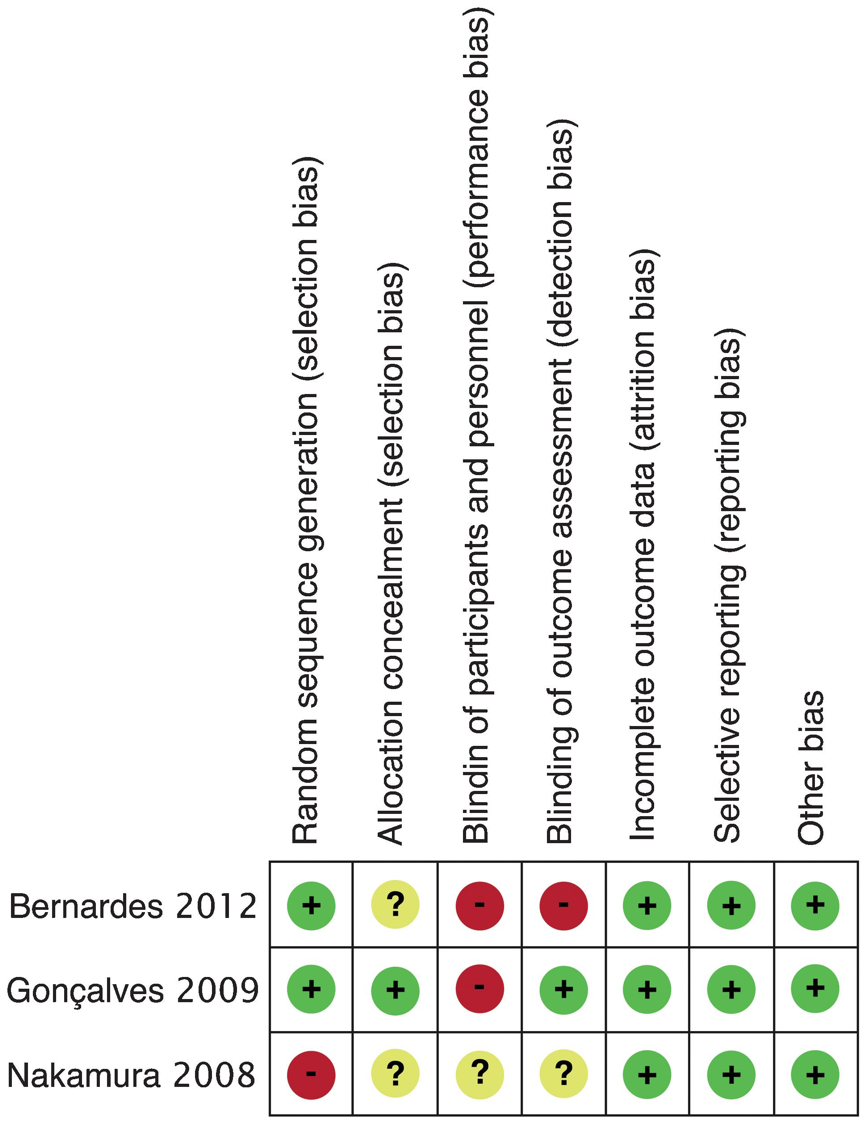 | 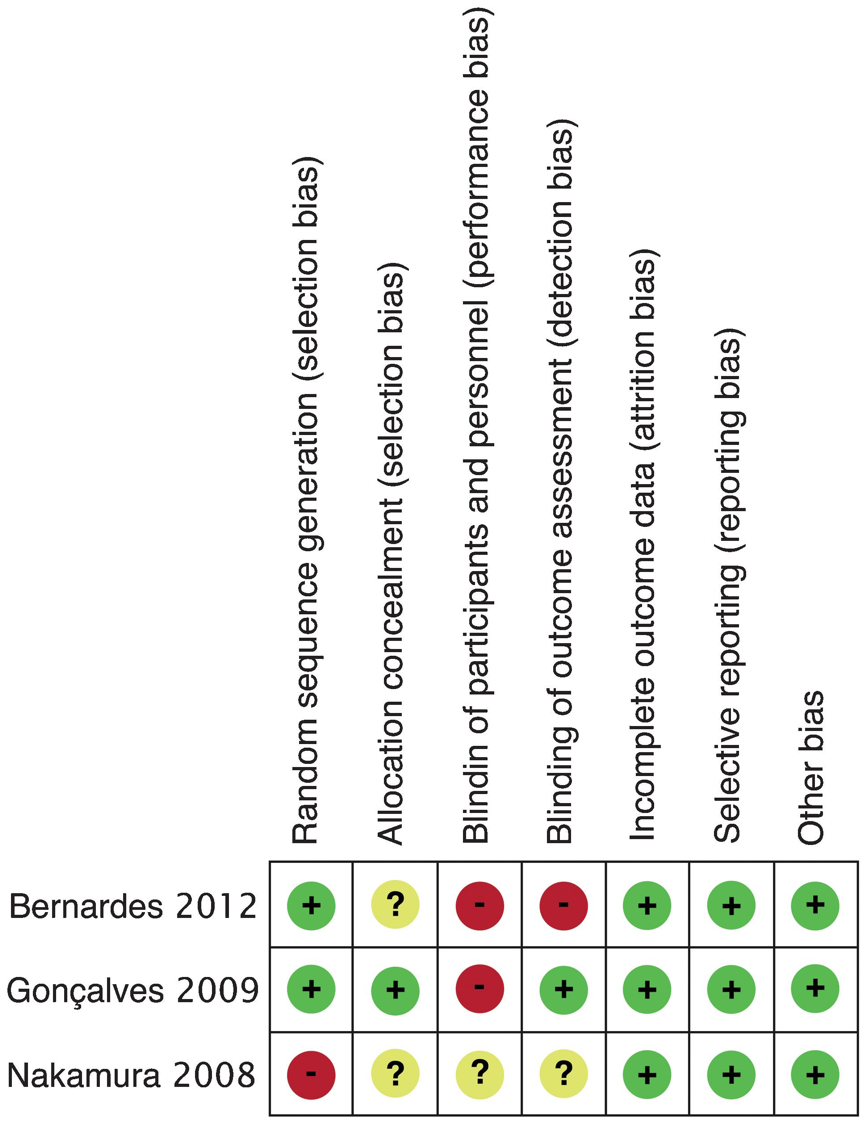 | 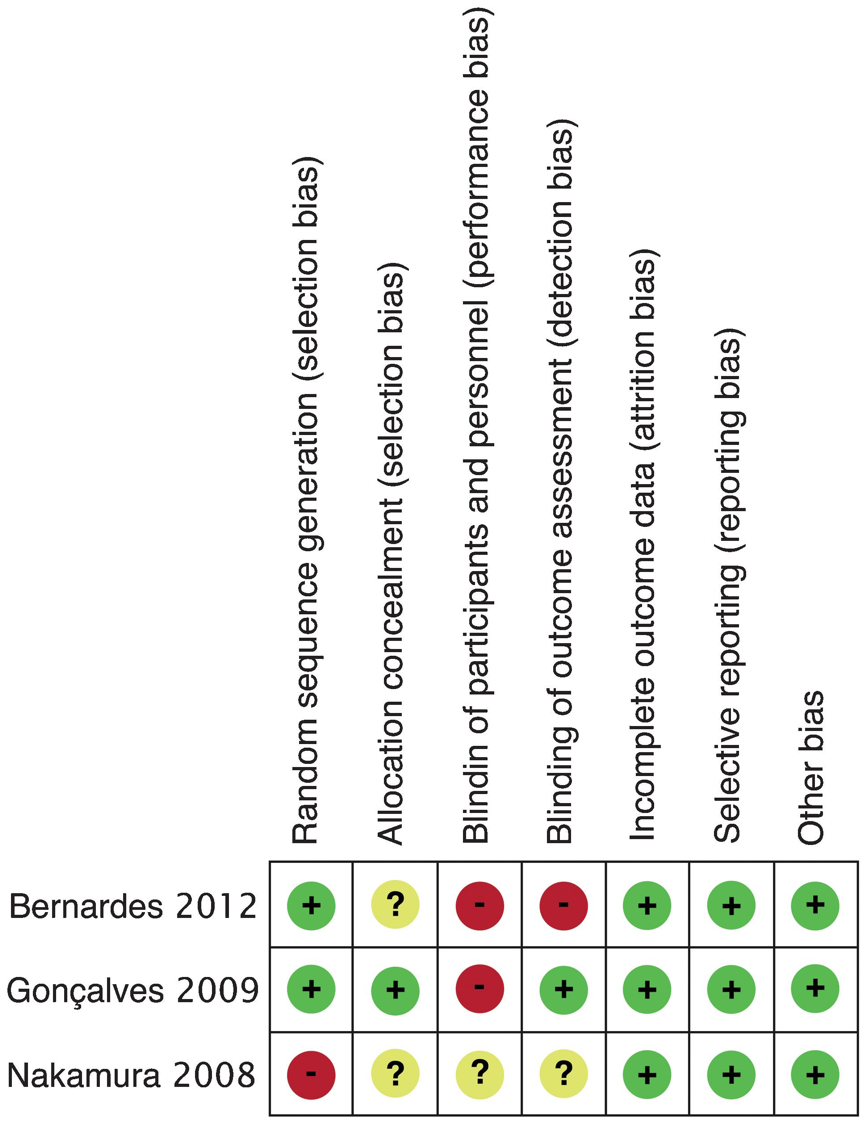 | 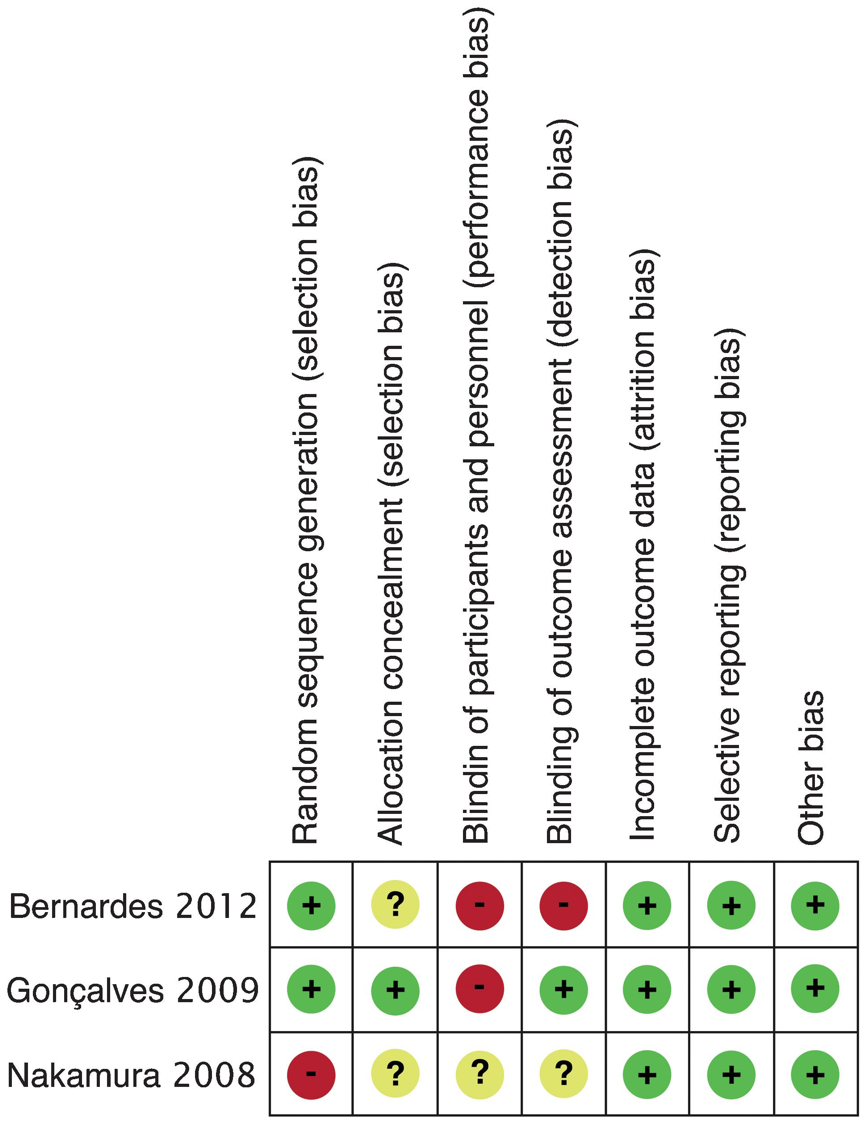 | 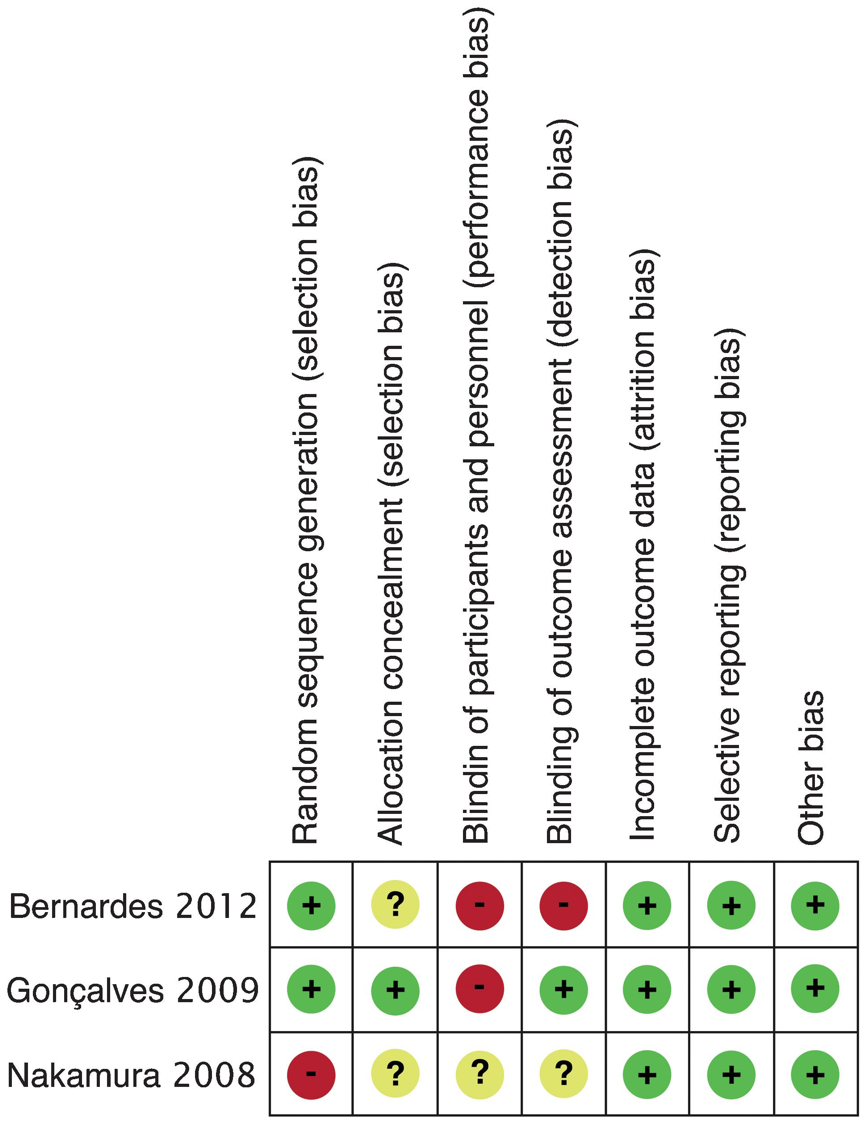 |
| Bruno et al. (2021) ^(25)^ | 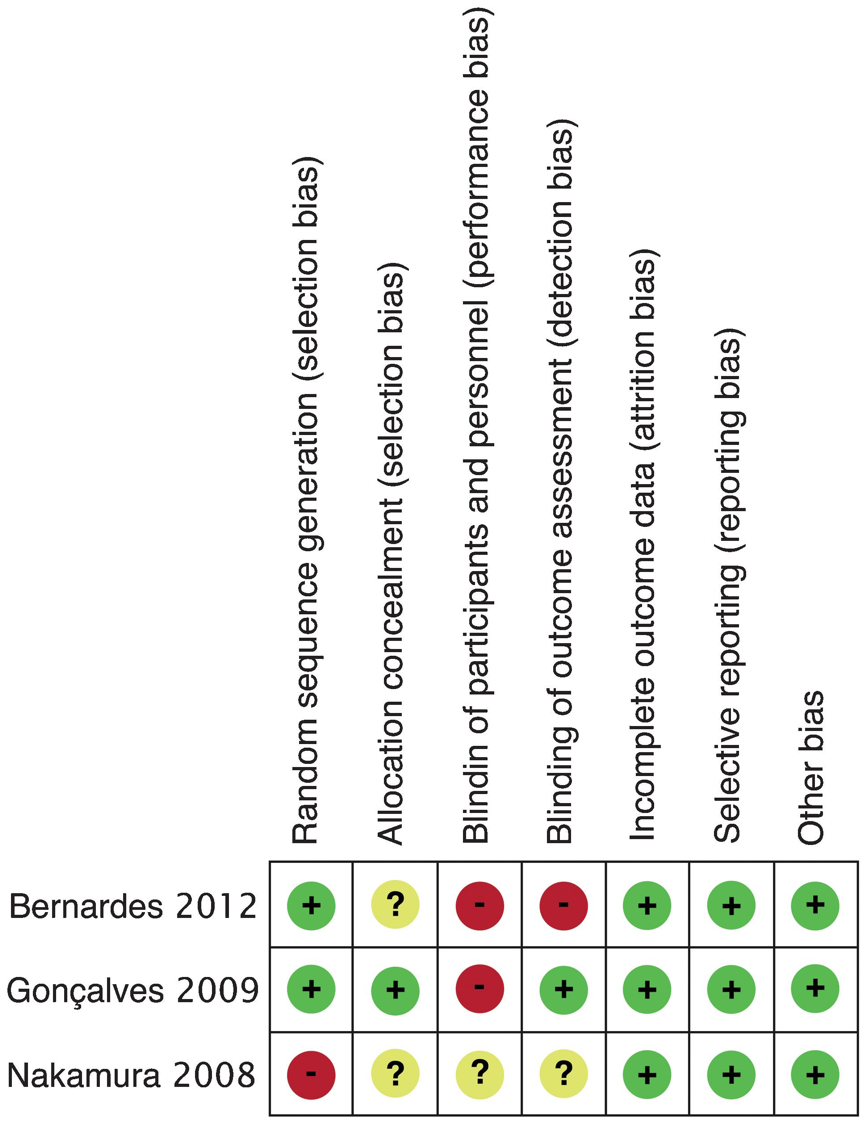 | 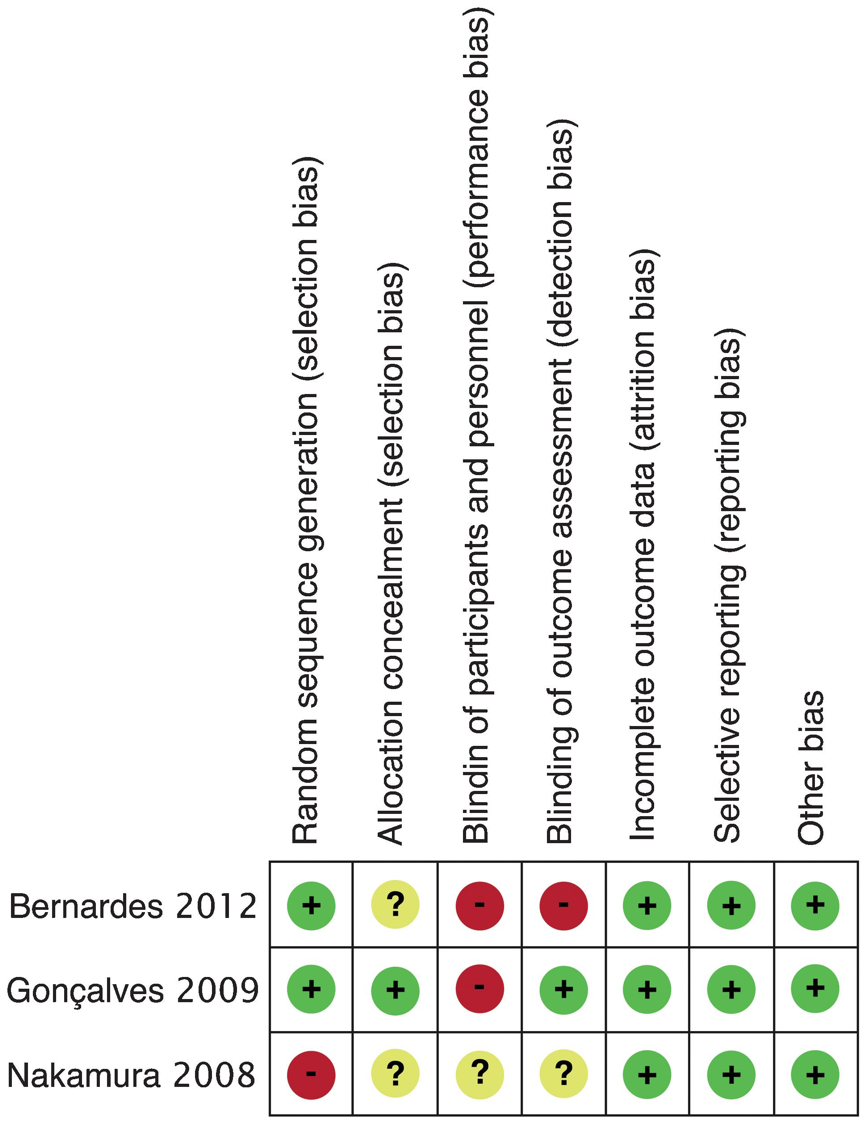 | 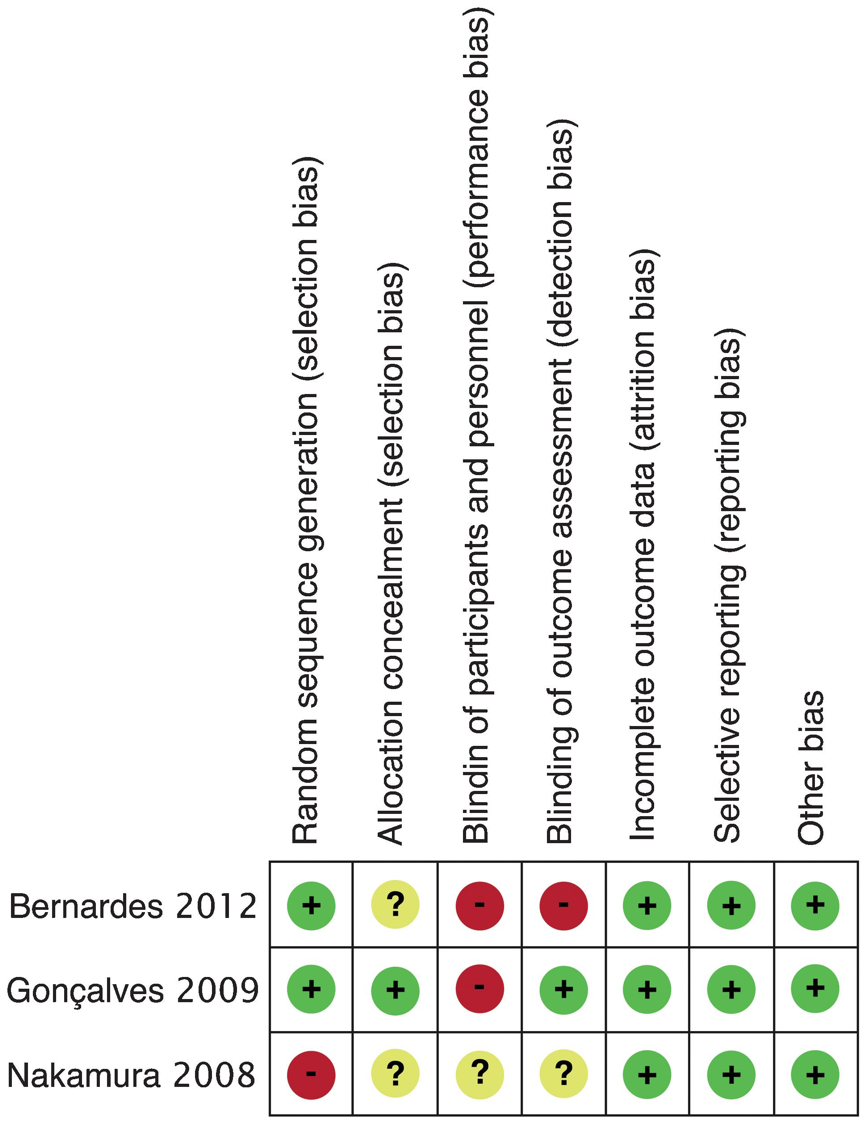 | 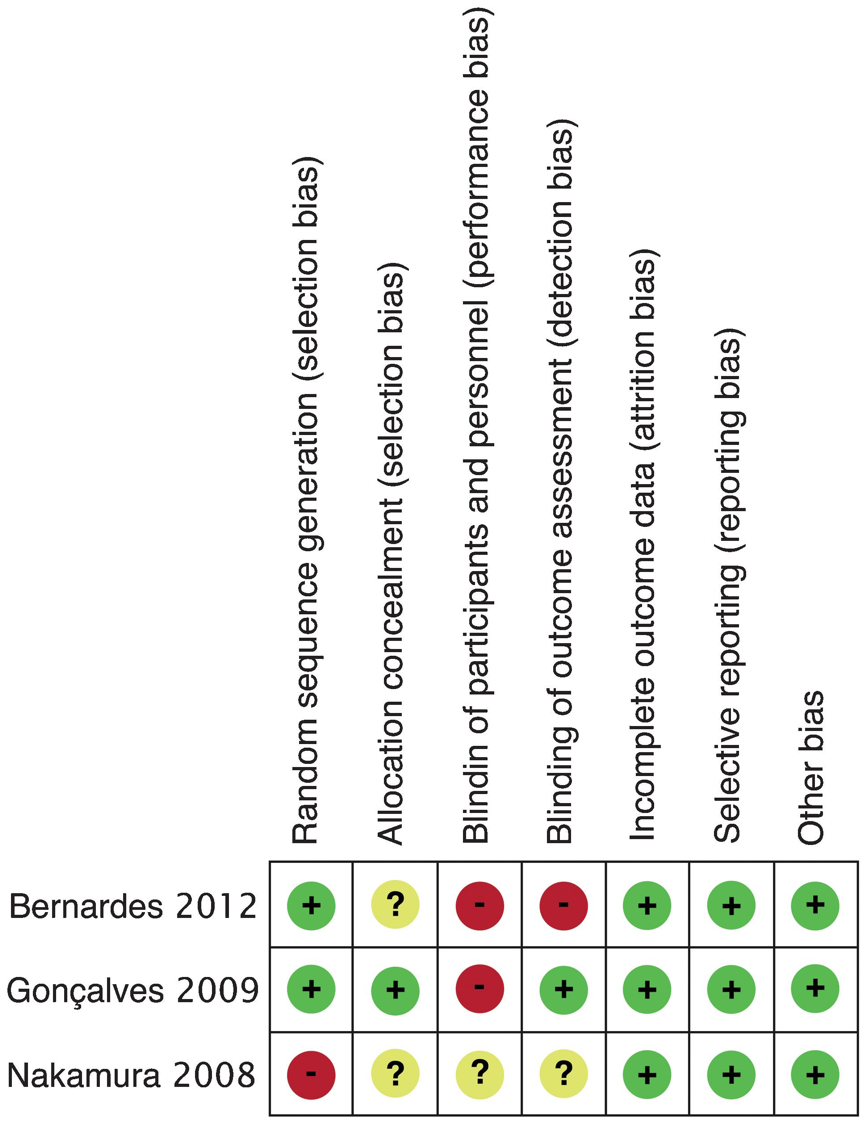 | 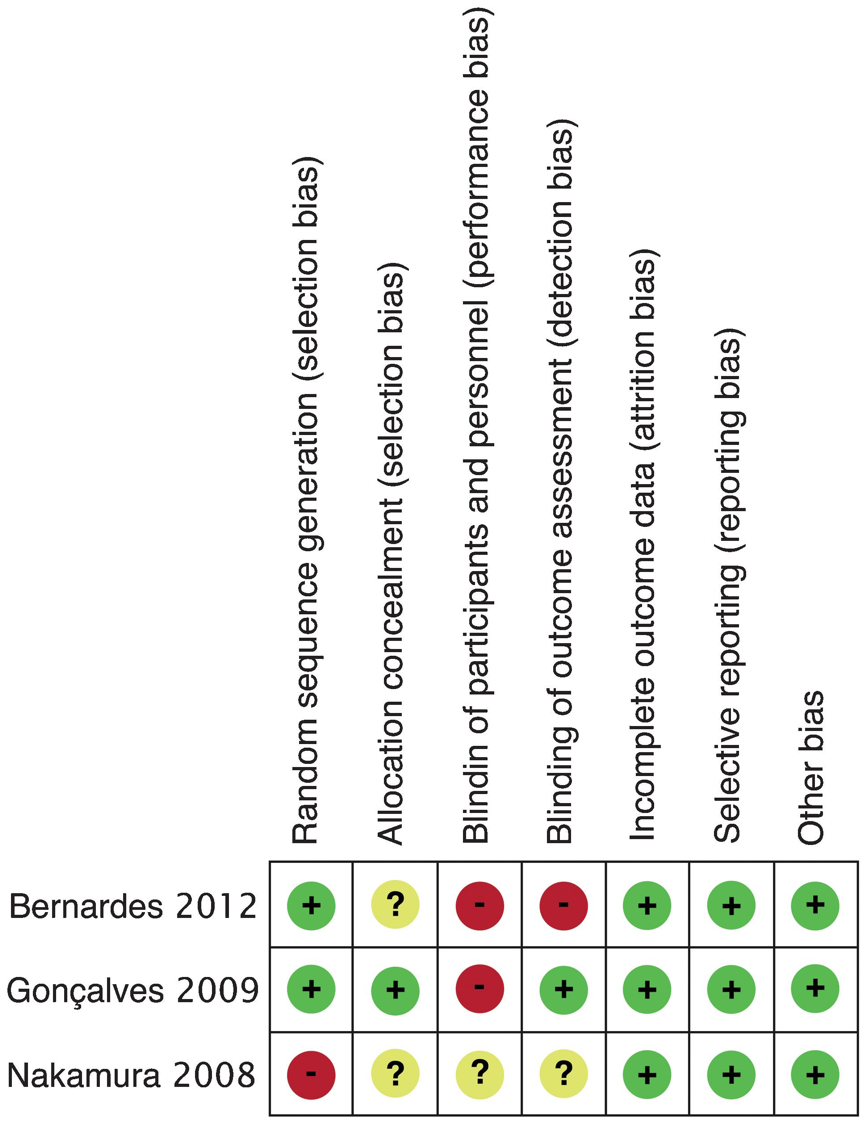 | 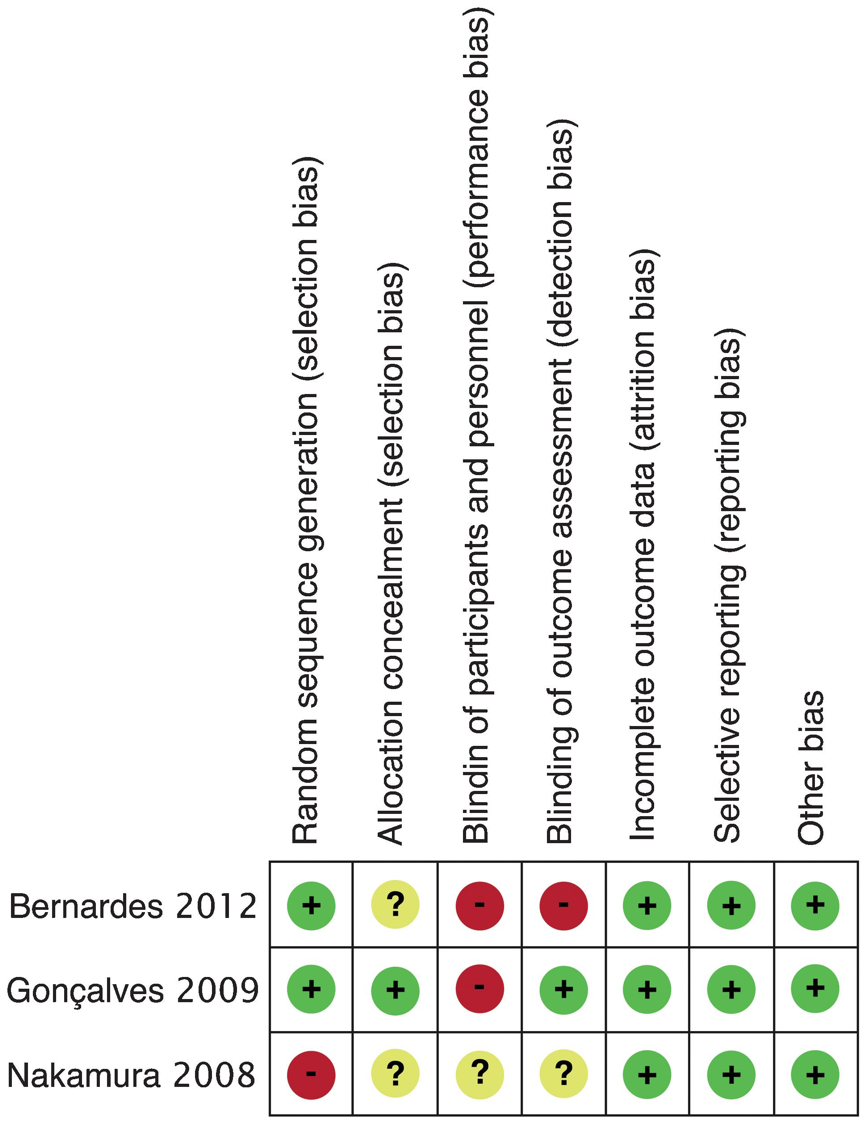 | 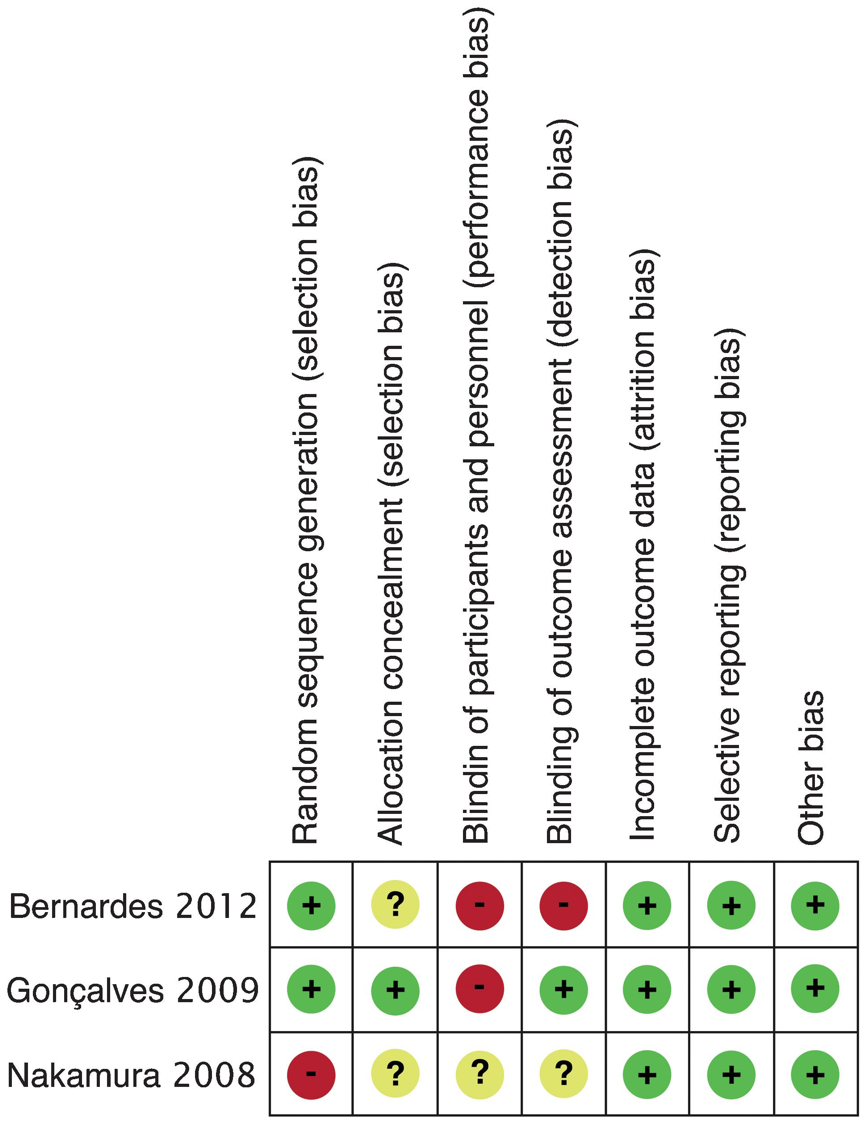 |
| Cho et al. (2022) ^(26)^ | 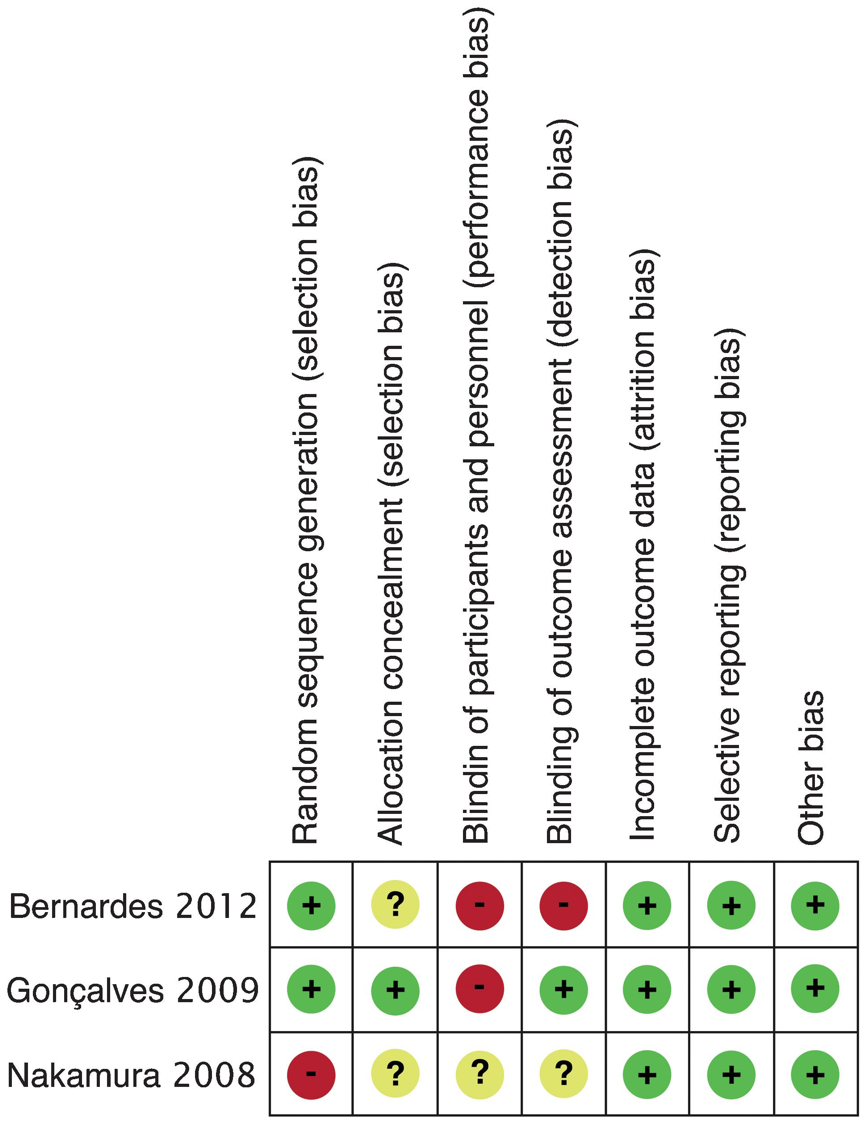 | 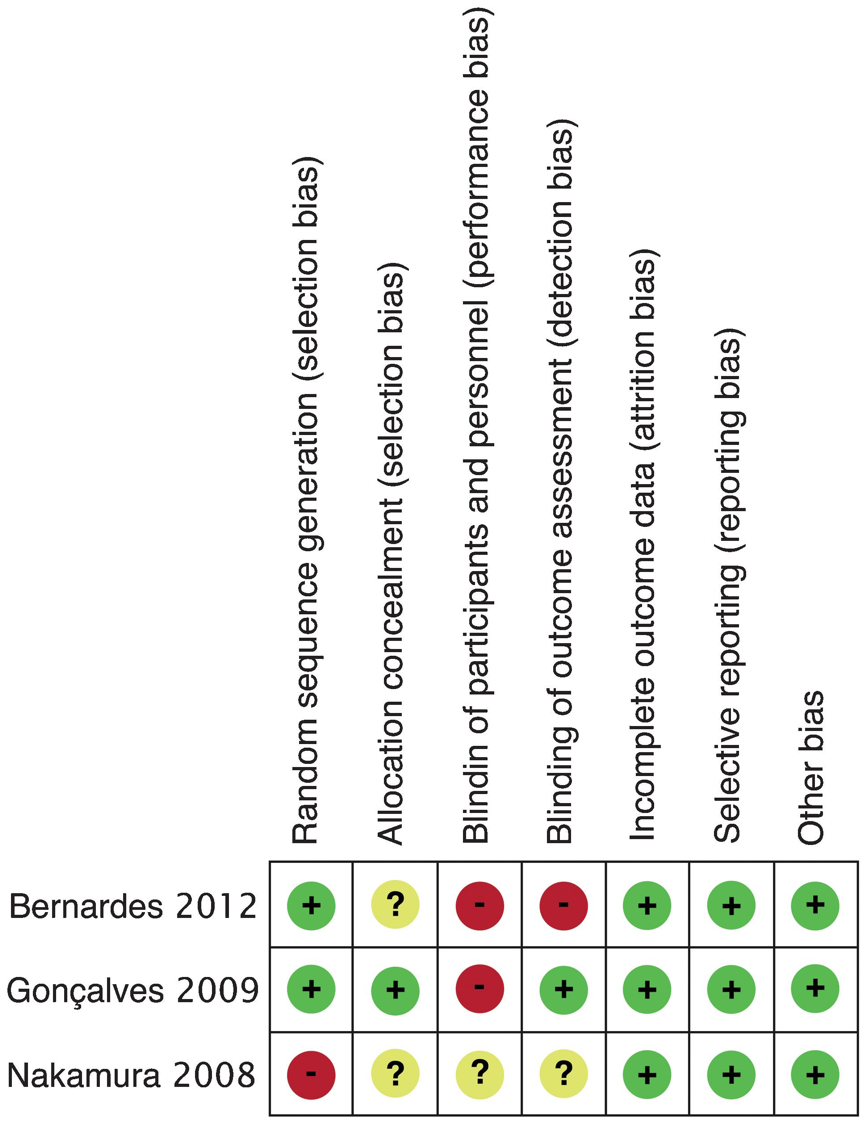 | 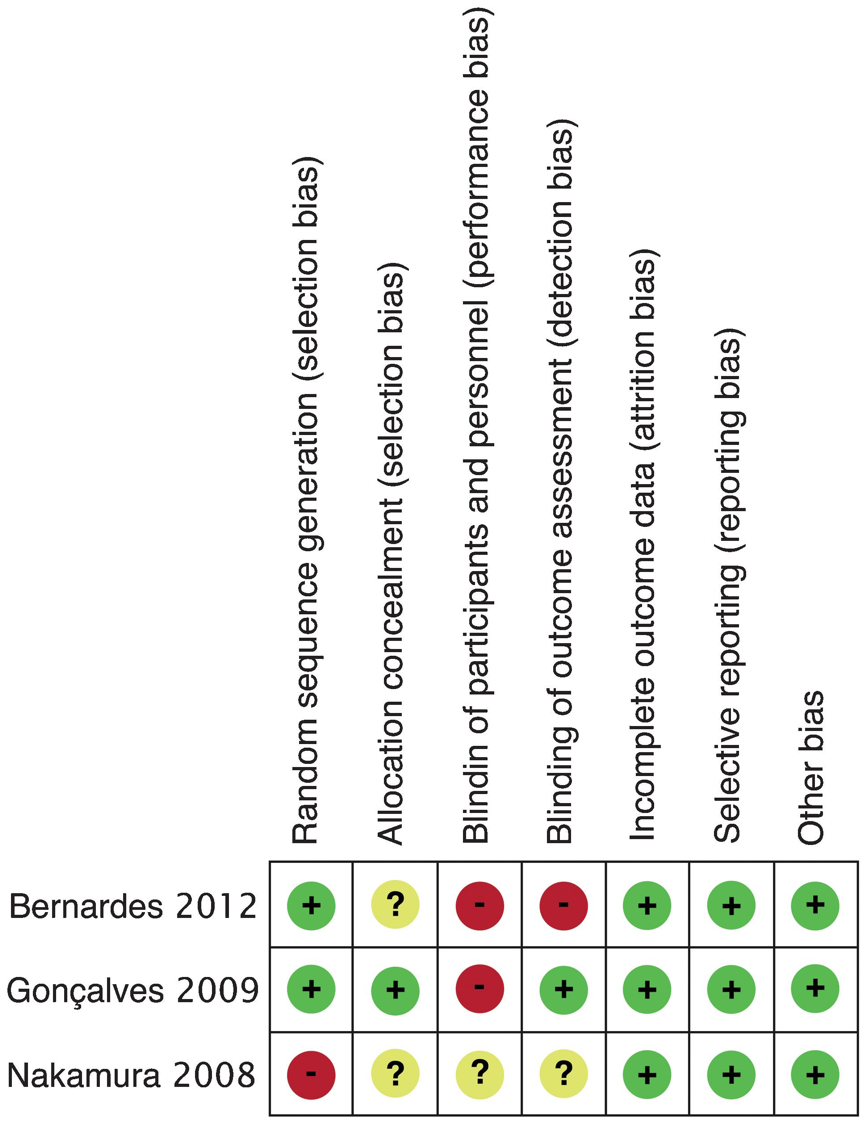 | 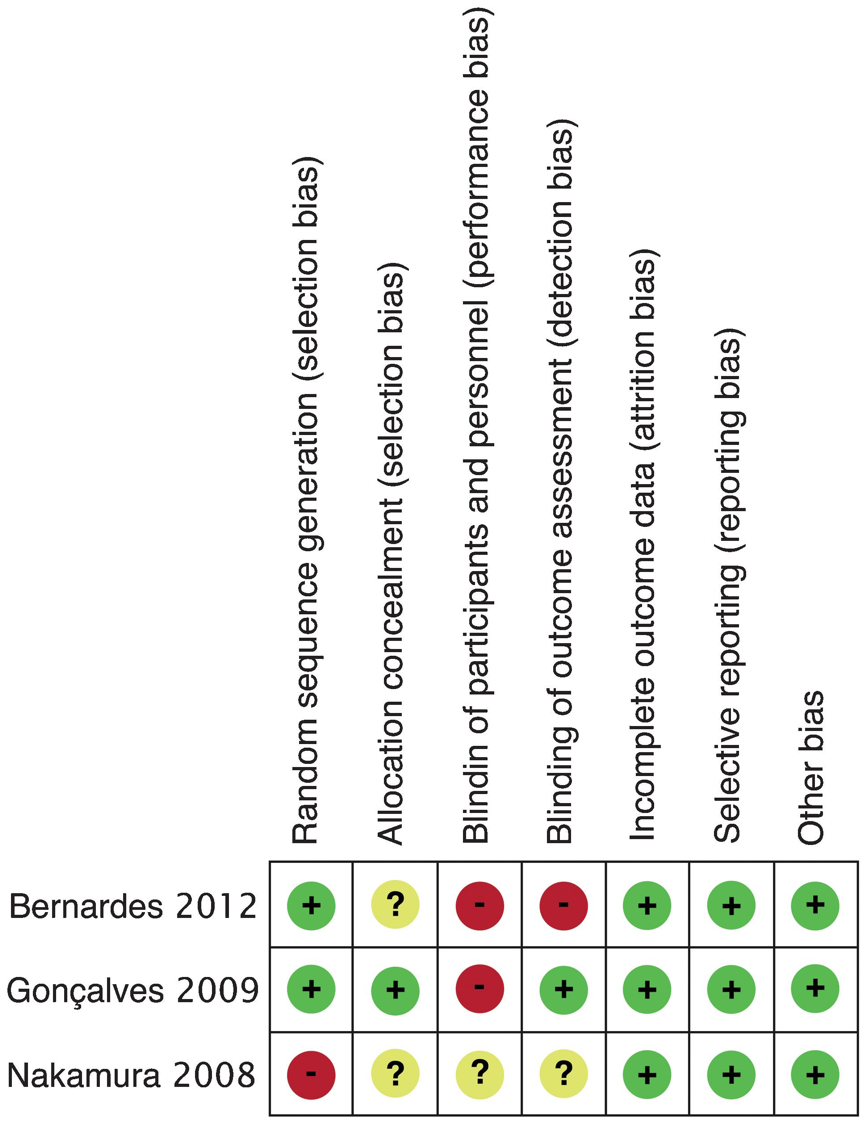 | 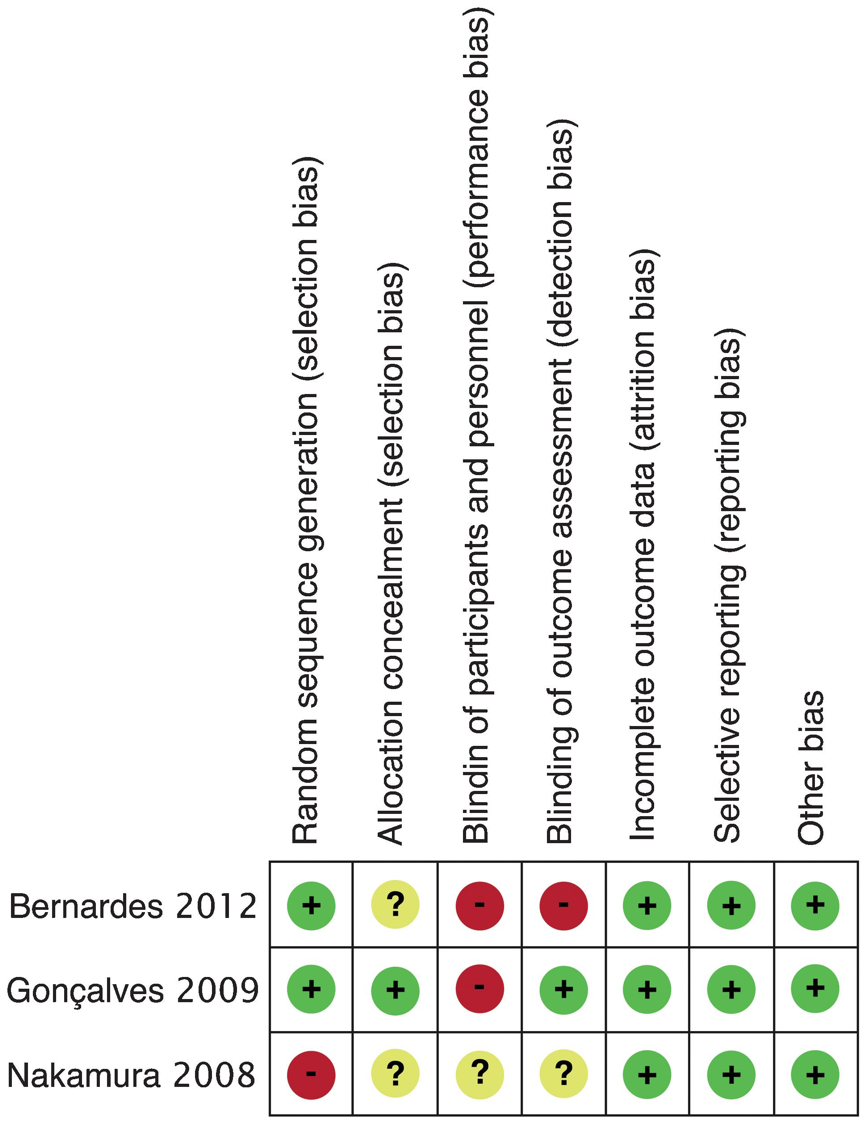 | 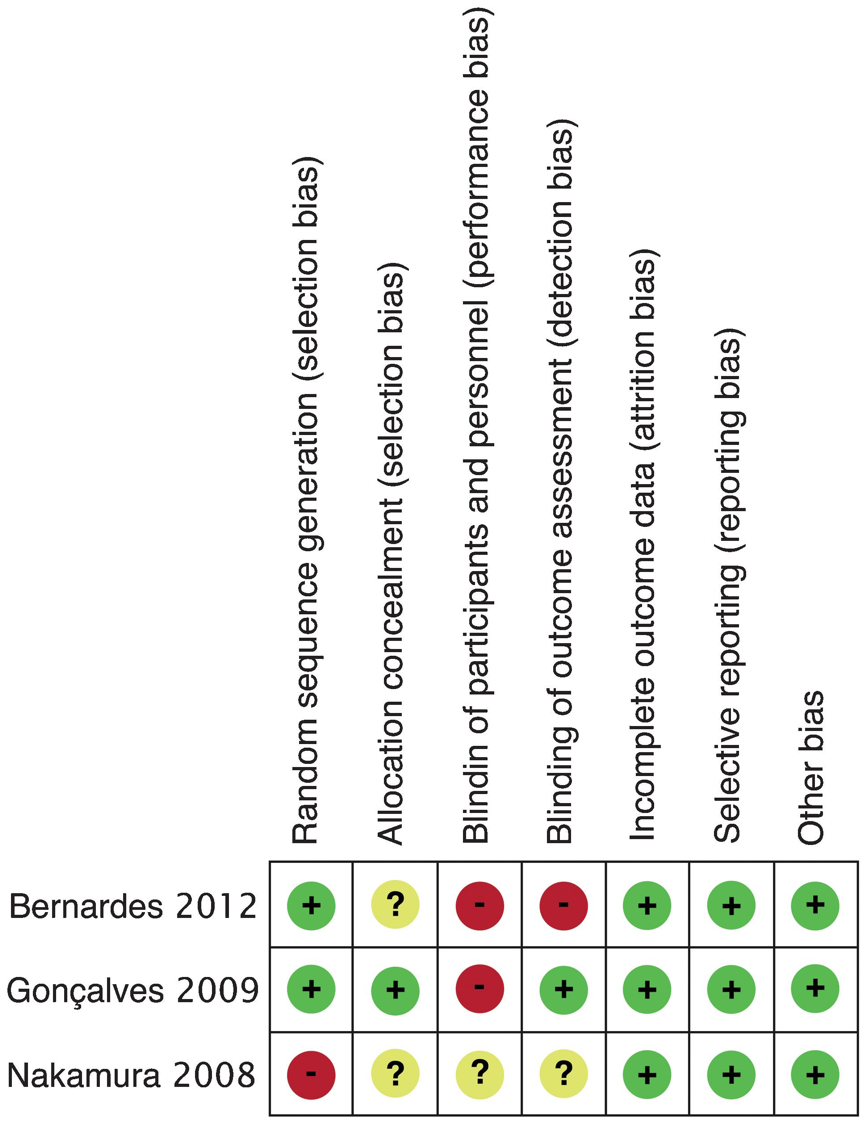 | 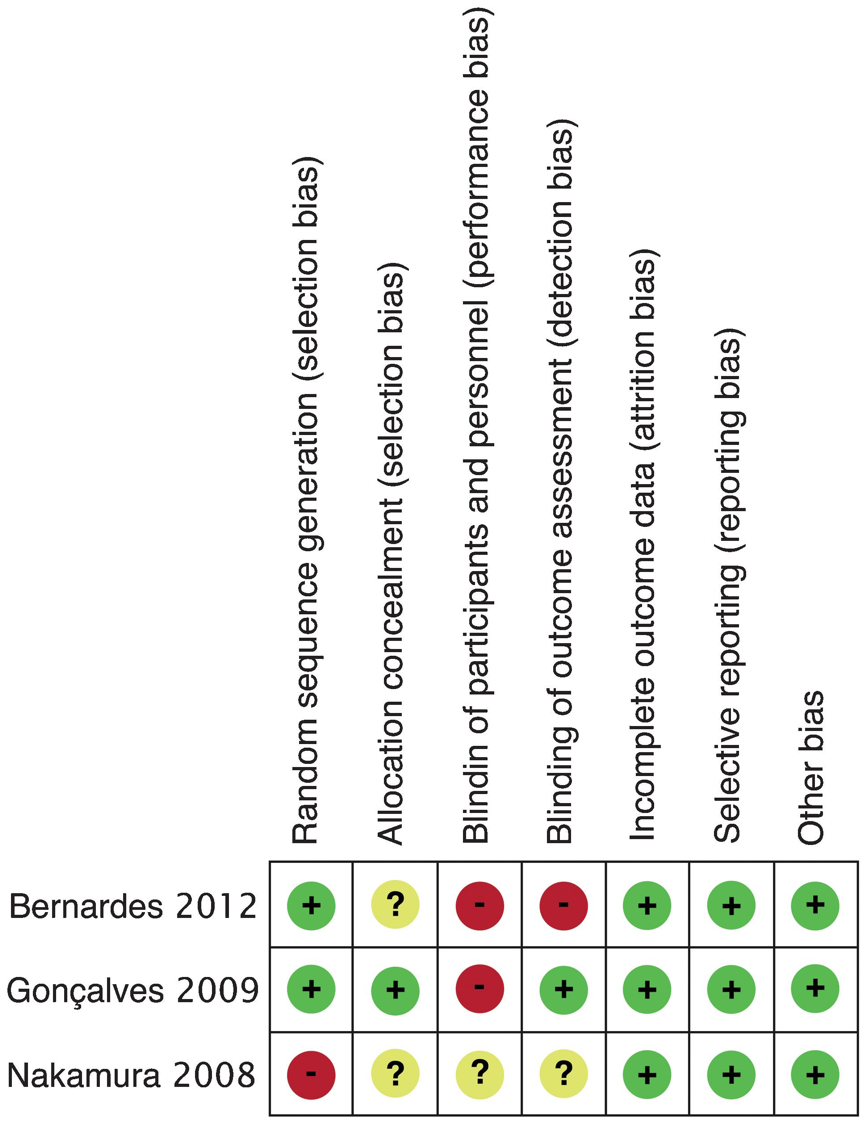 |
| Gioxari et al. (2021) ^(27)^ | 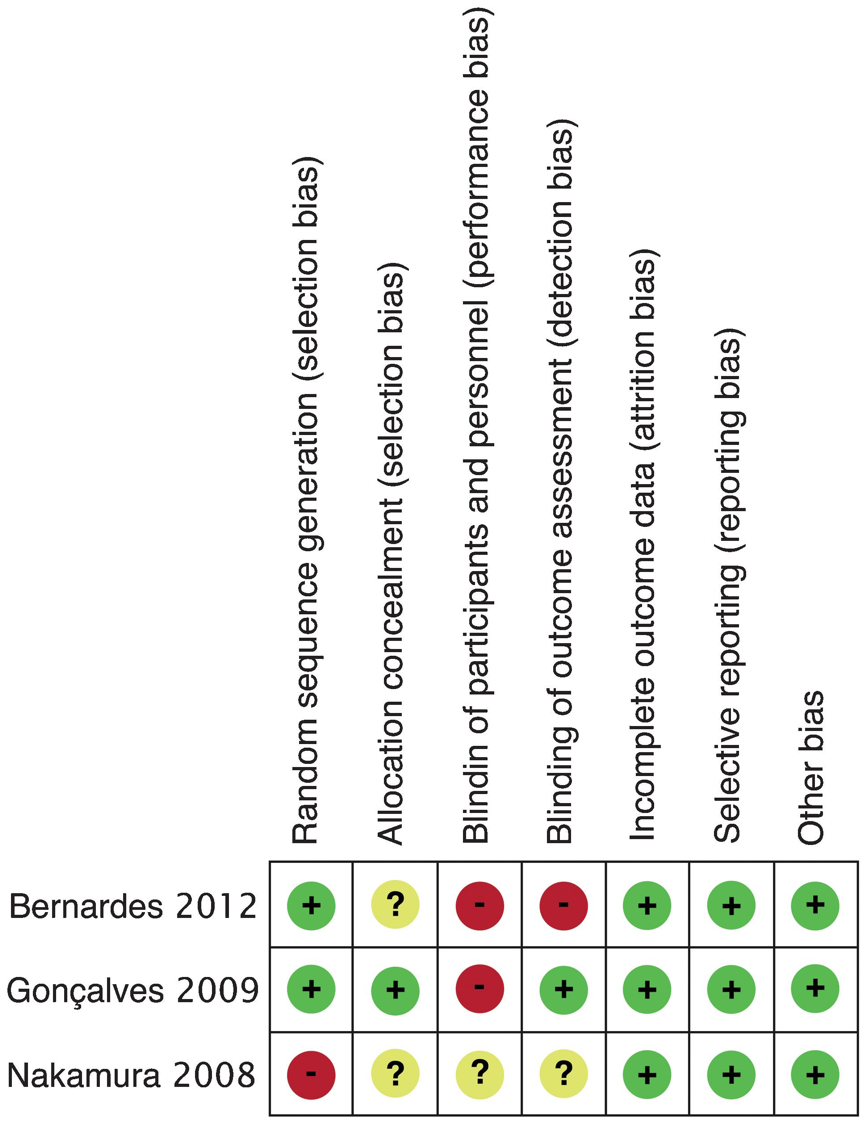 | 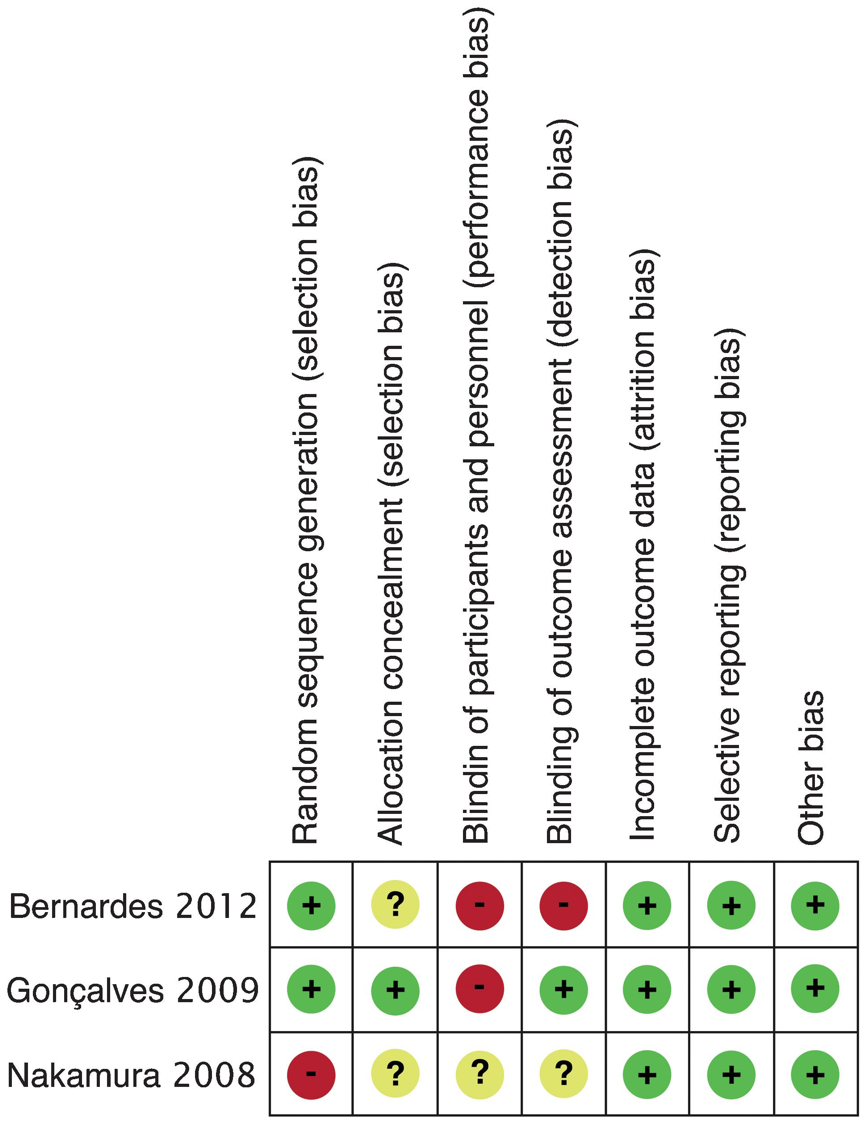 | 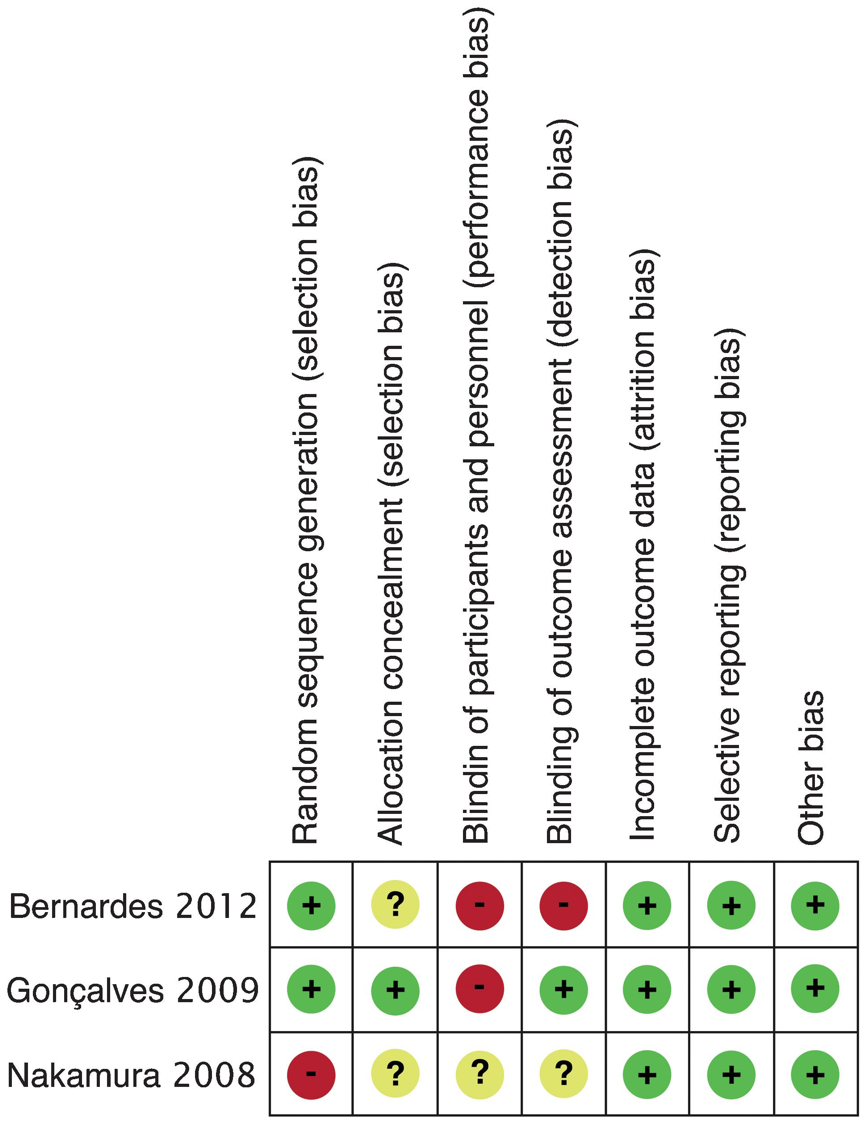 | 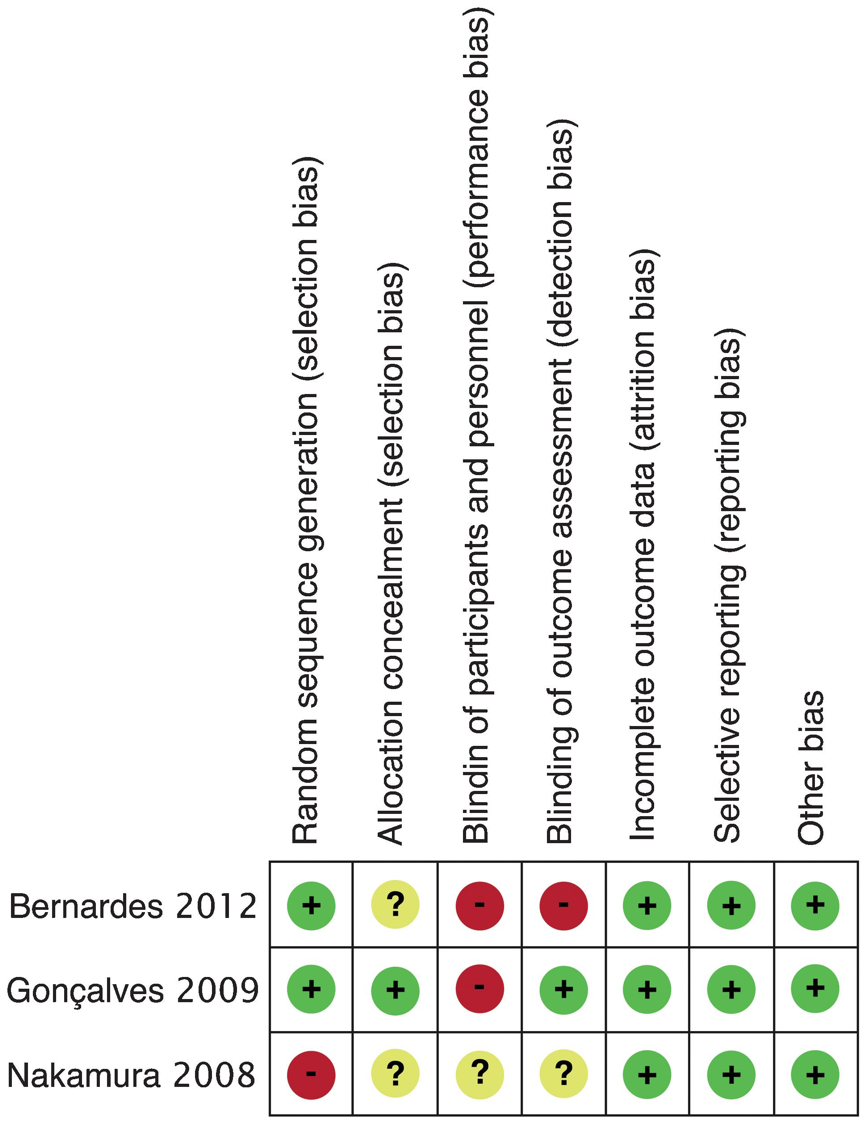 | 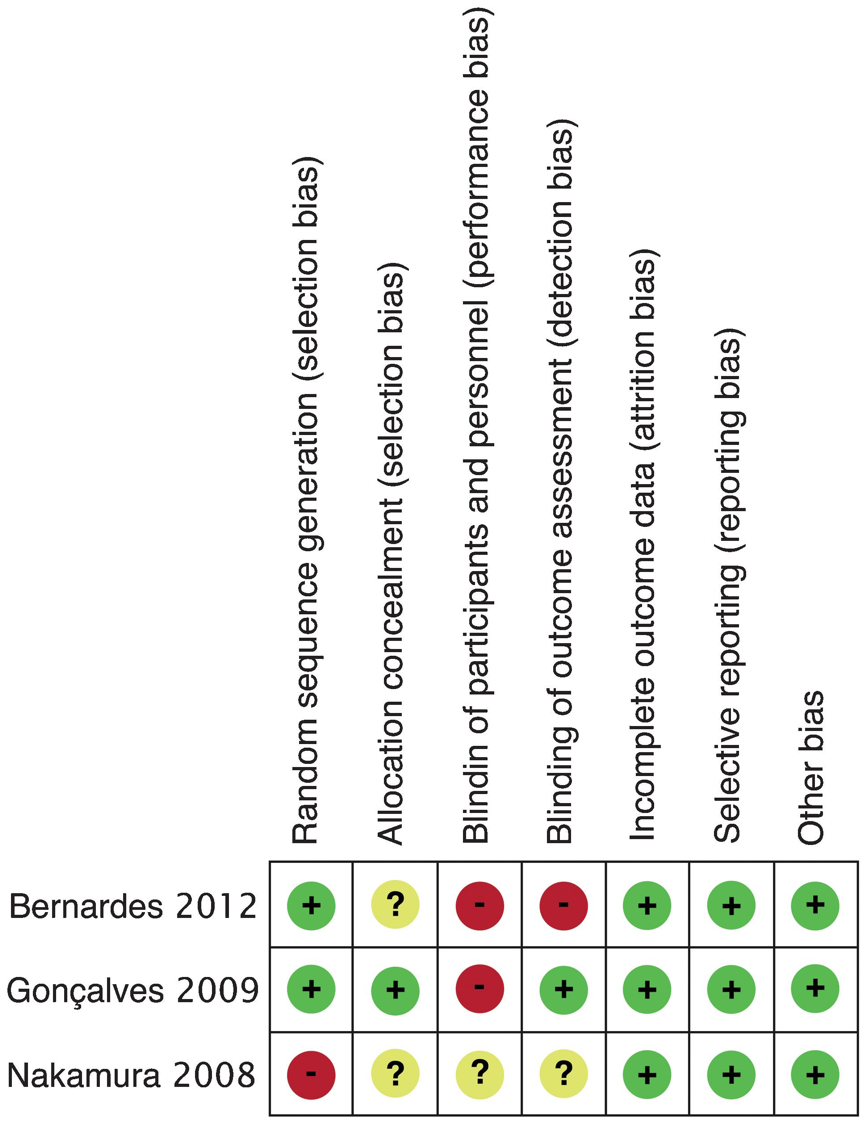 | 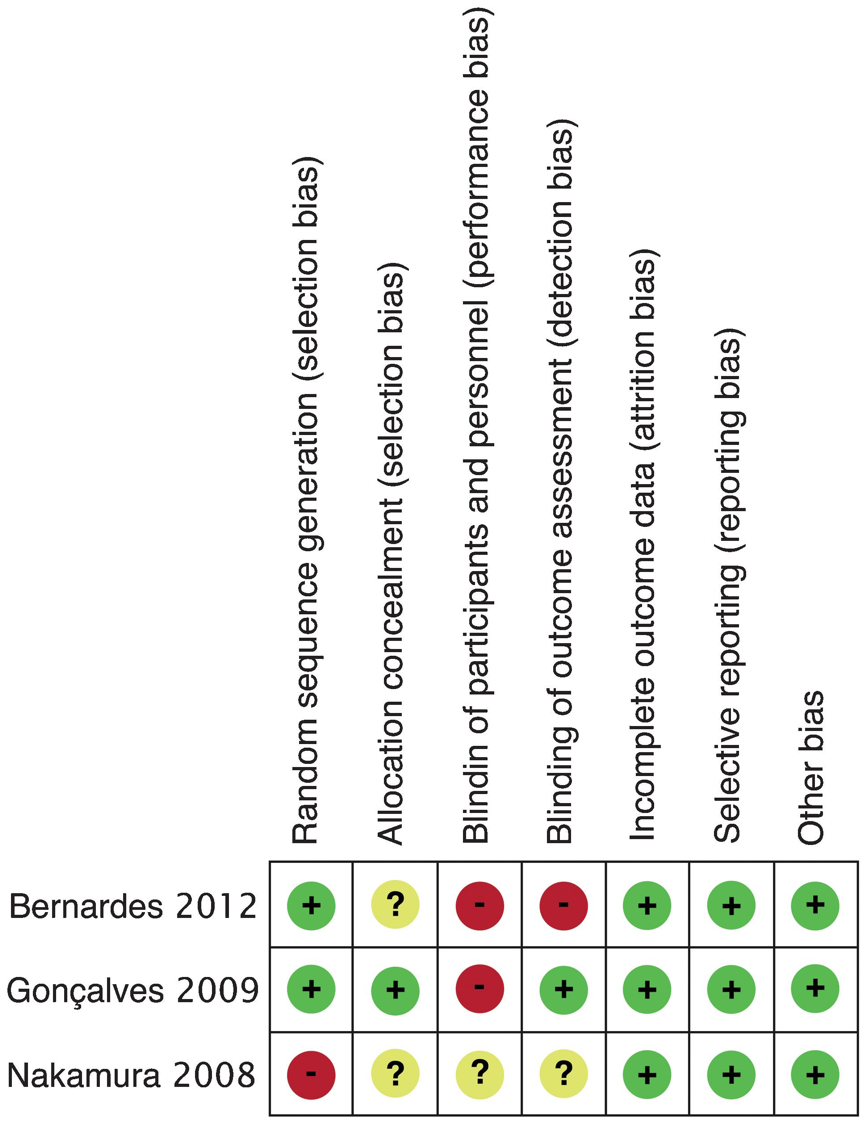 | 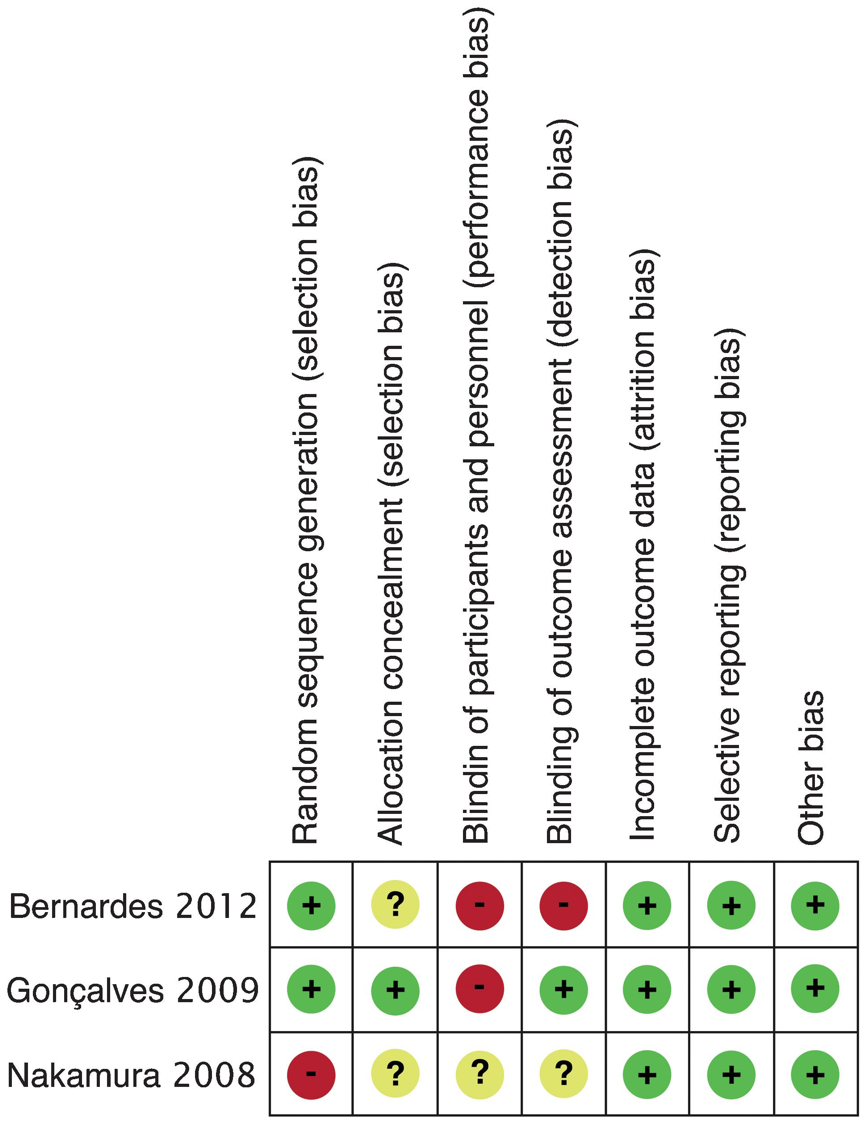 |
| Harvie et al. (2019) ^(28)^ | 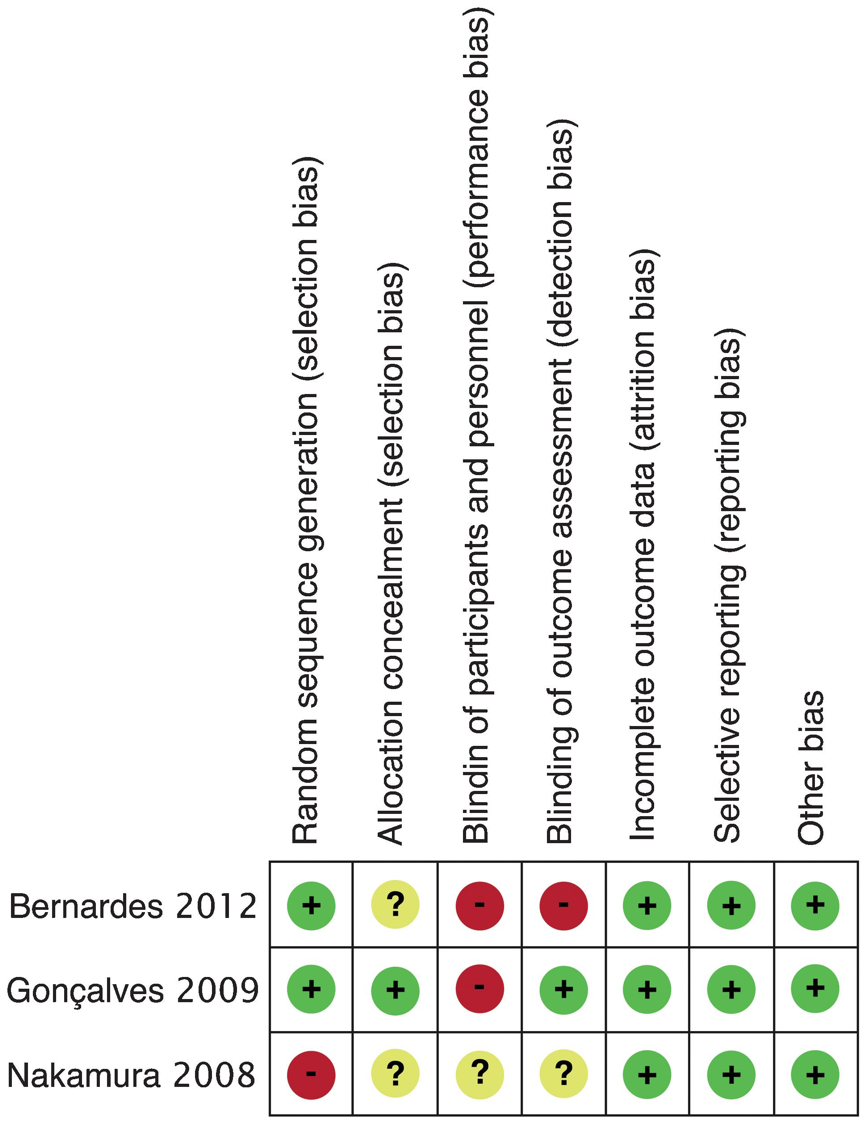 | 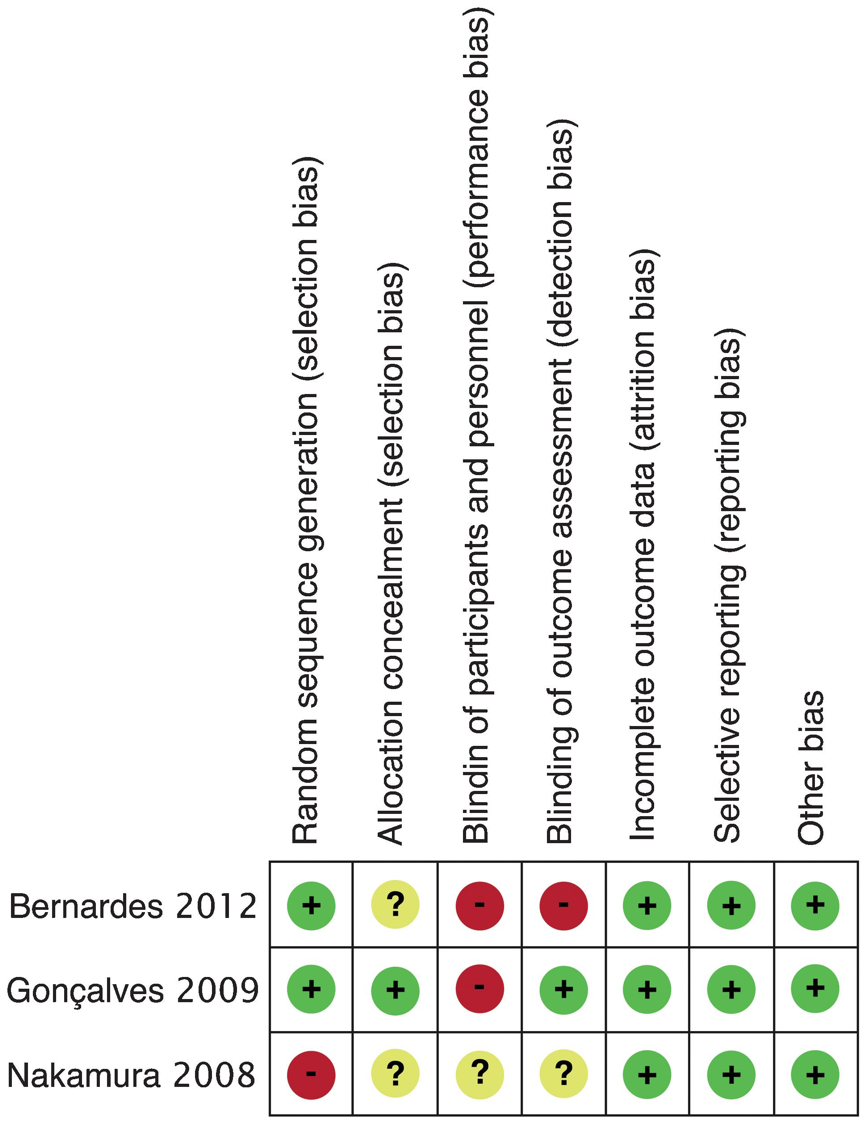 | 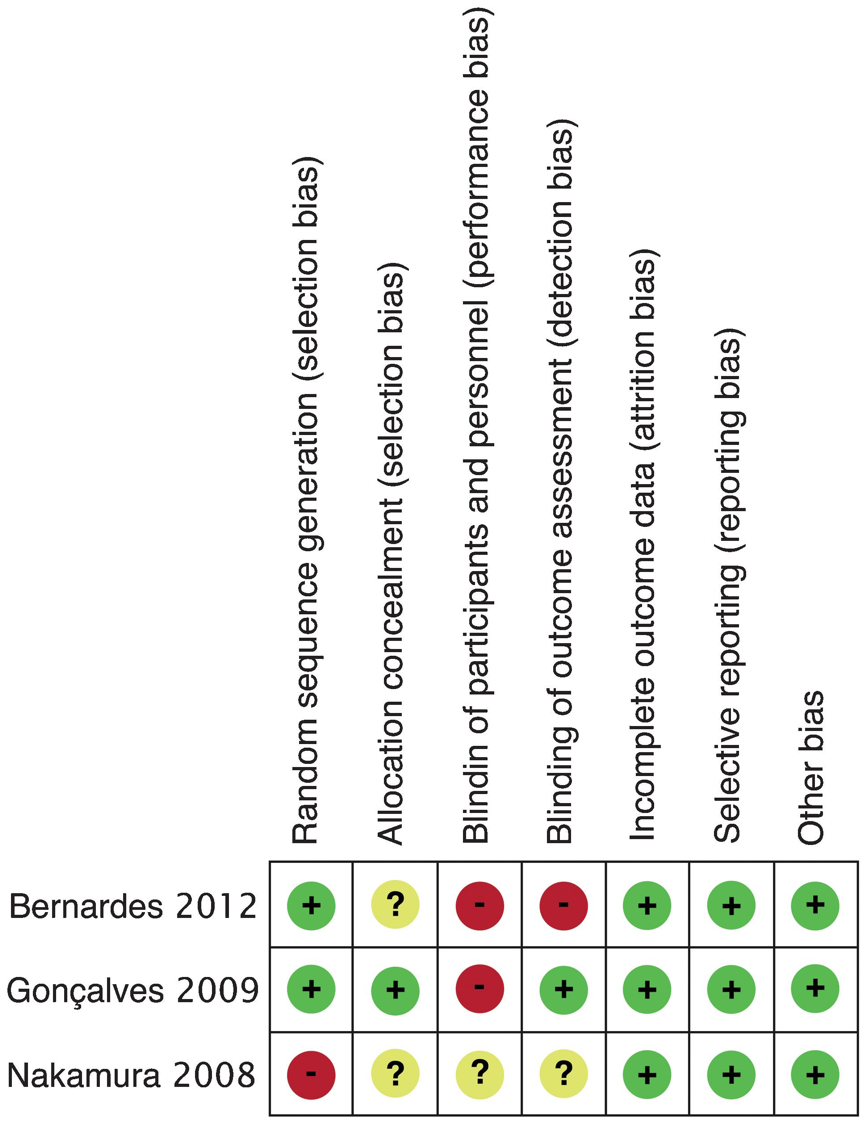 | 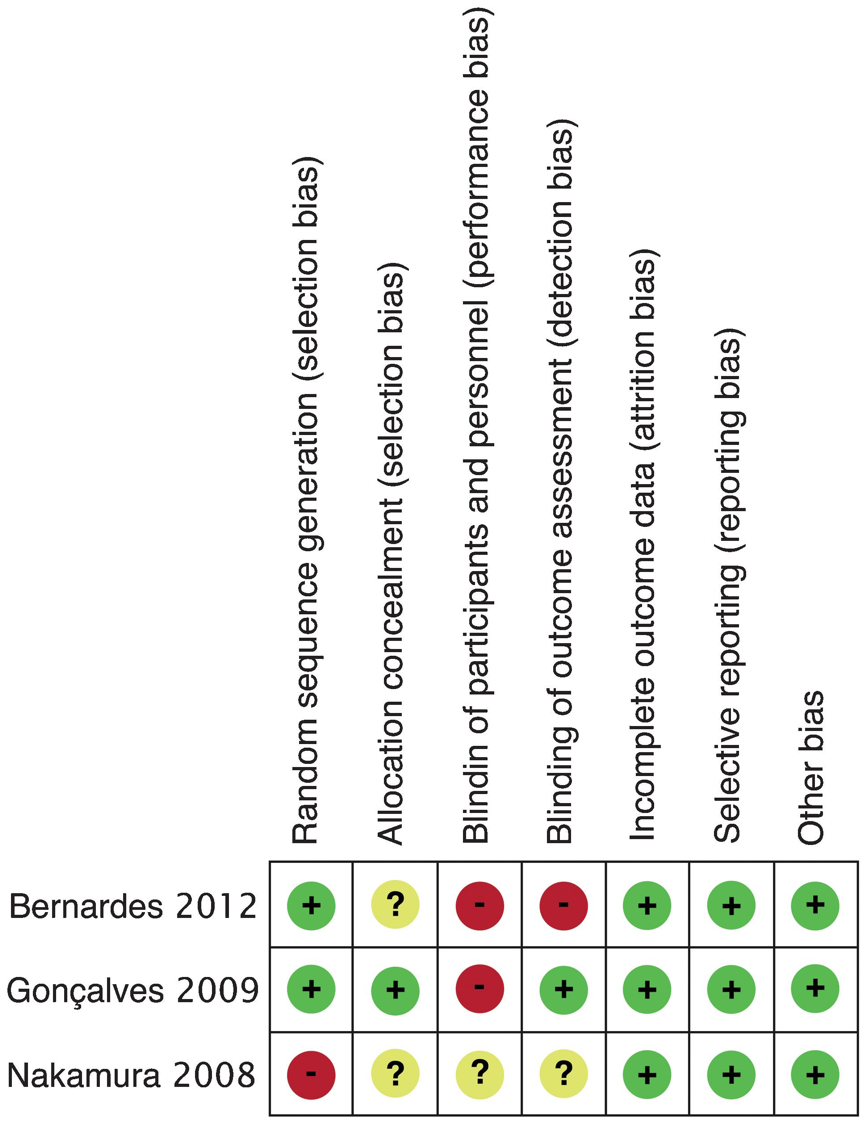 | 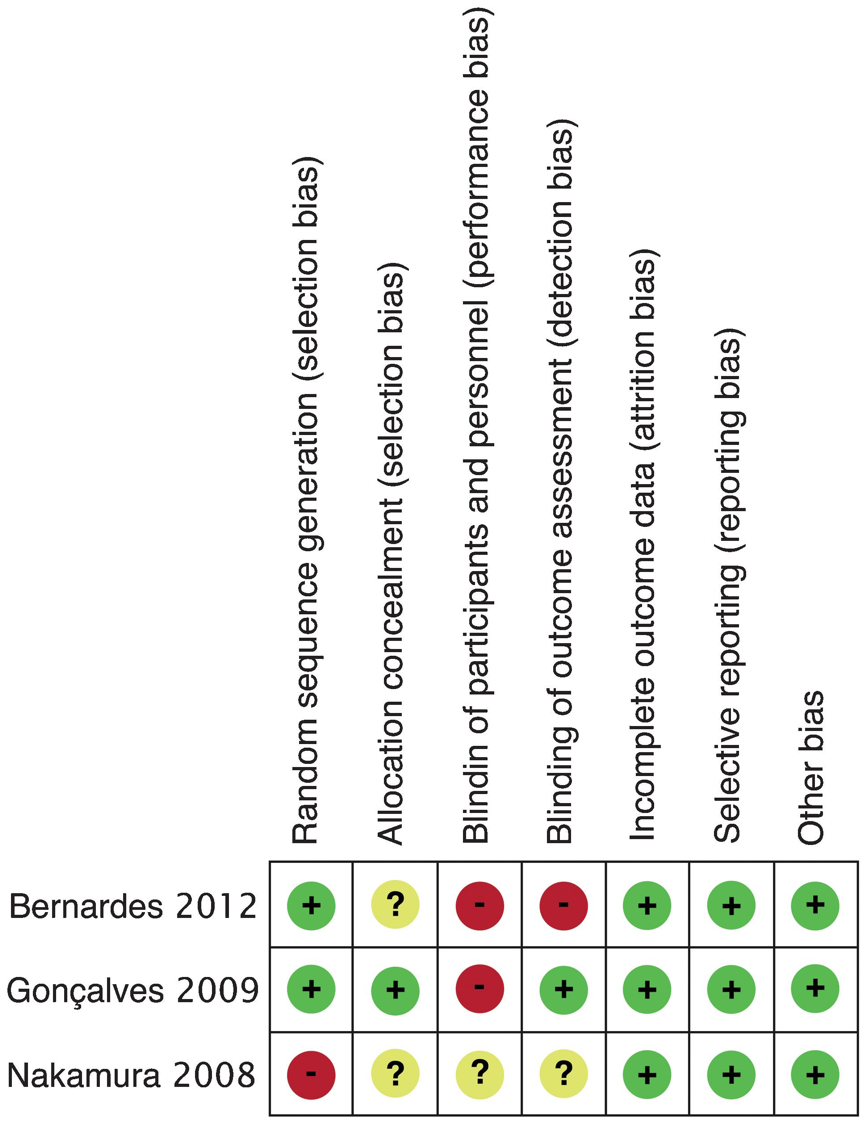 | 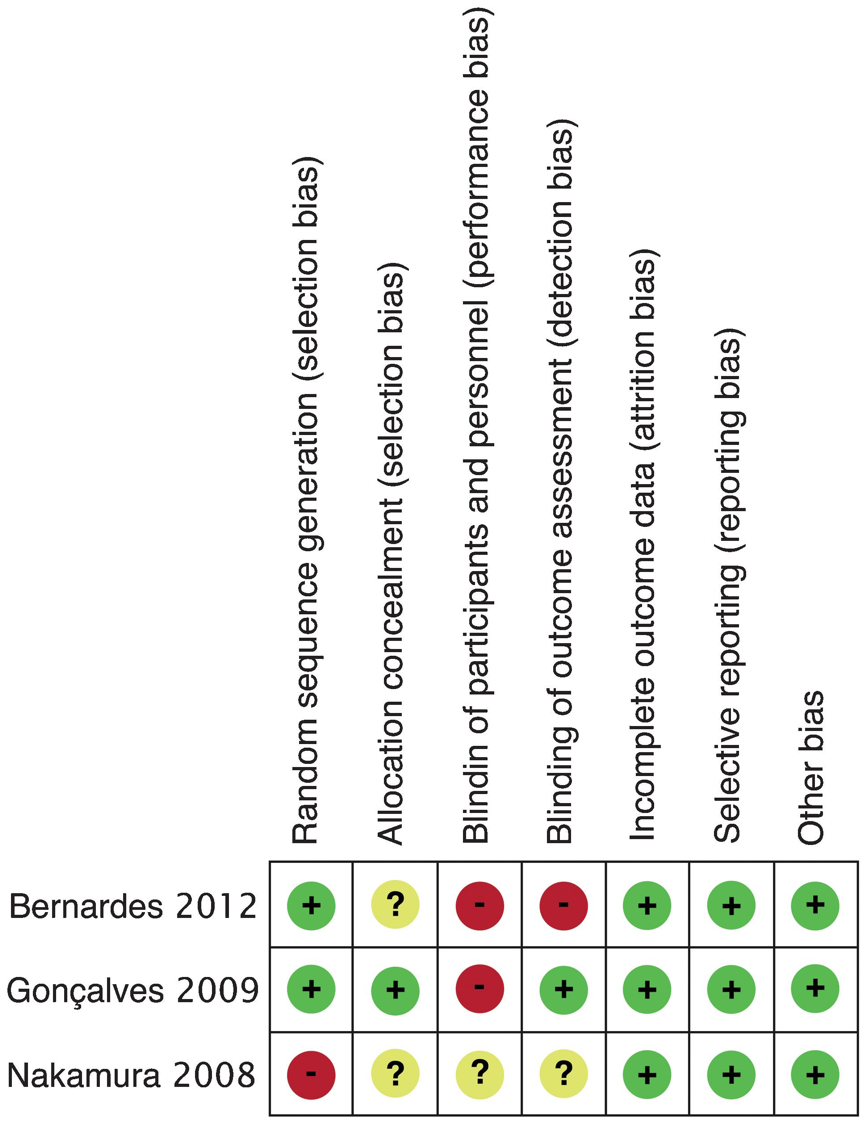 | 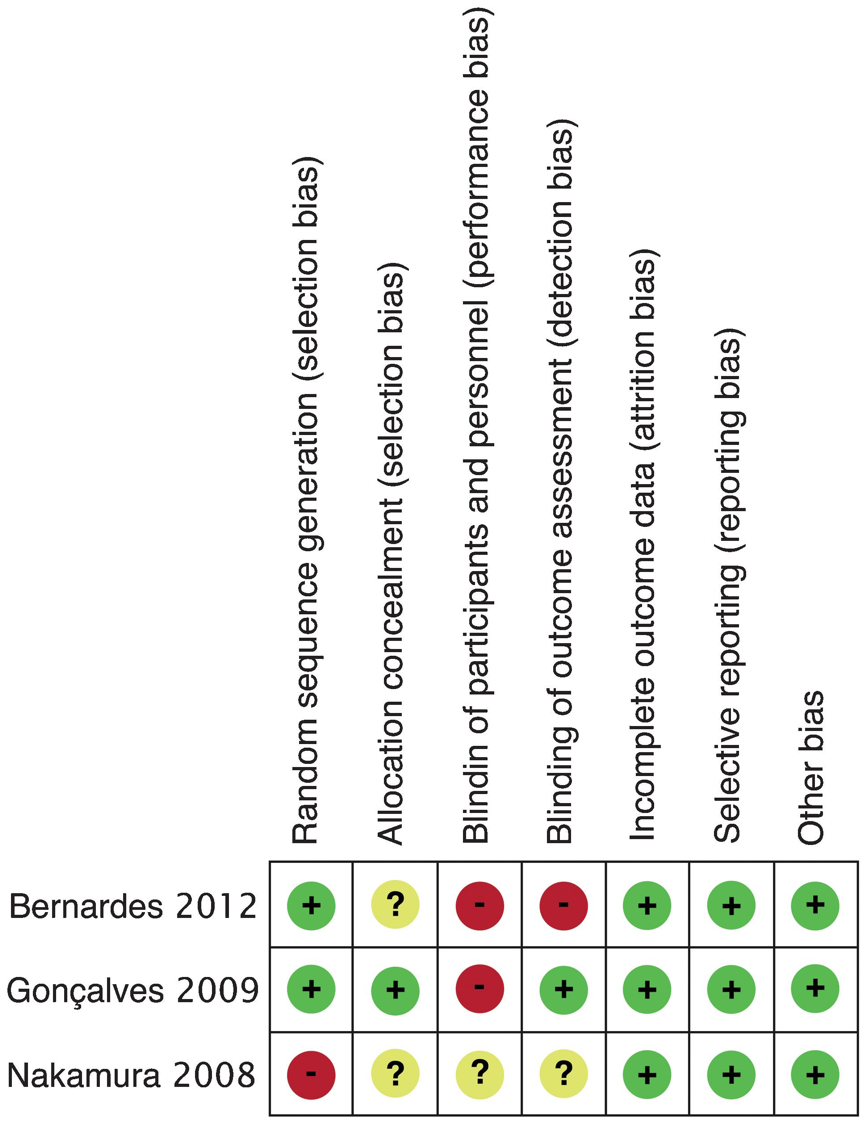 |
| Jalali et al. (2018) ^(29)^ | 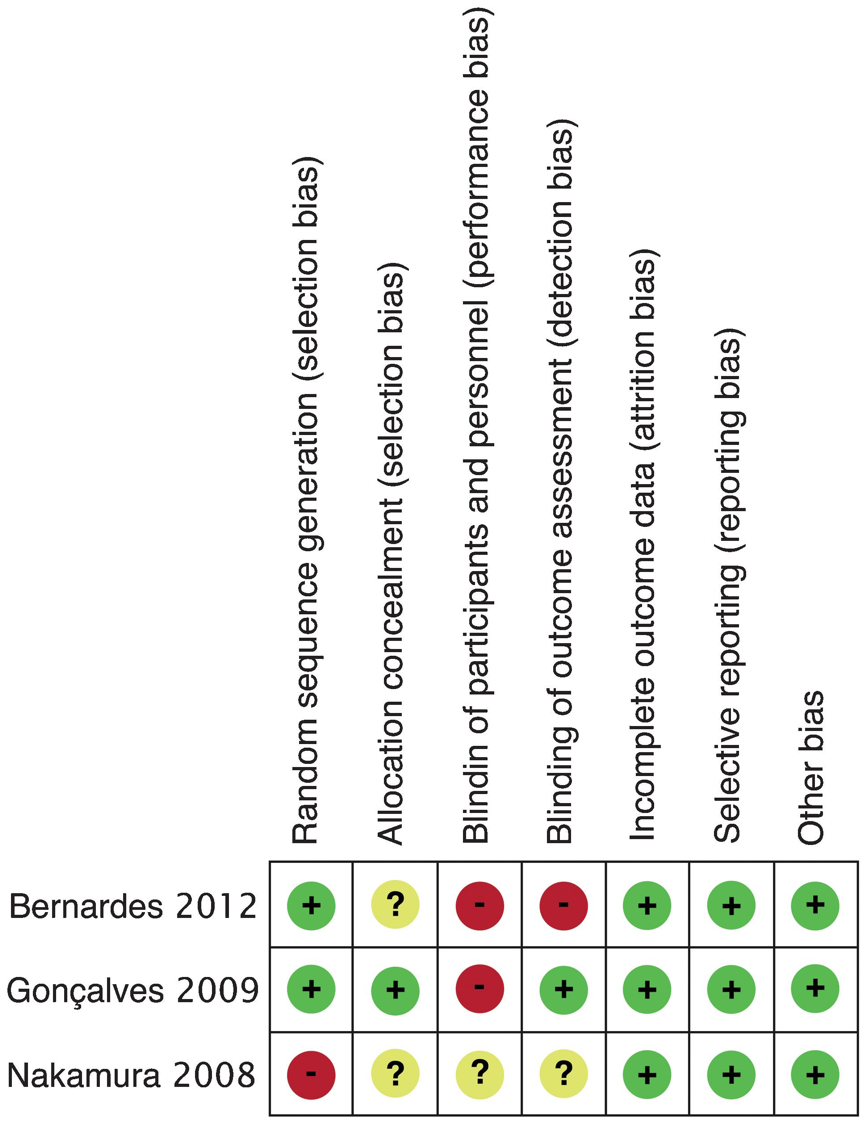 | 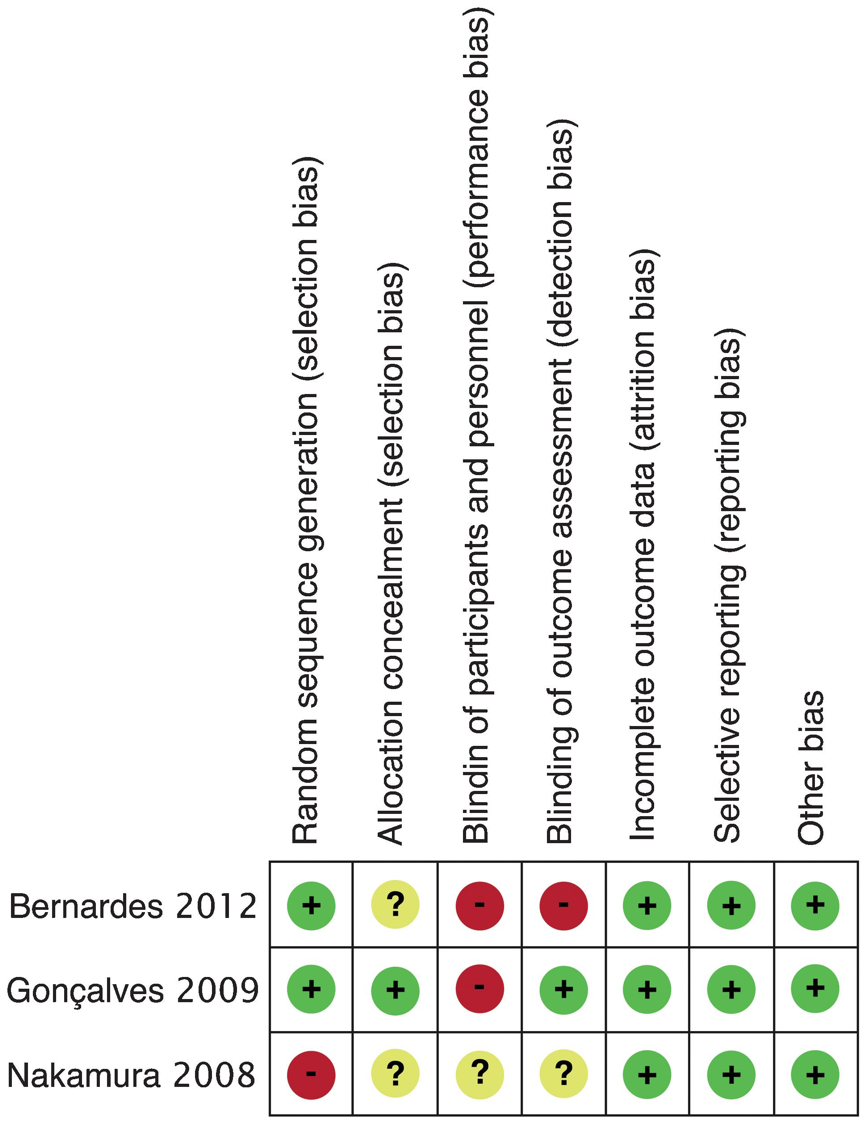 | 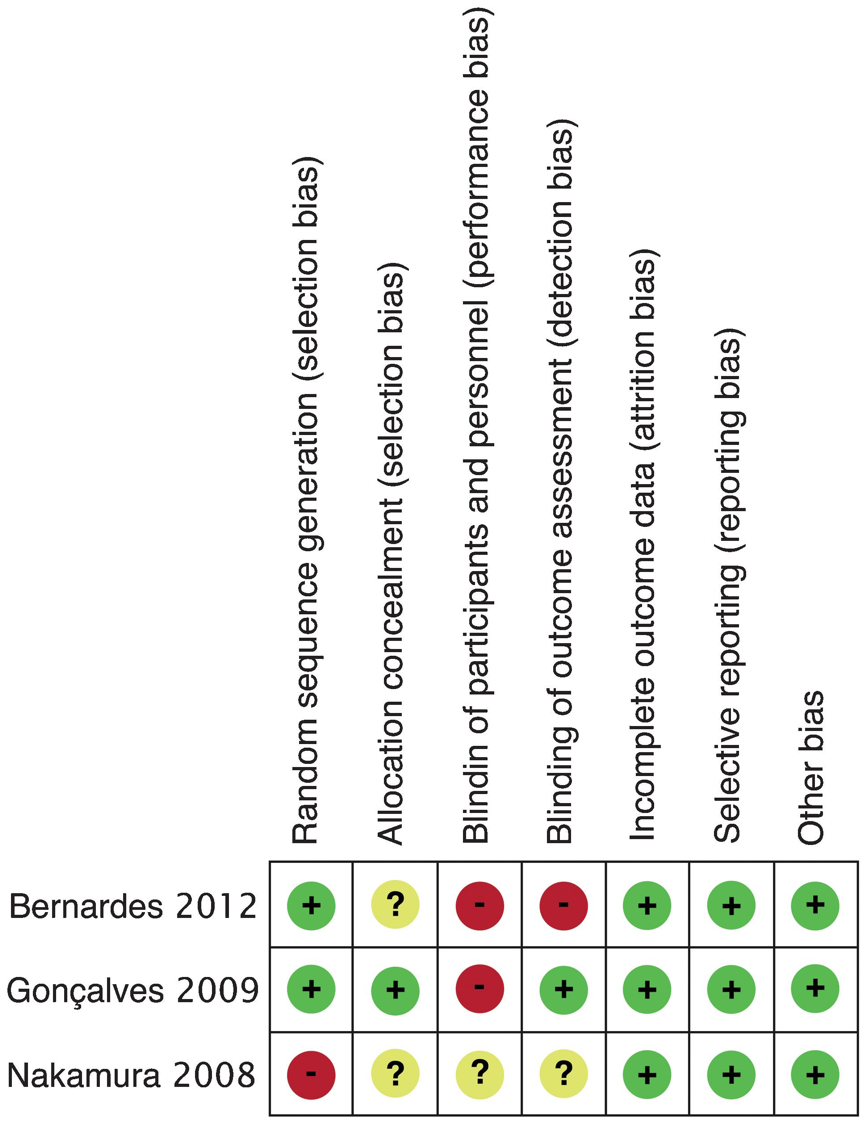 | 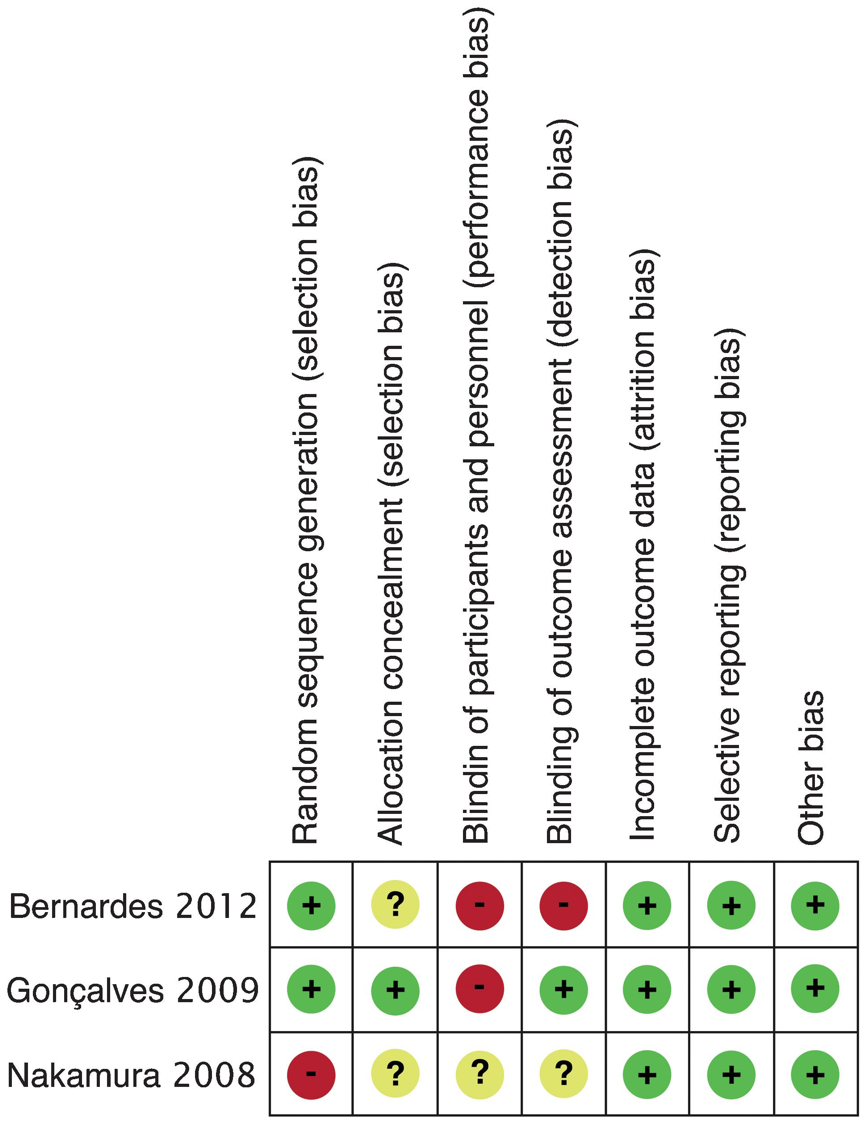 | 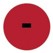 | 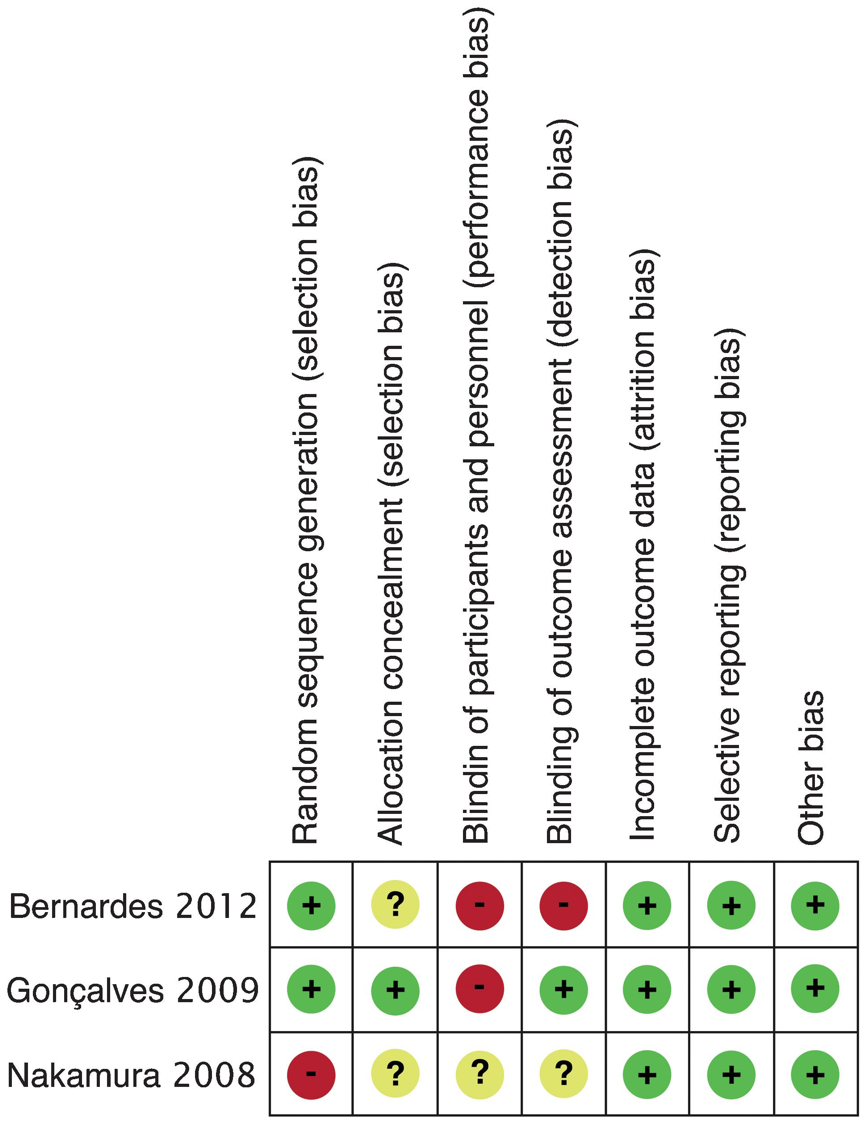 | 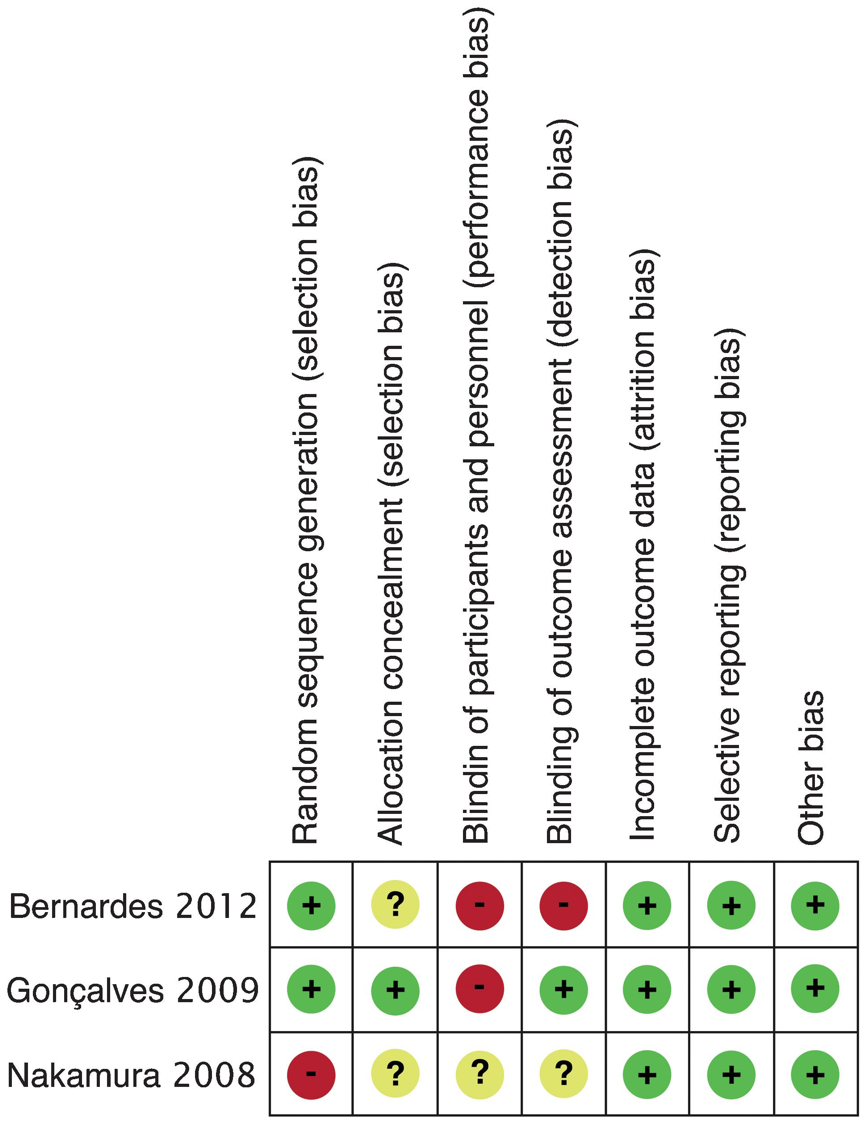 |
| Kleckner et al. (2022) ^(30)^ | 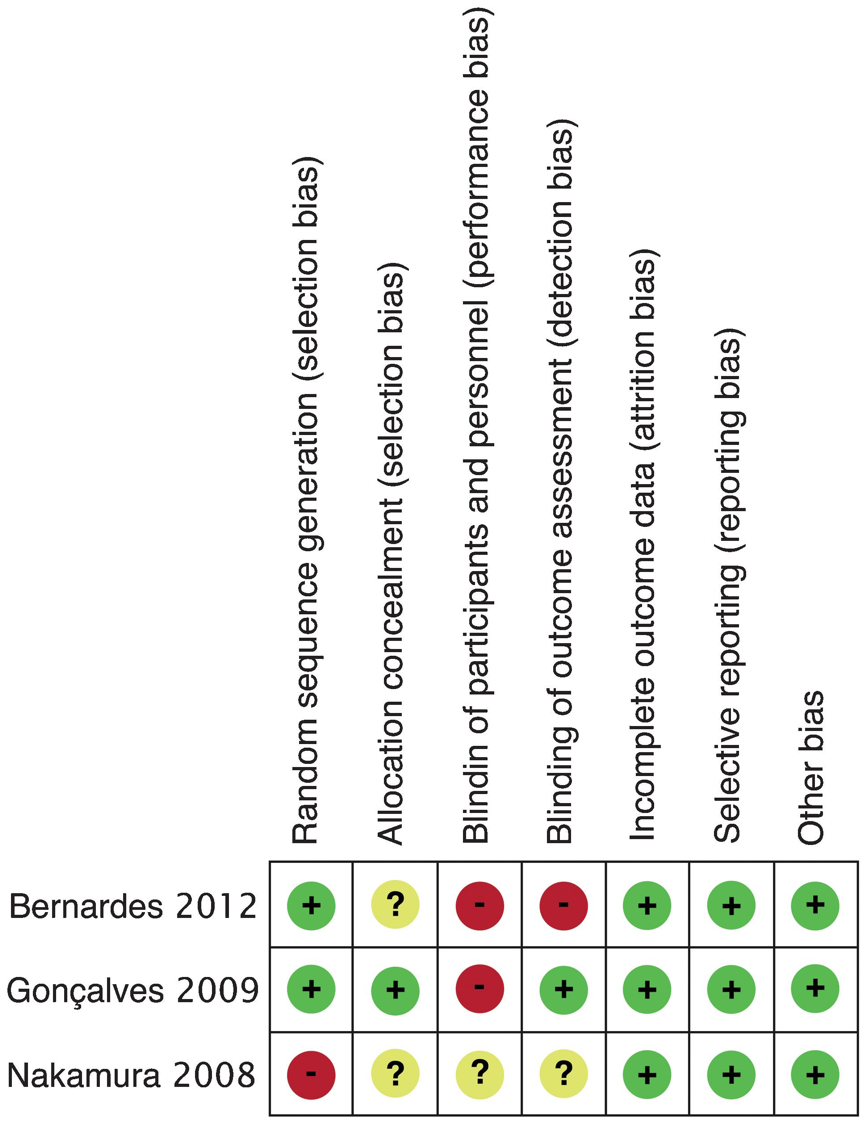 | 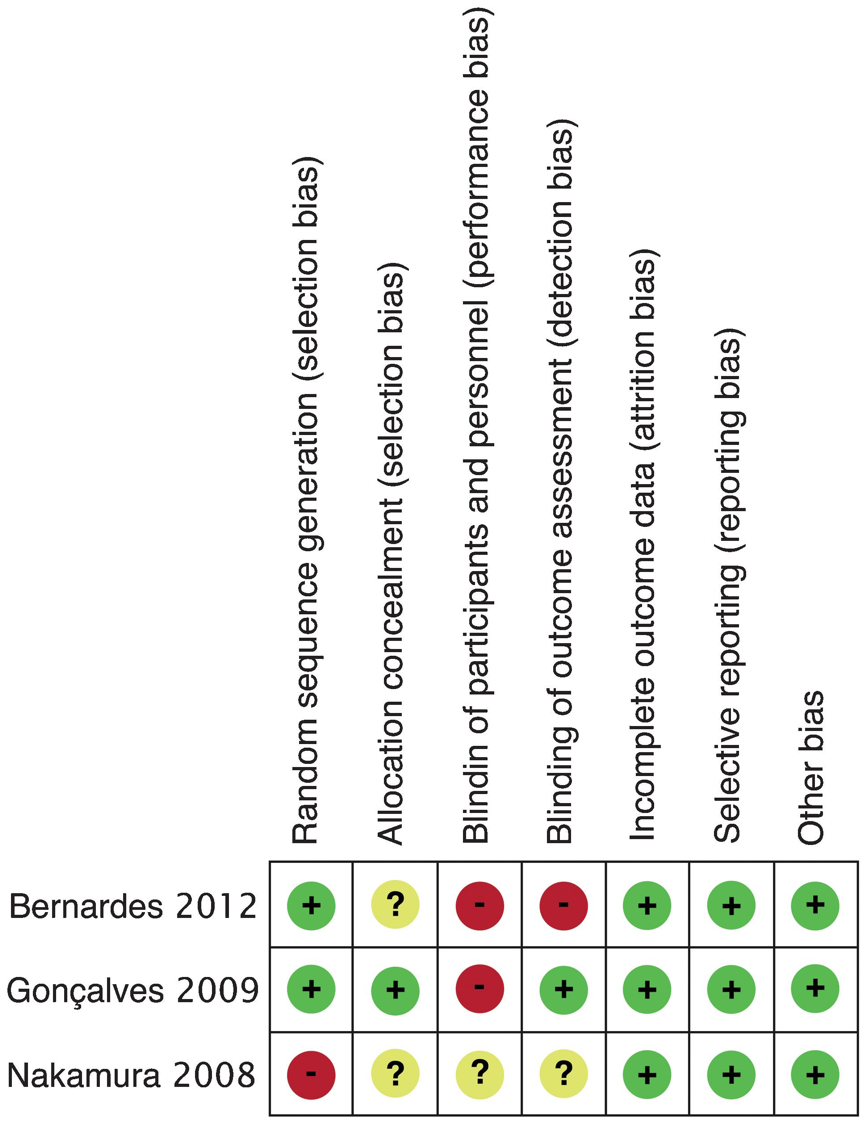 | 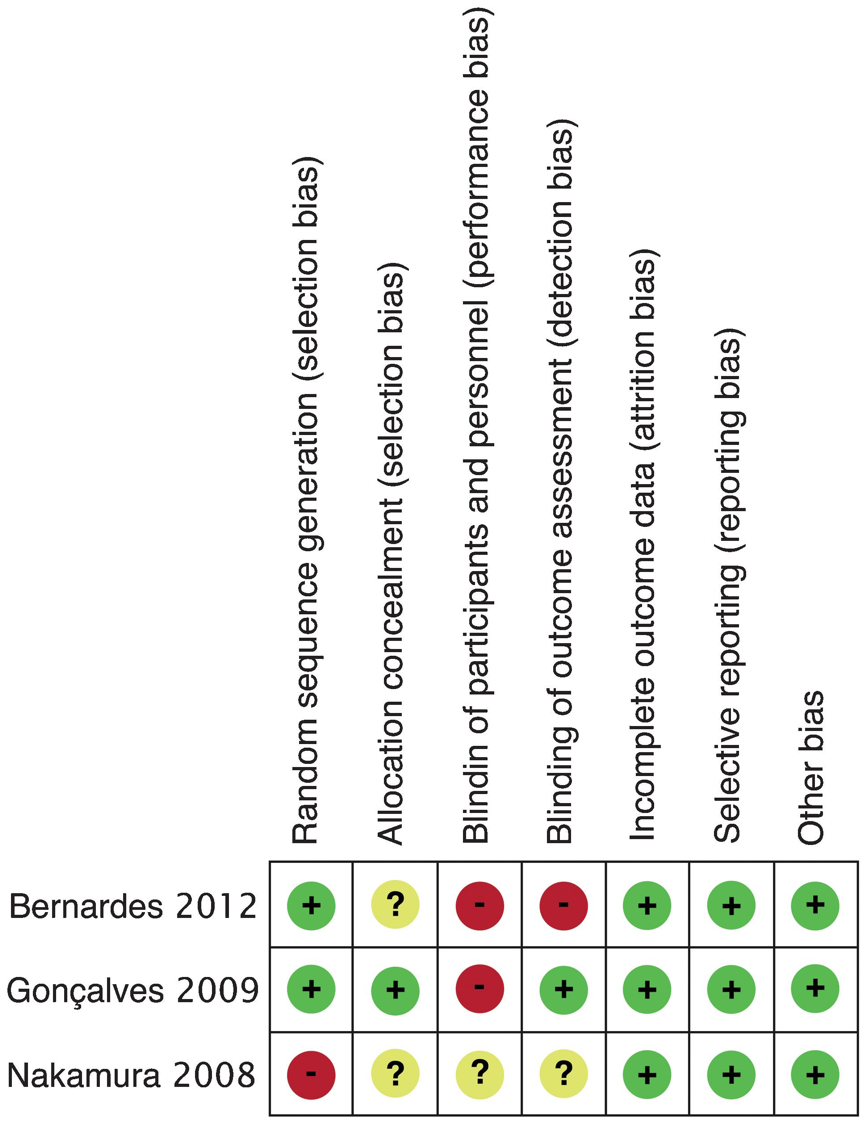 | 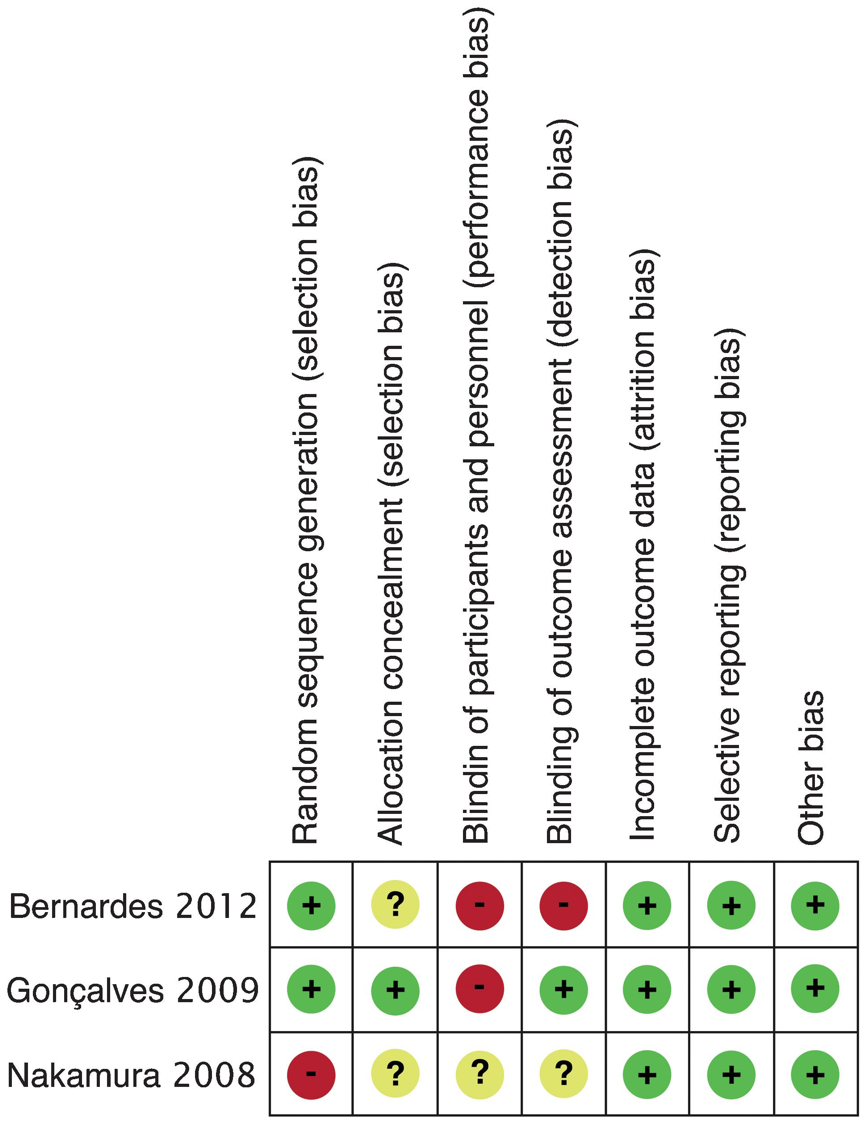 | 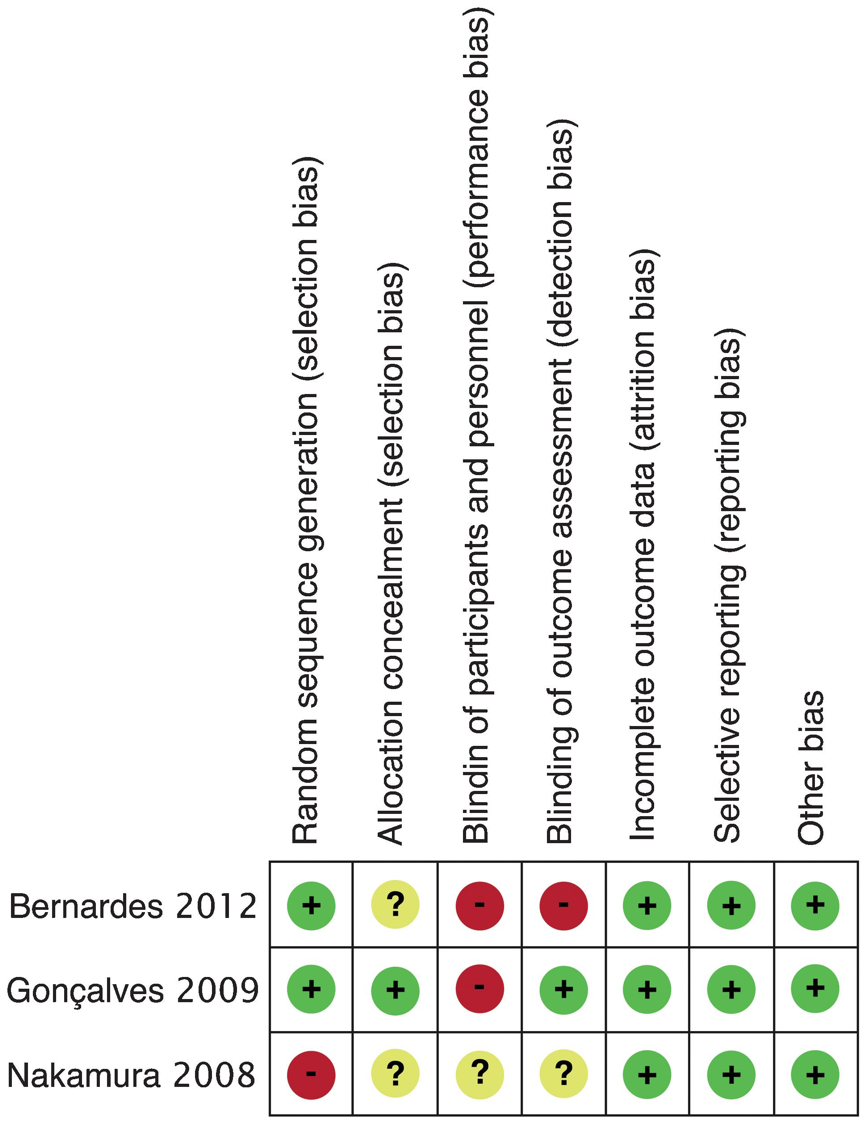 | 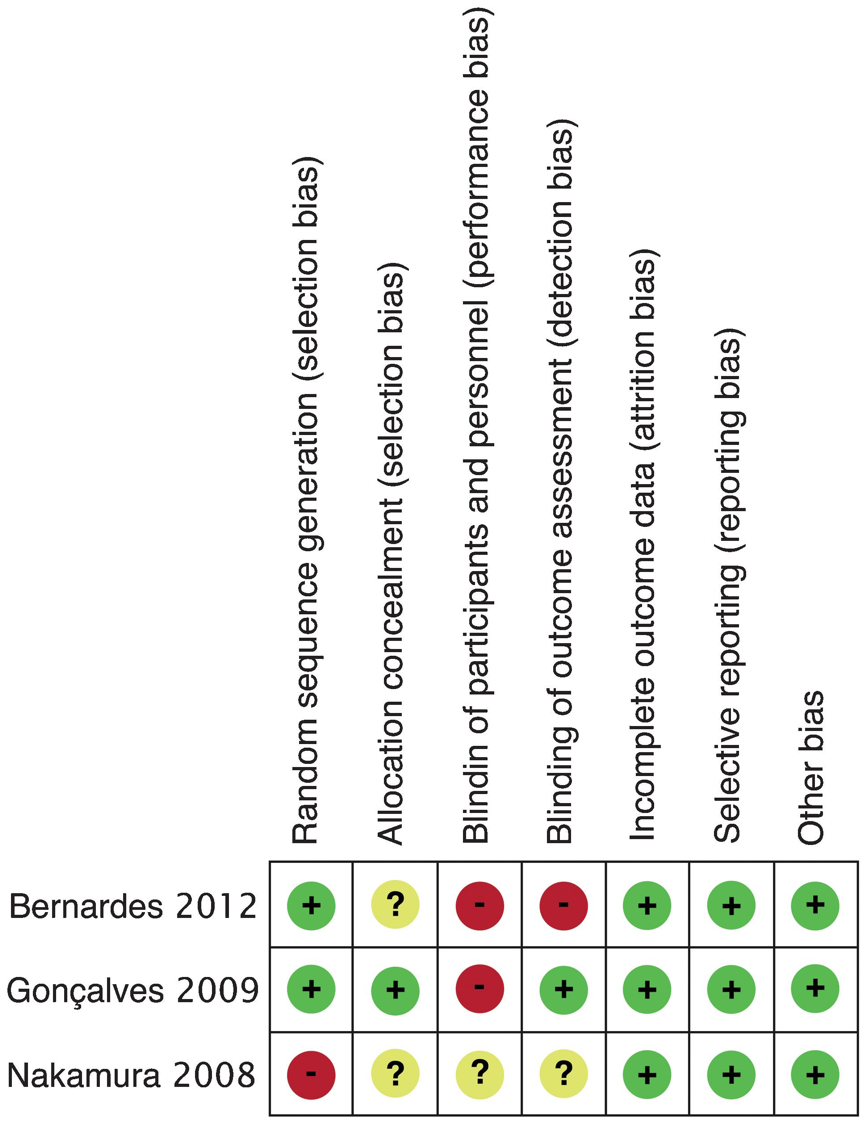 | 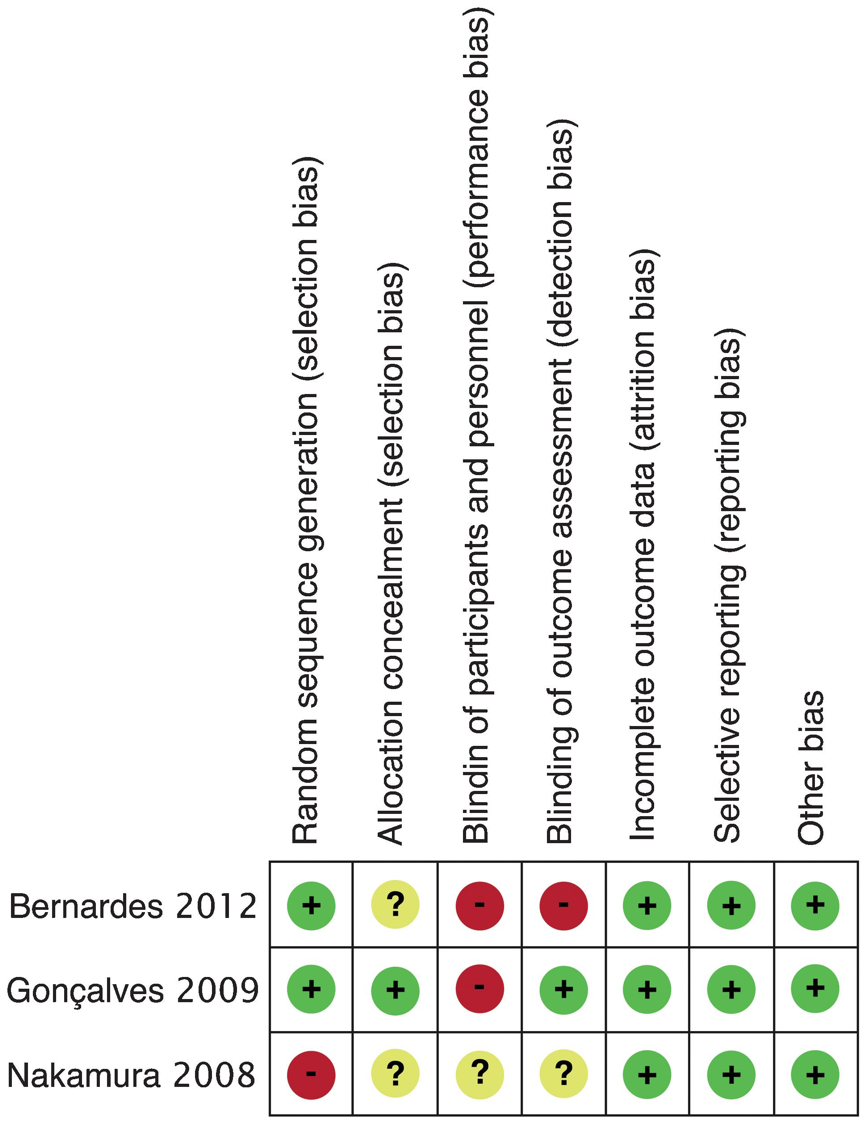 |
| Long Parma et al. (2022) ^(31)^ | 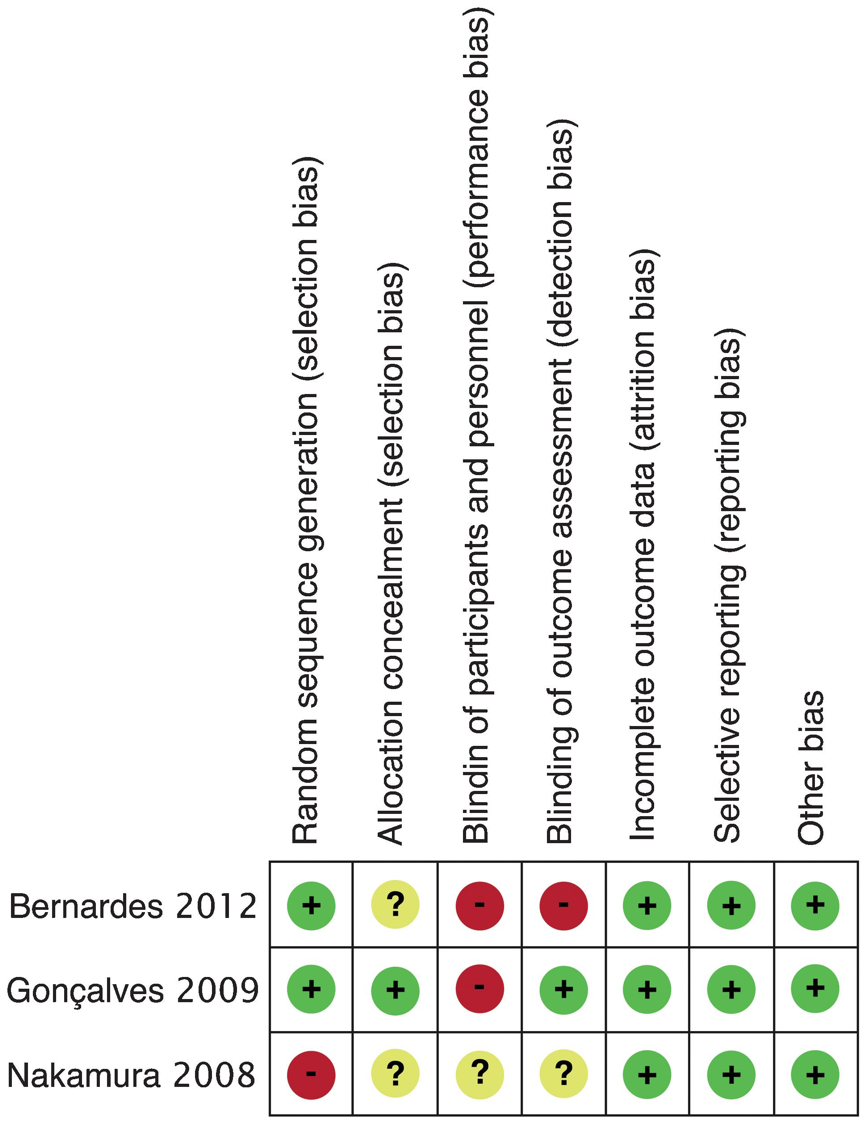 | 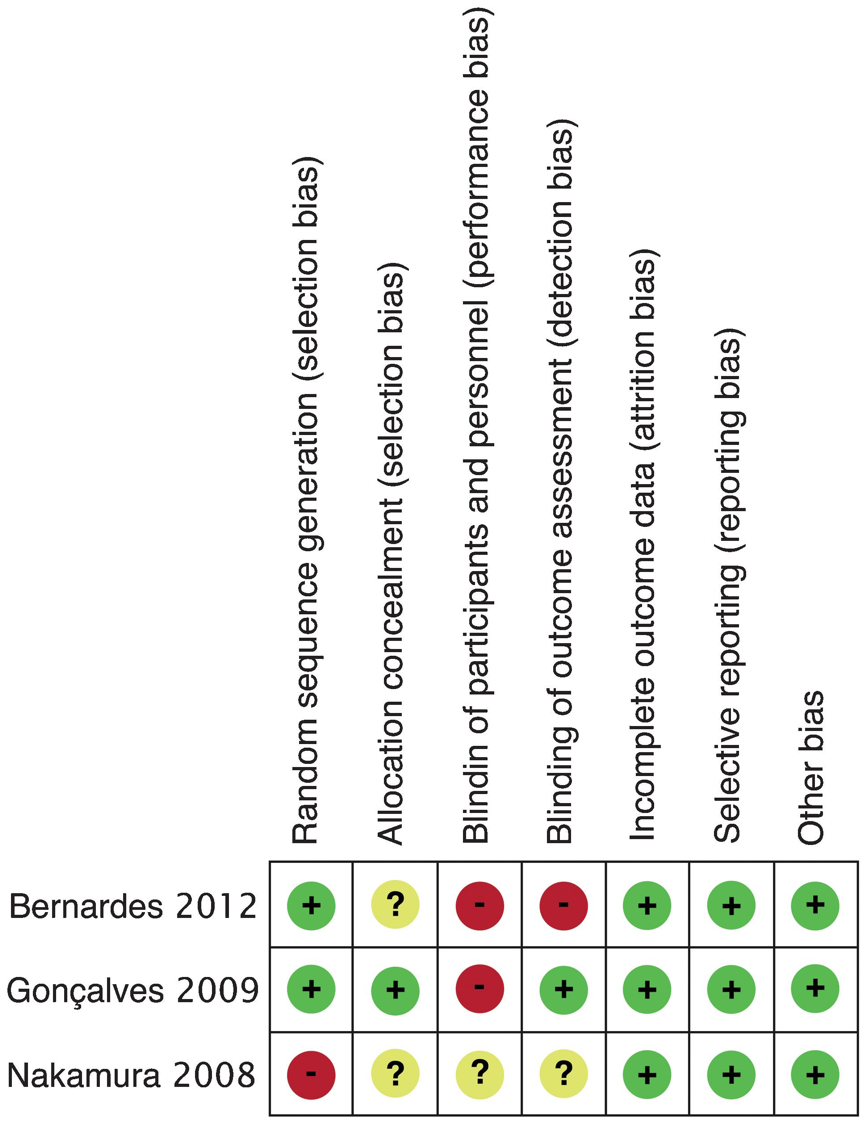 | 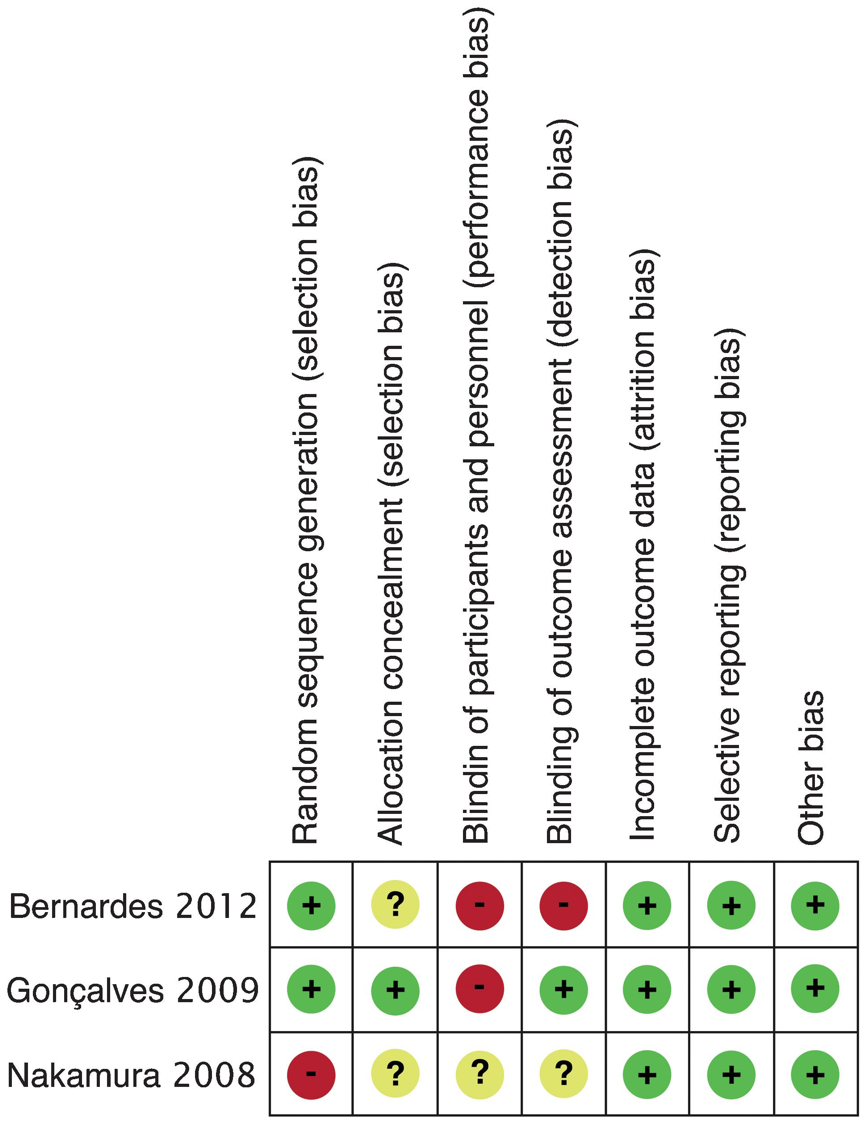 | 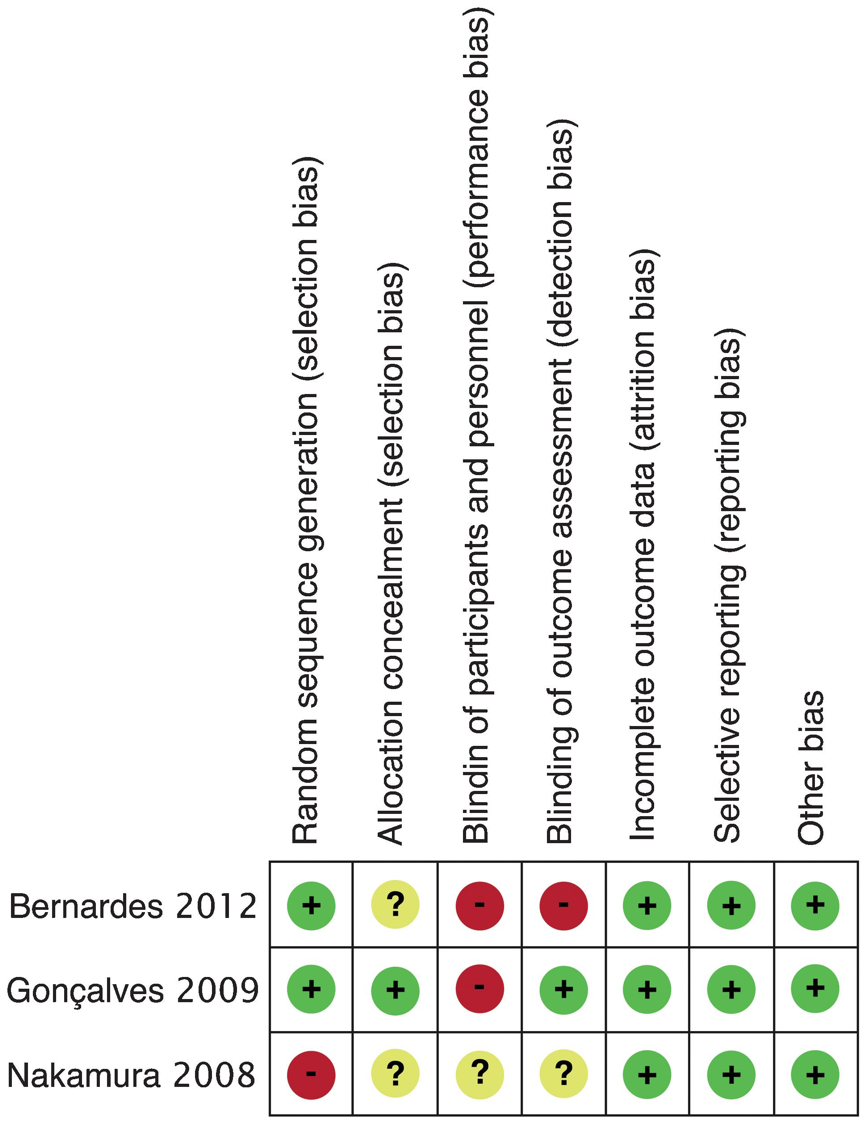 | 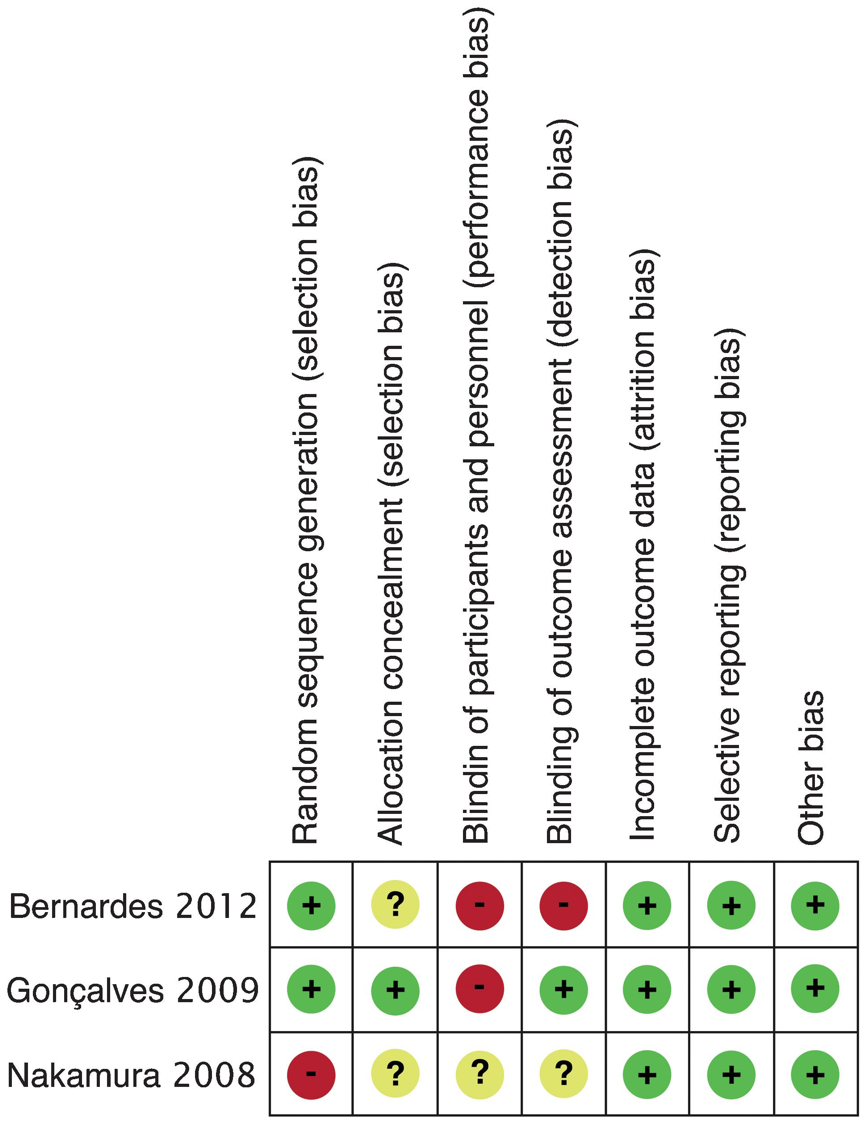 | 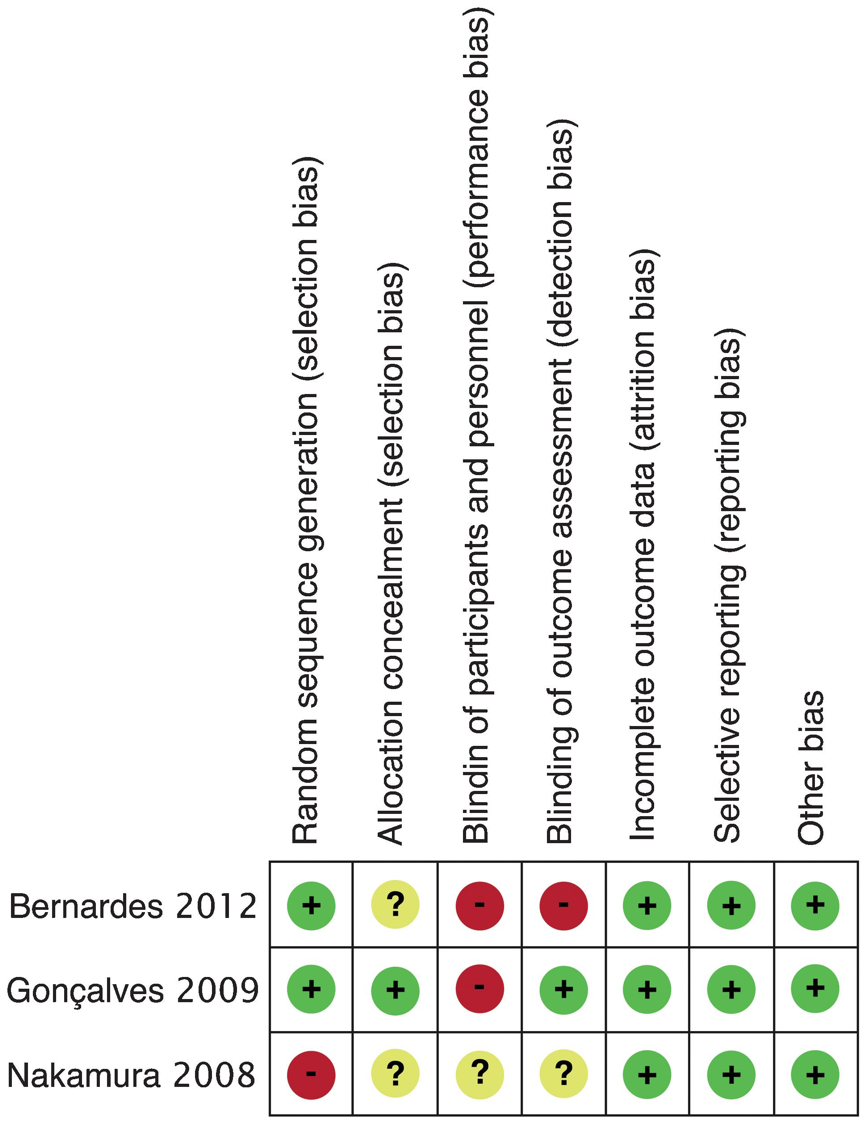 | 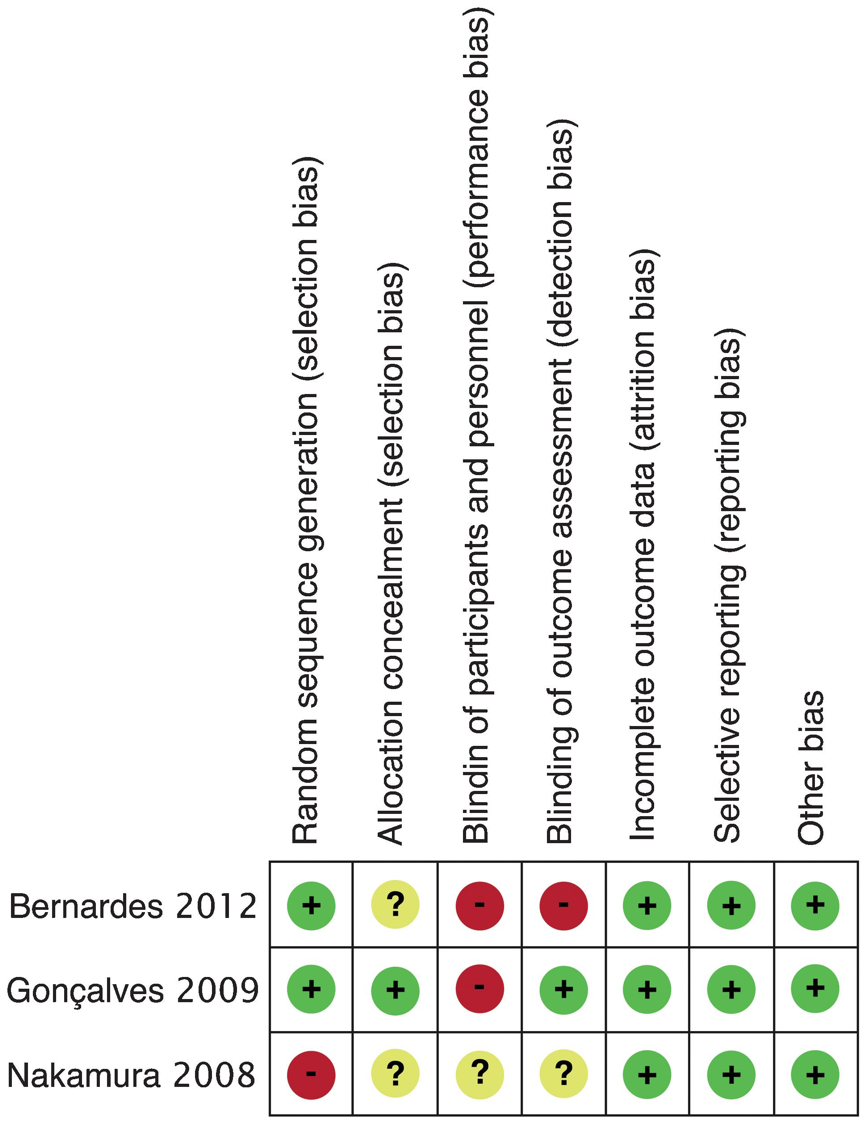 |
| Papandreou et al. (2021) ^(32)^ | 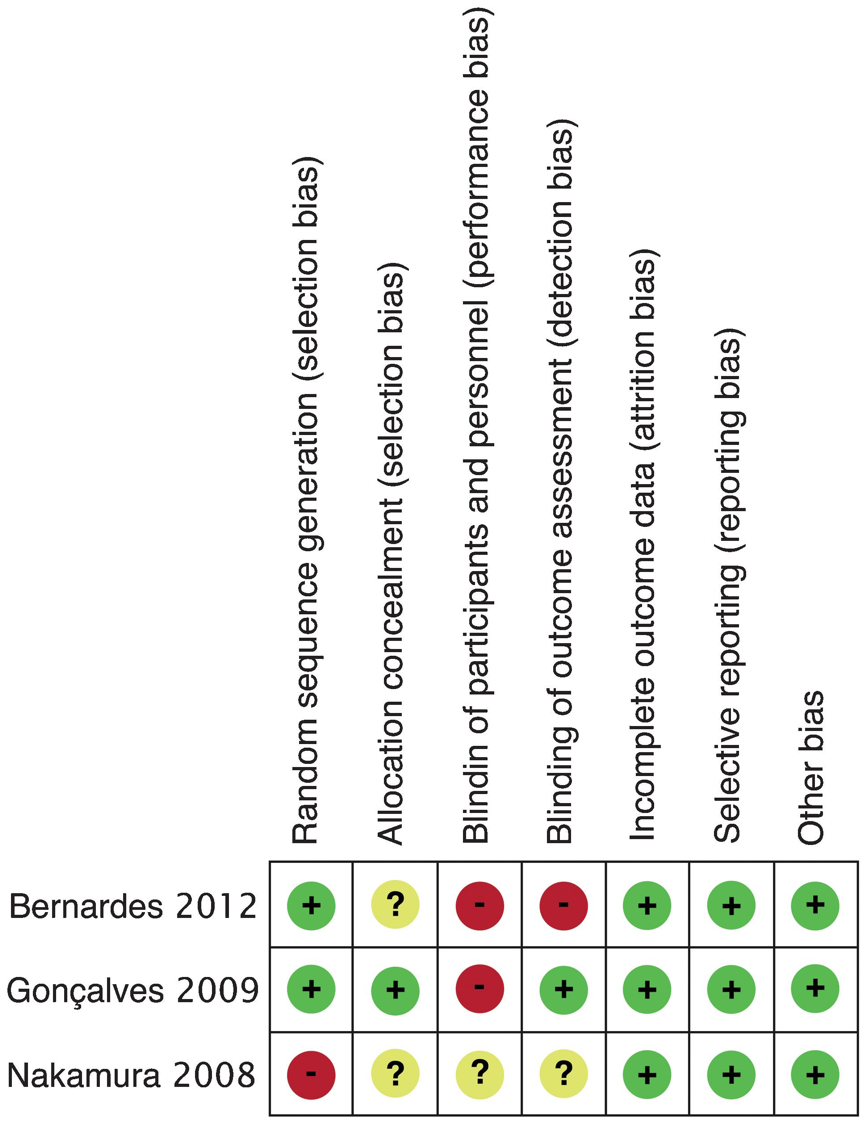 | 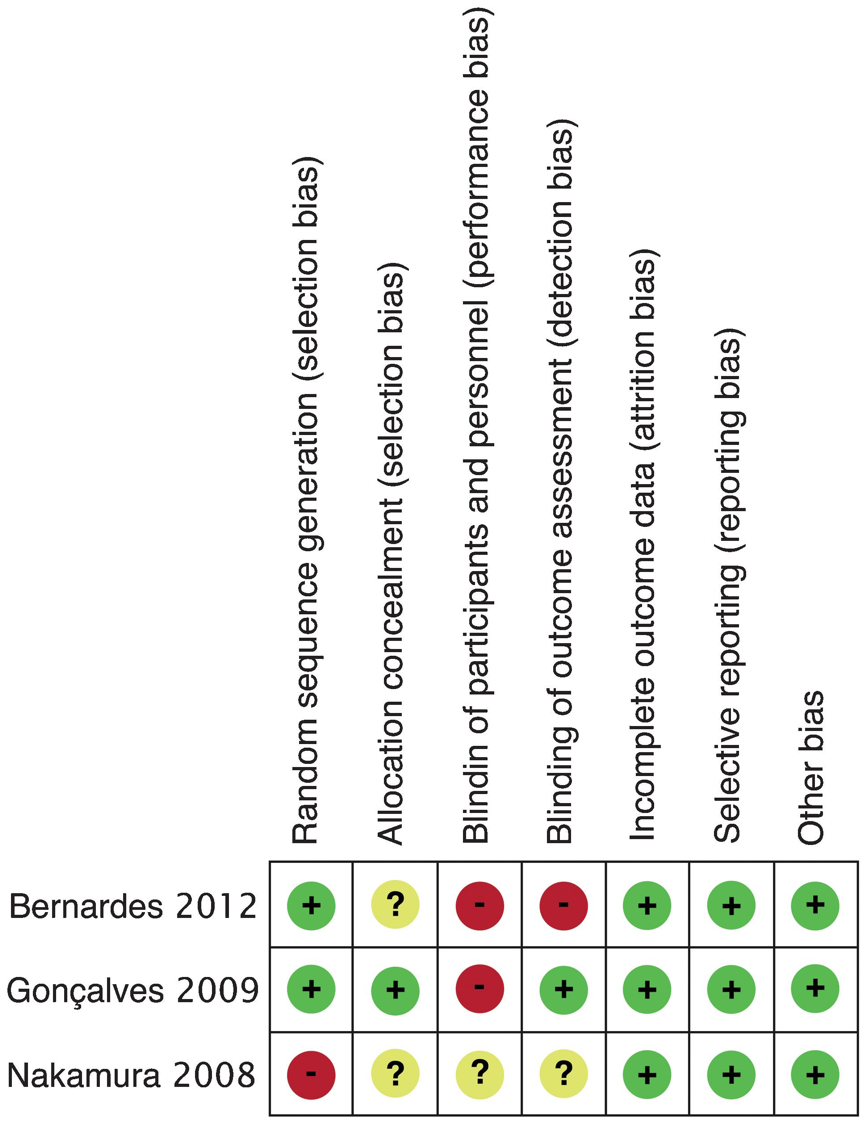 | 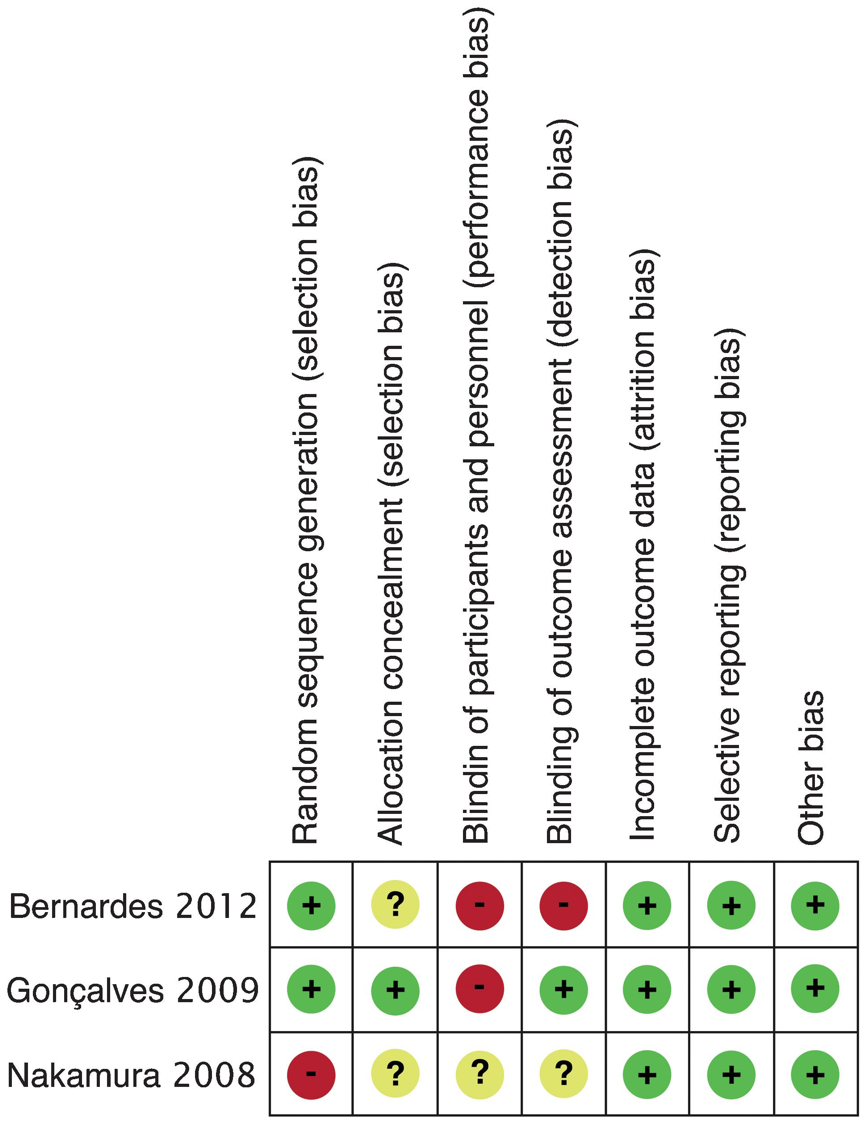 | 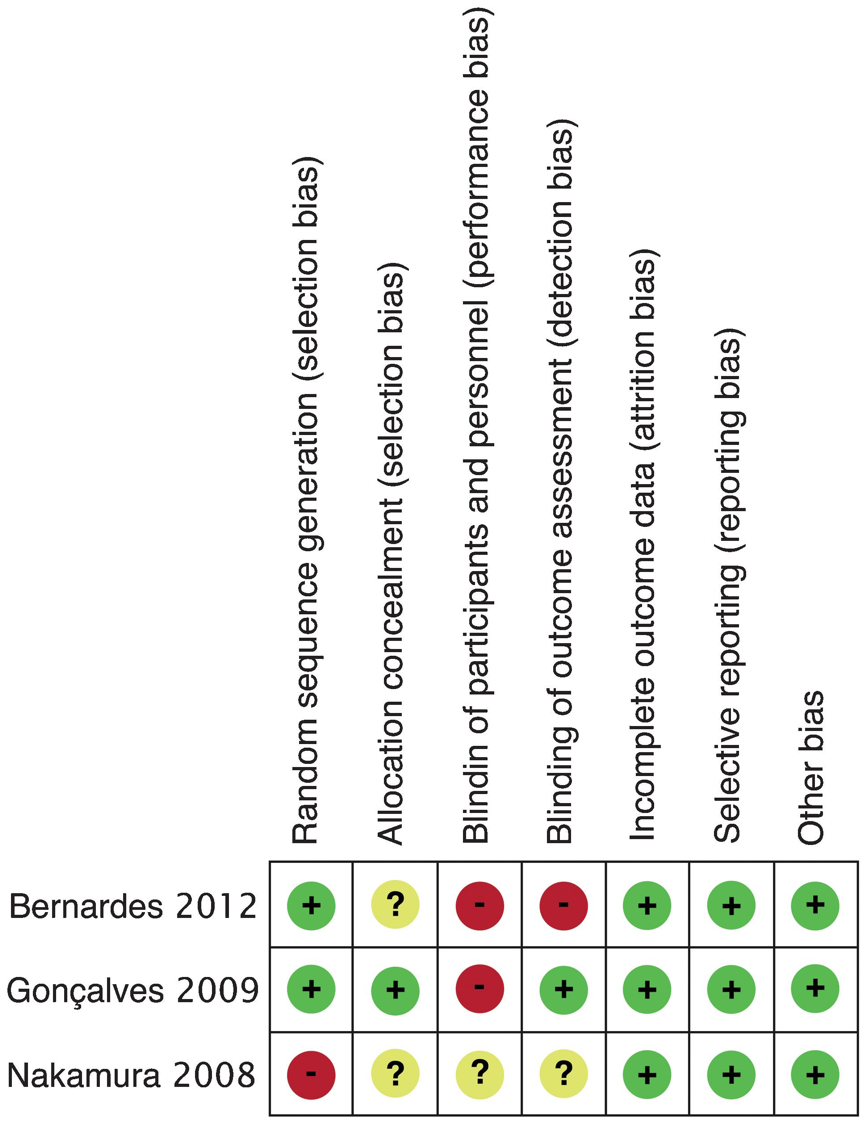 | 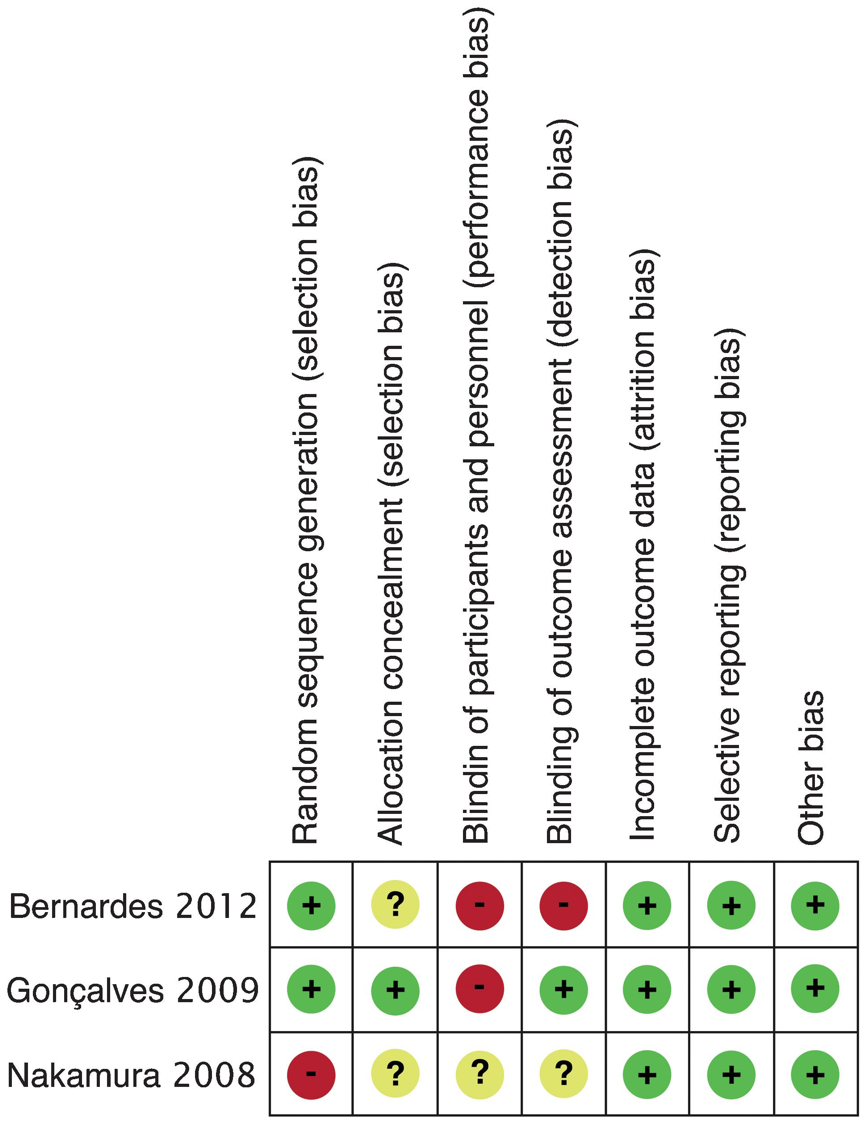 | 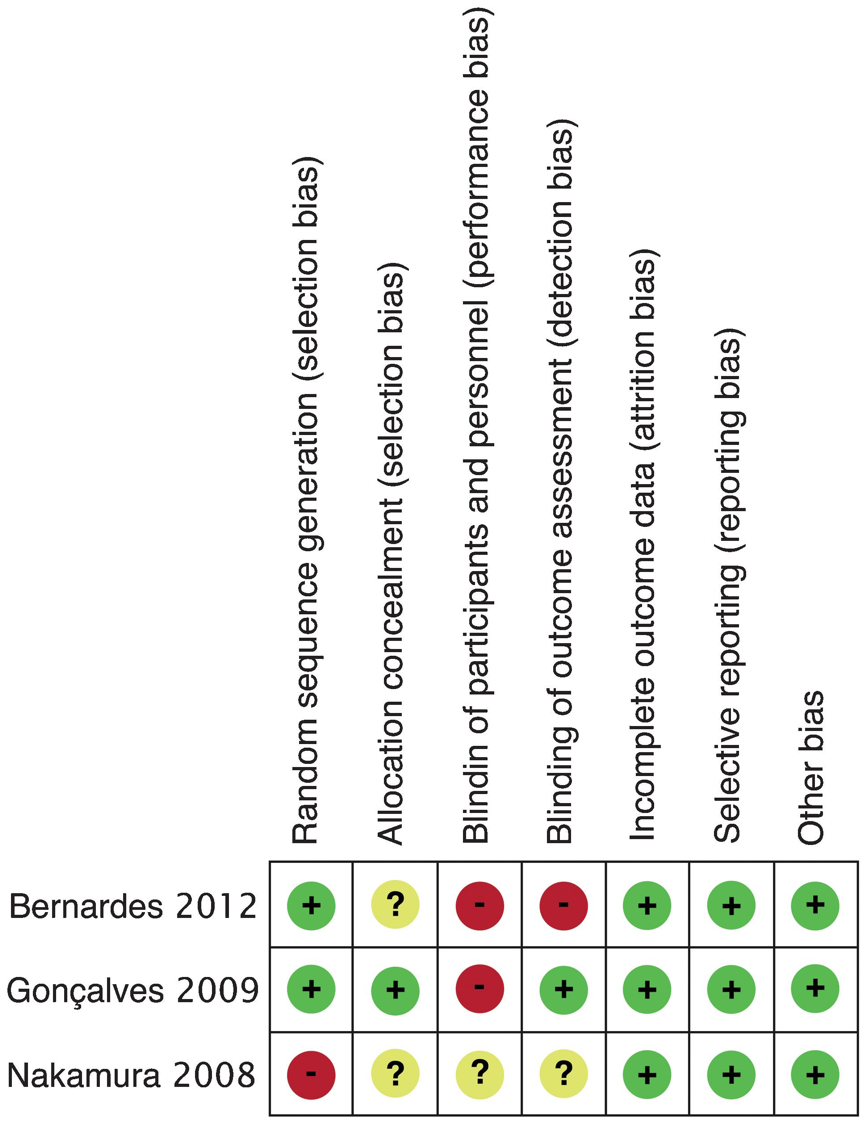 | 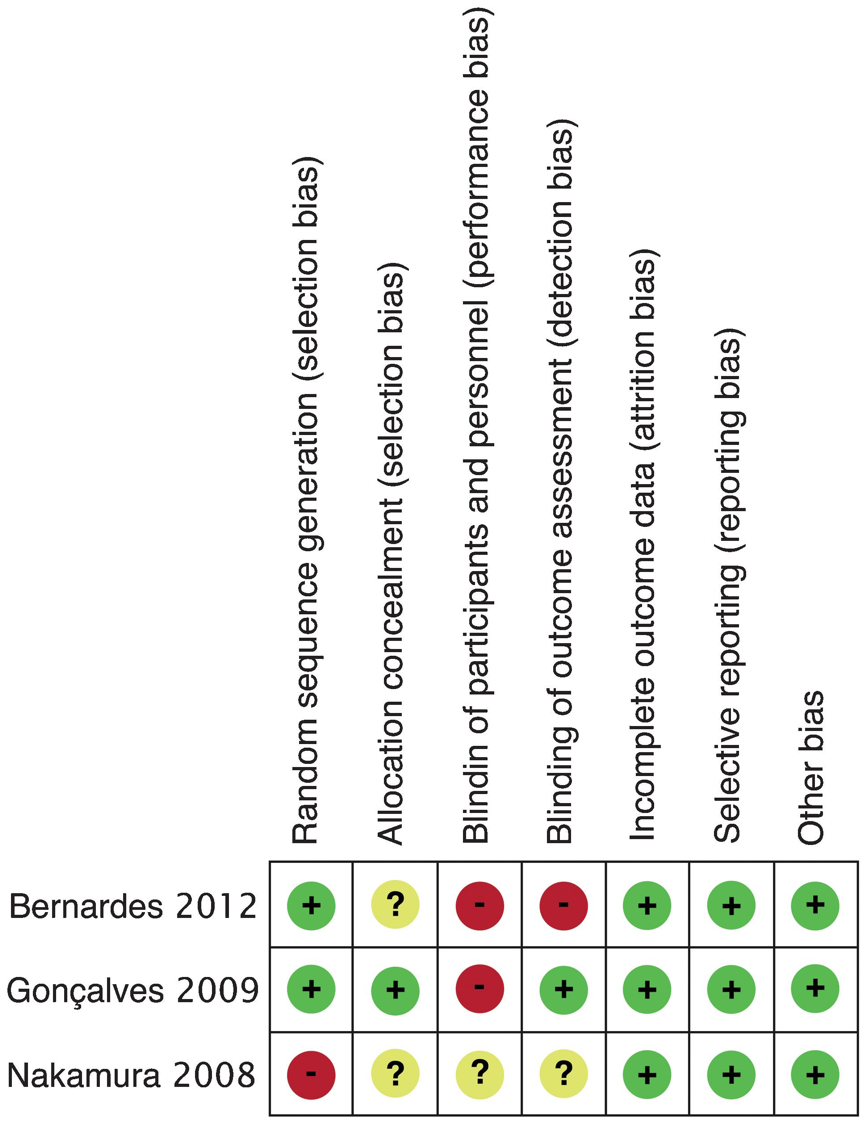 |
| Ruiz-Vozmediano et al. (2020) ^(33)^ | 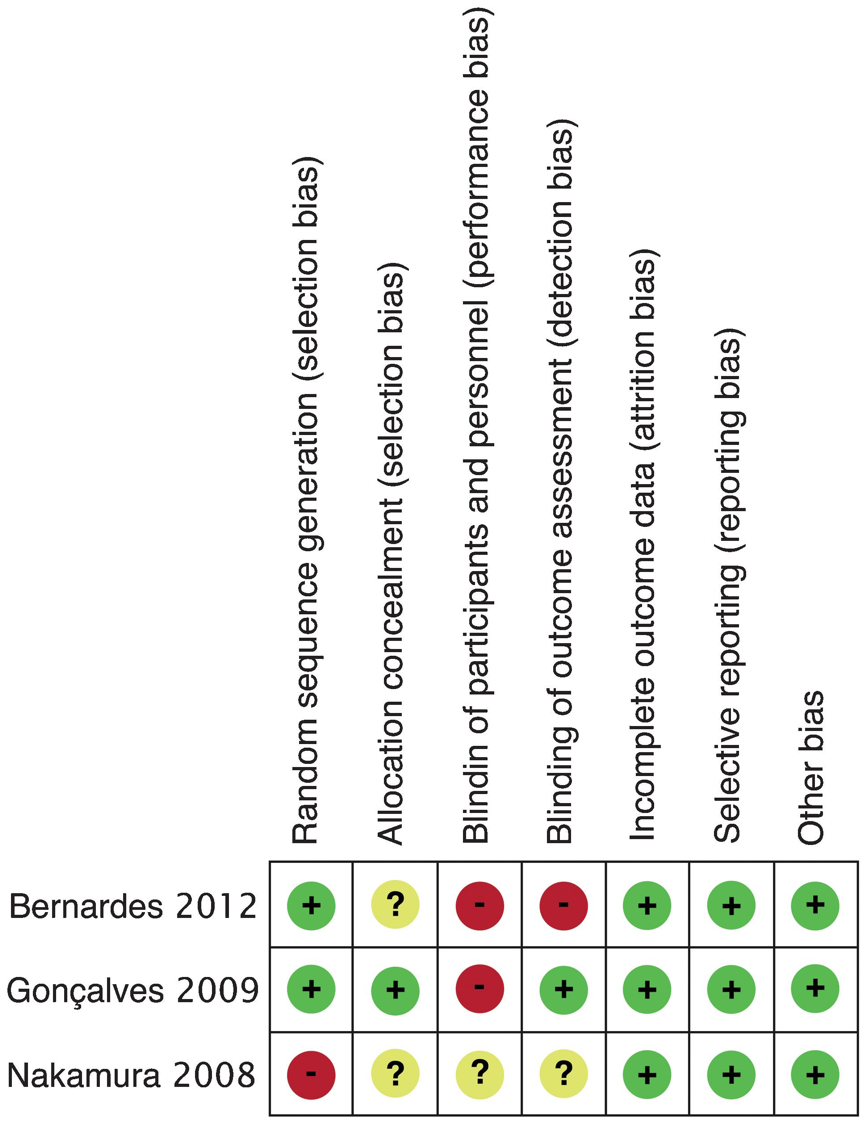 | 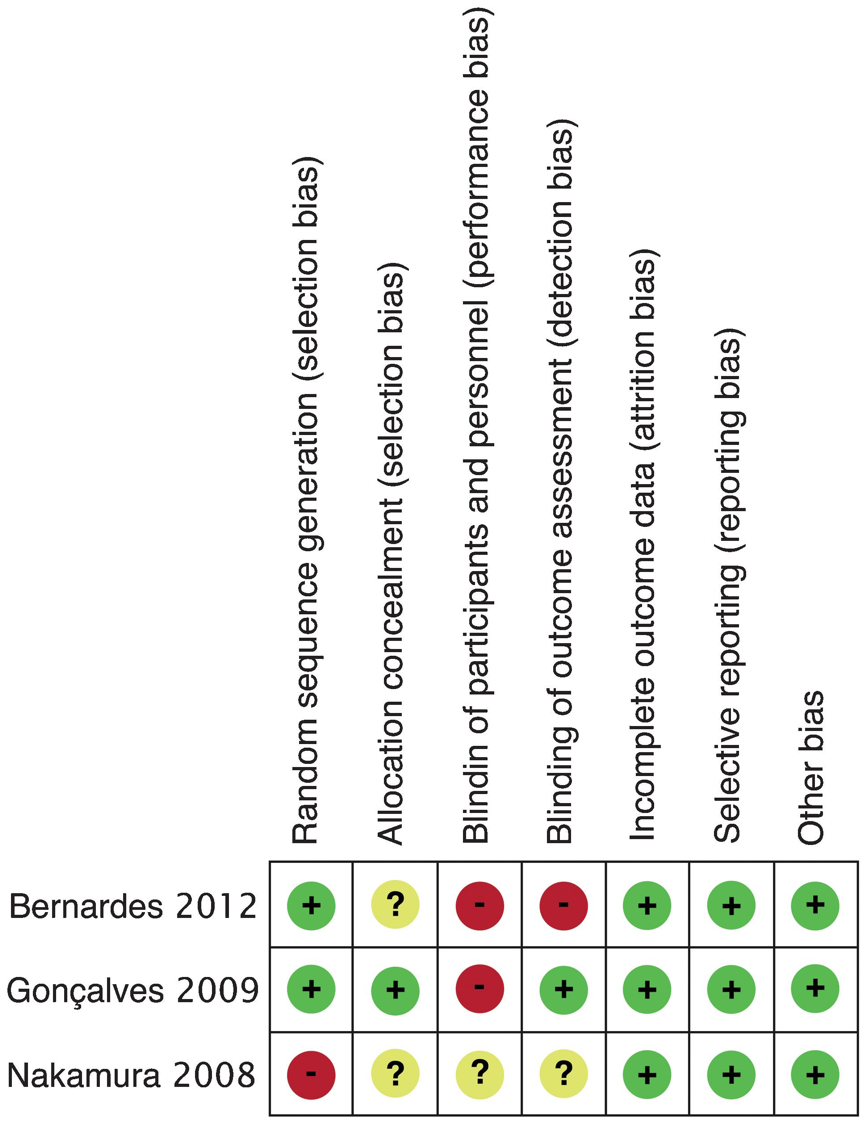 | 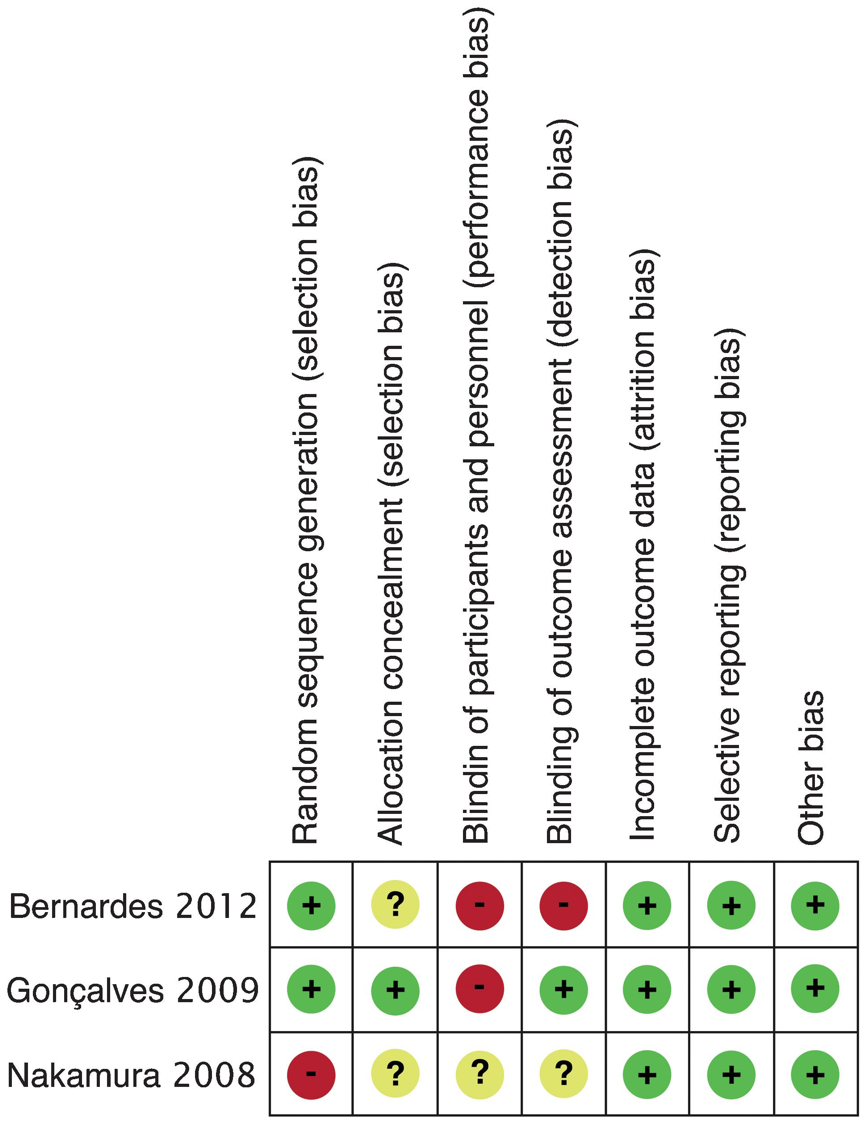 | 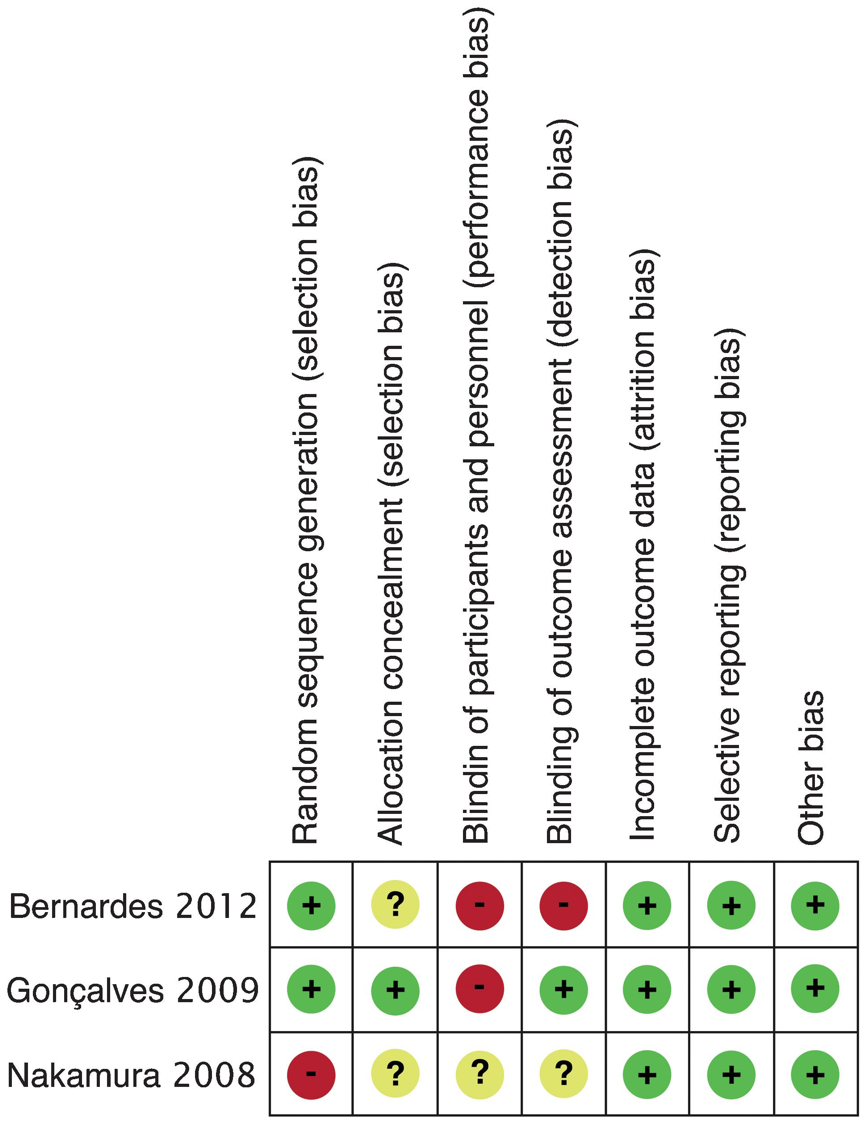 | 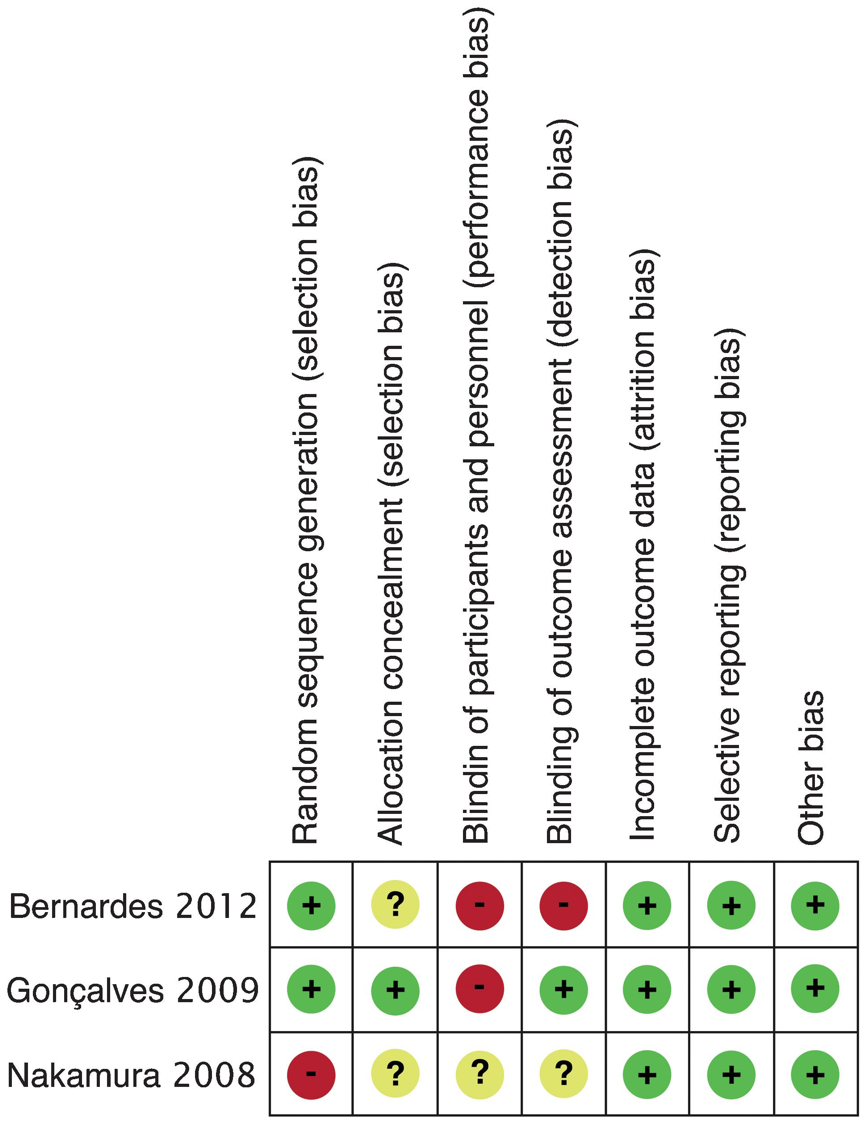 | 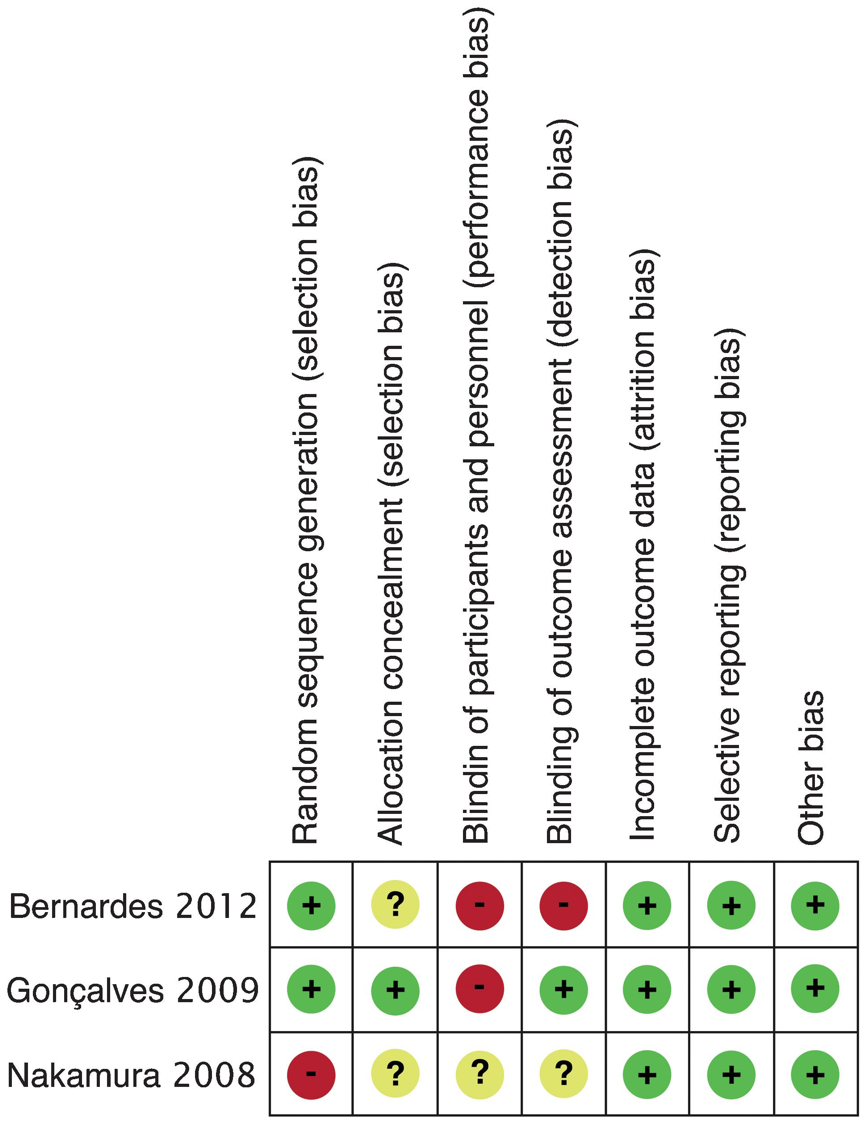 | 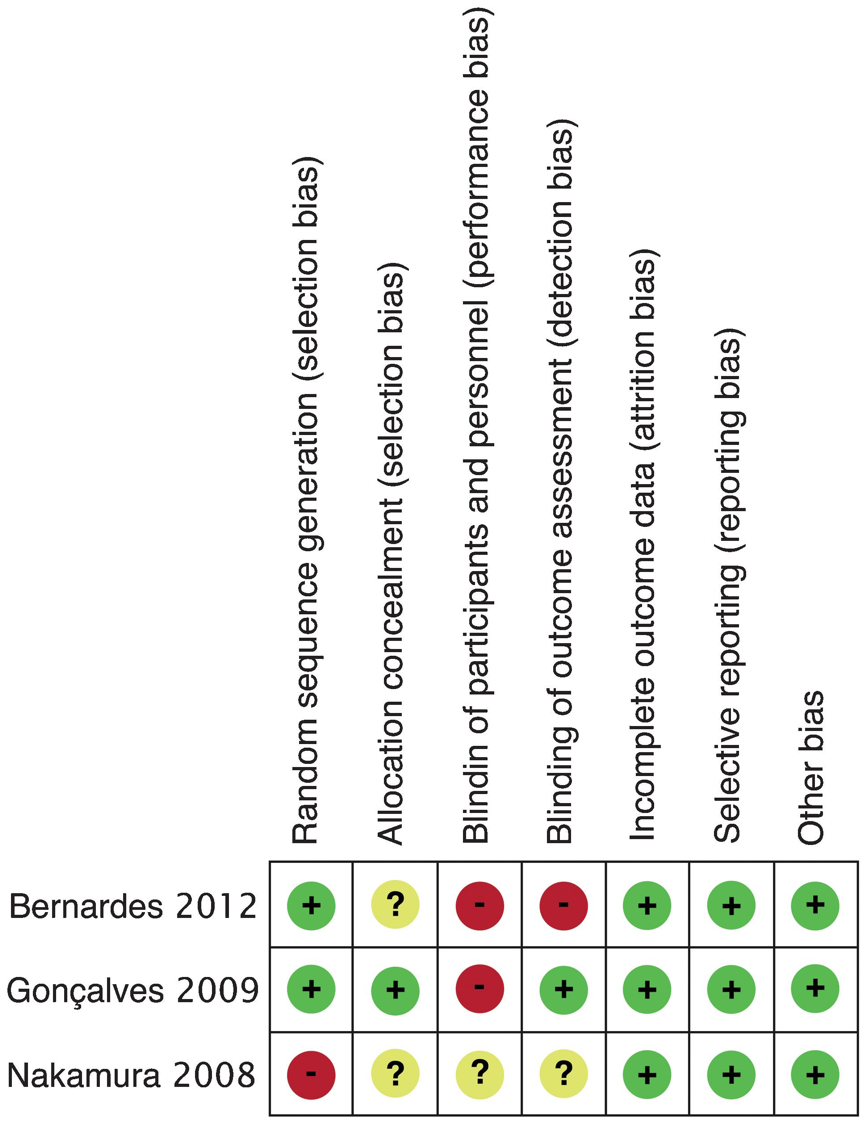 |
| Skouroliakou et al. (2018) ^(34)^ | 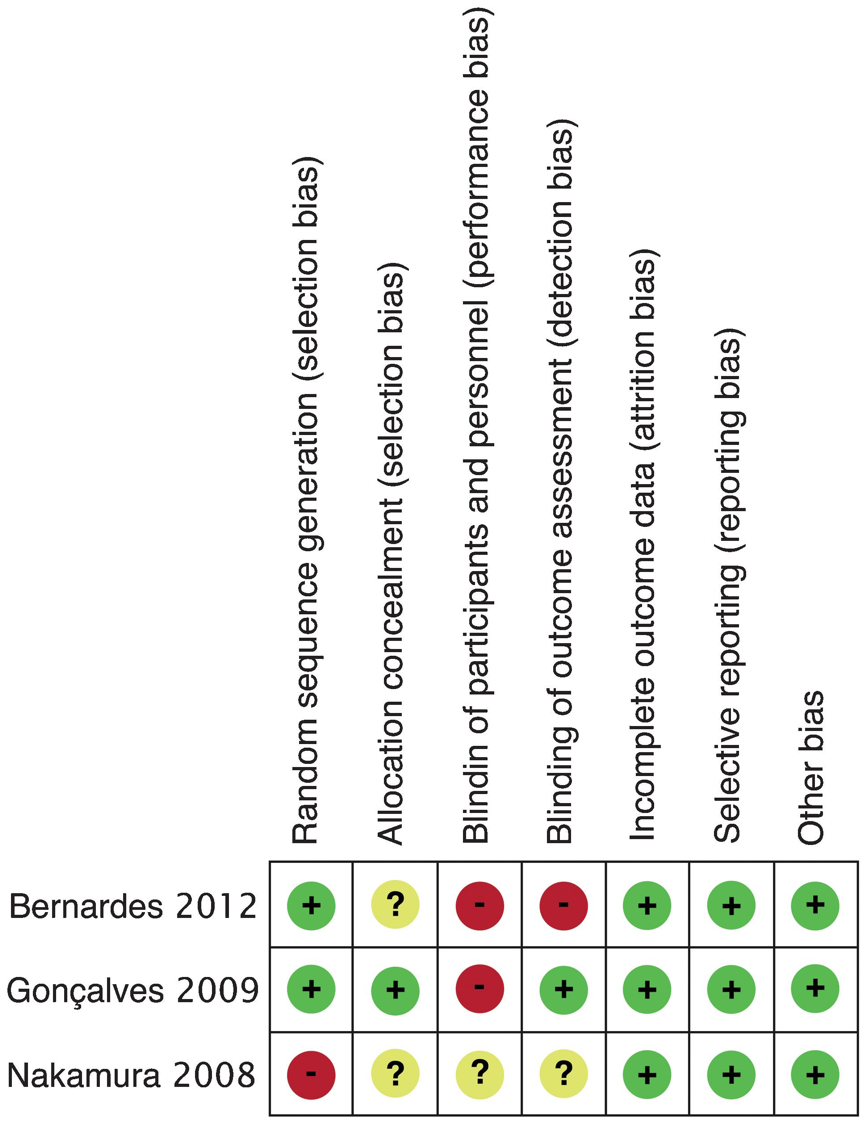 | 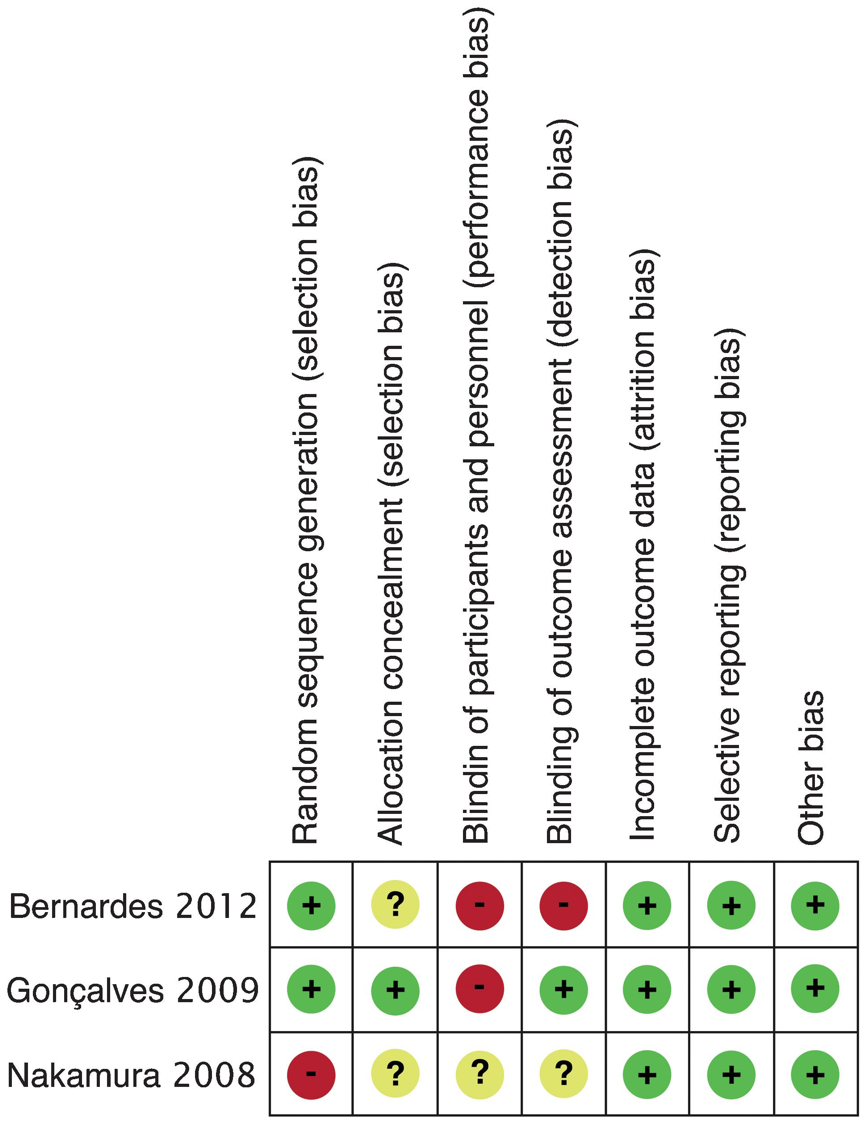 | 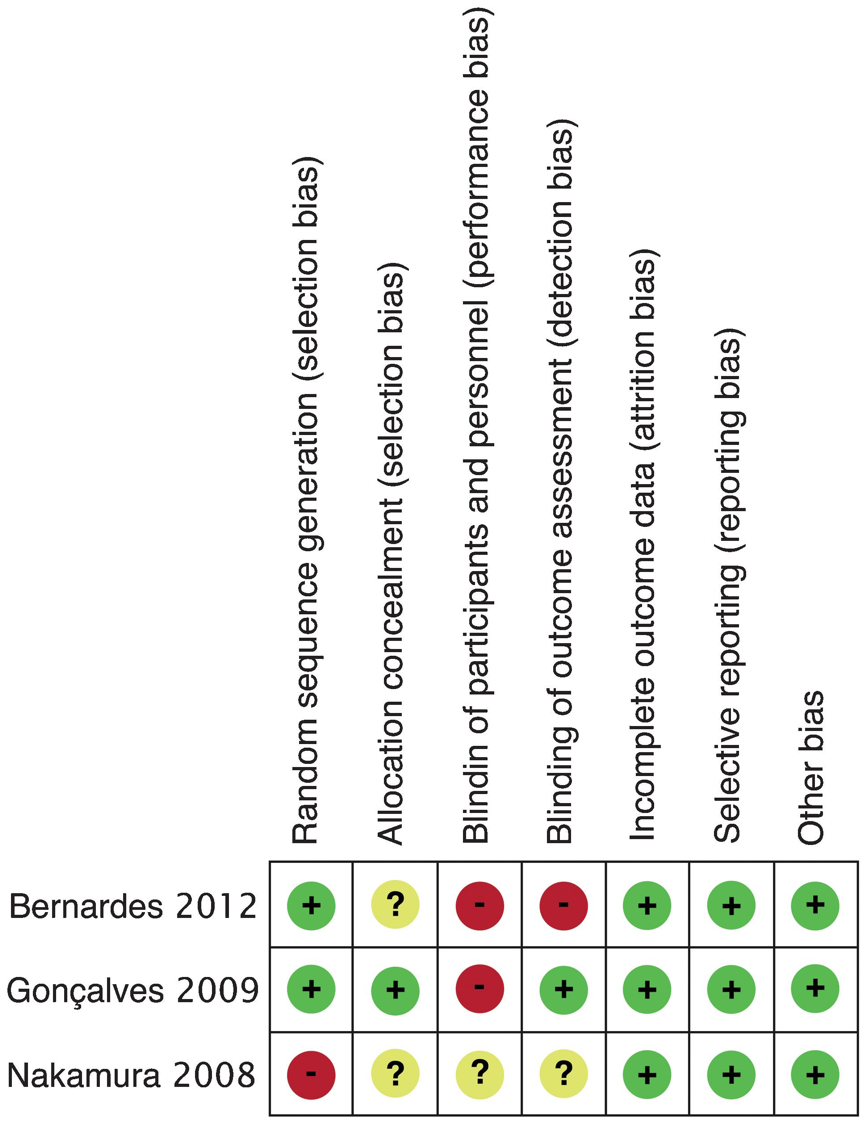 | 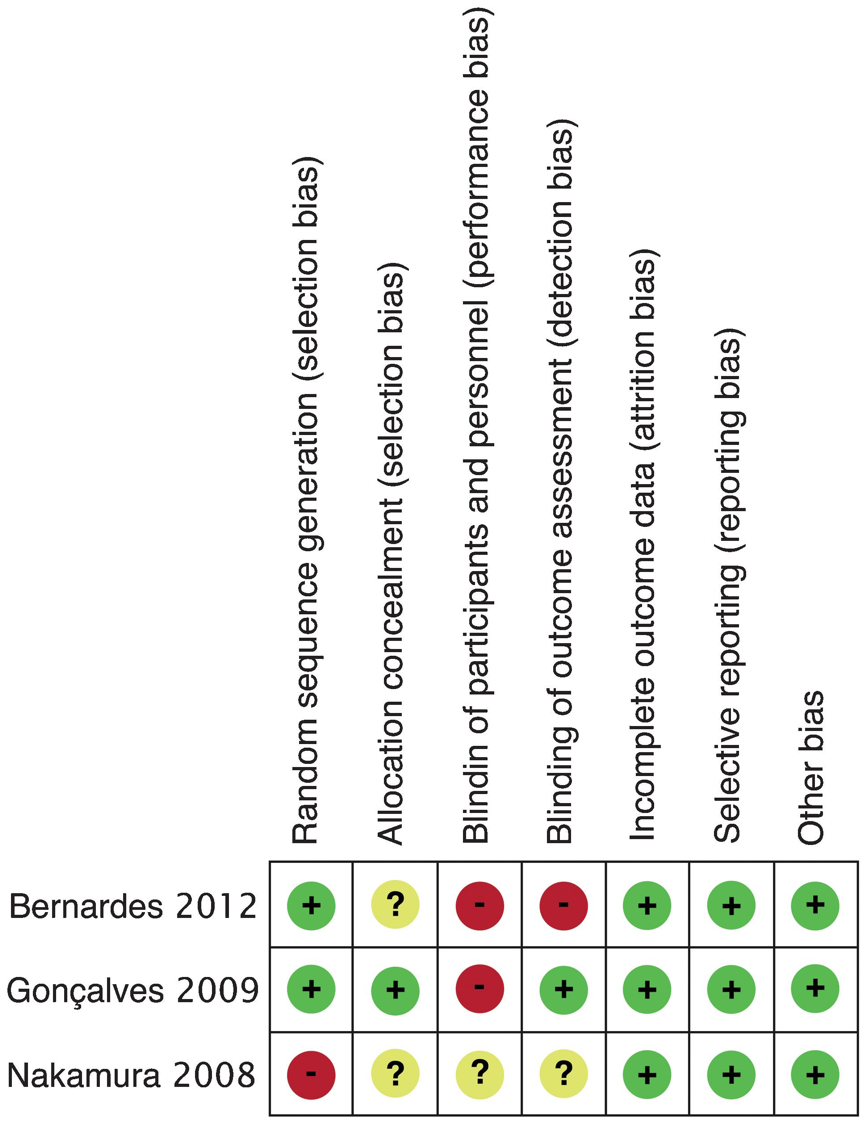 | 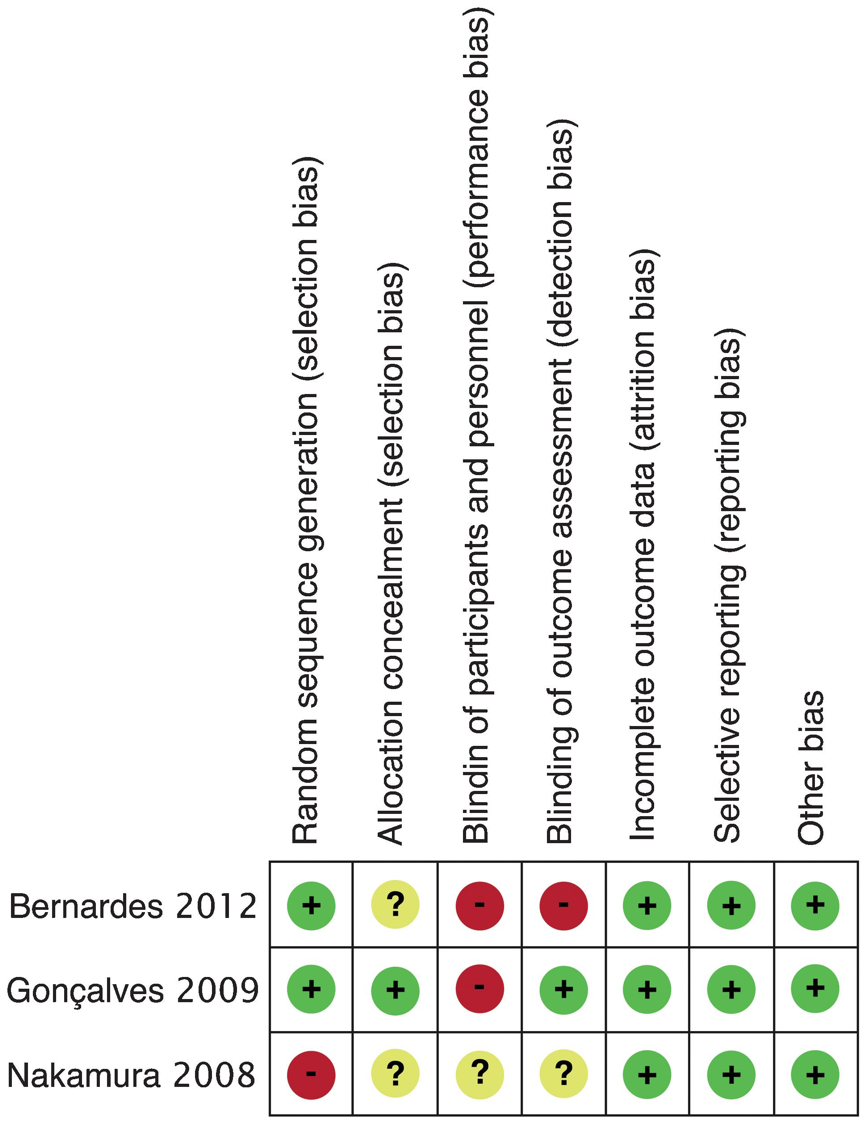 | 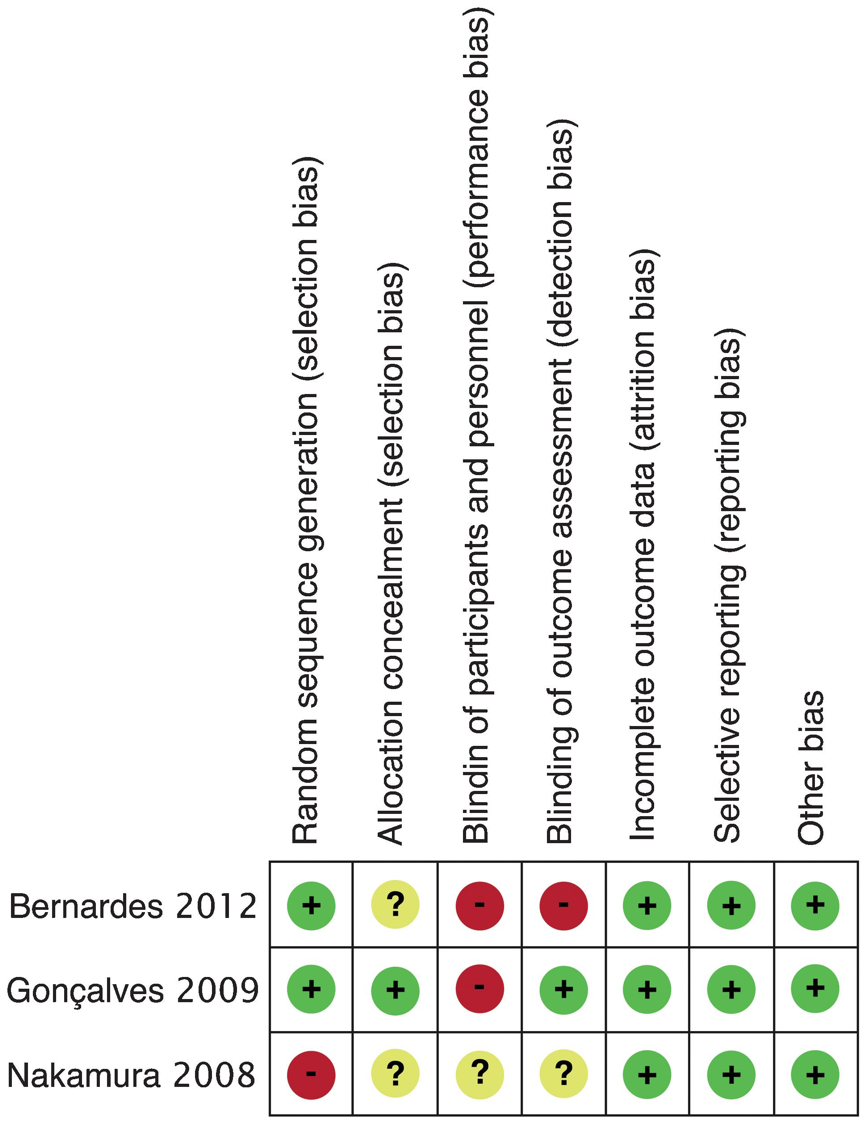 | 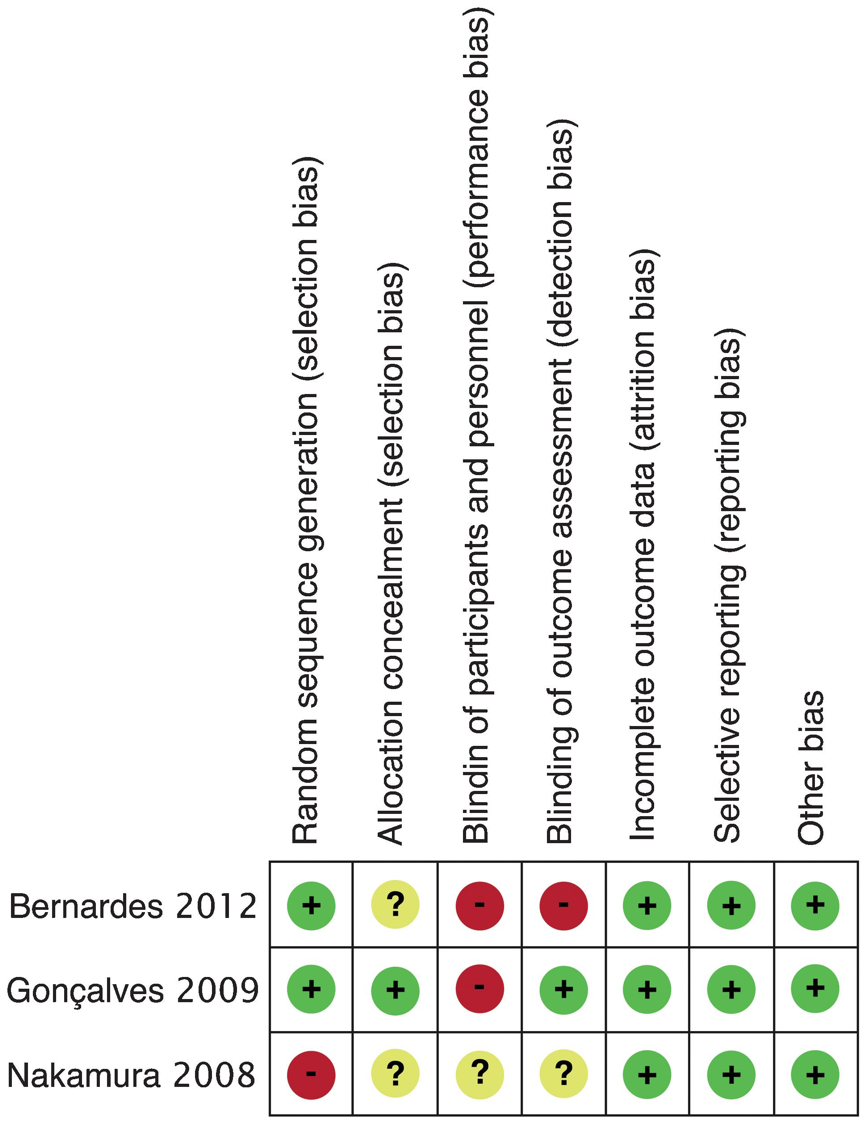 |
| Villarini et al. (2012) ^(35)^ | 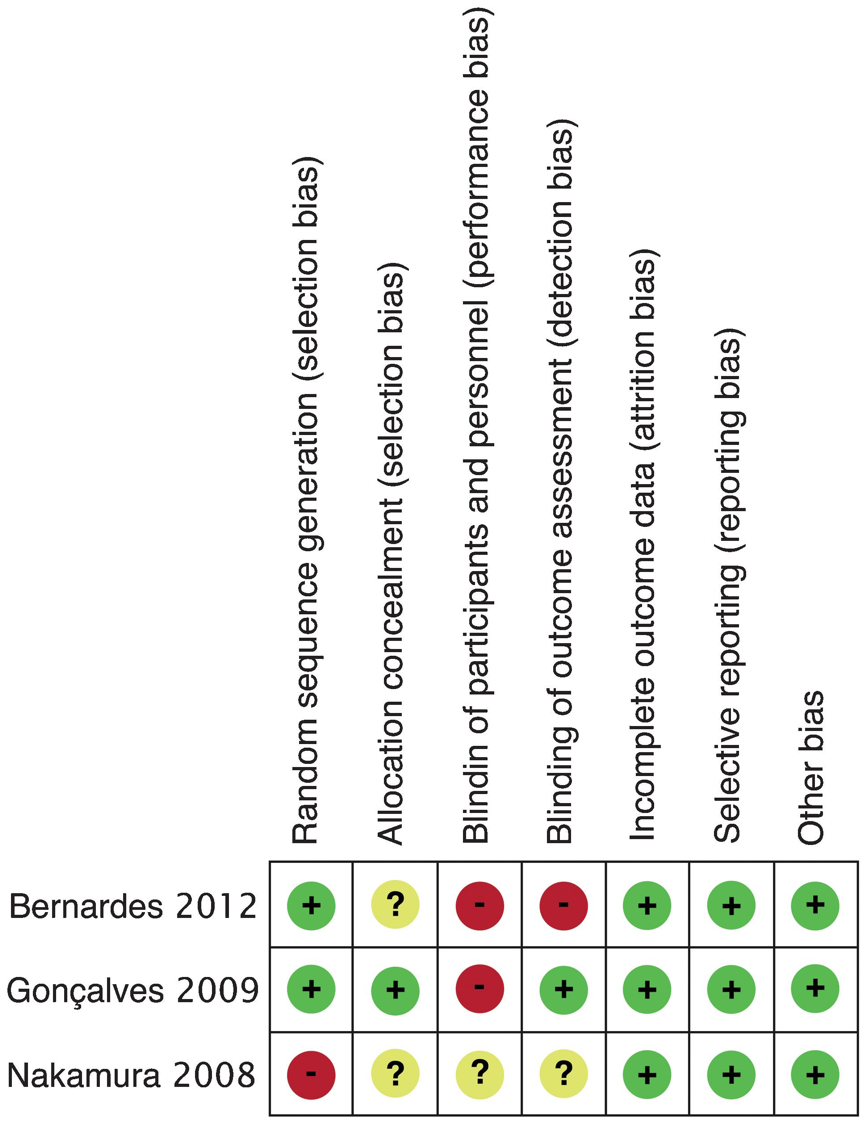 | 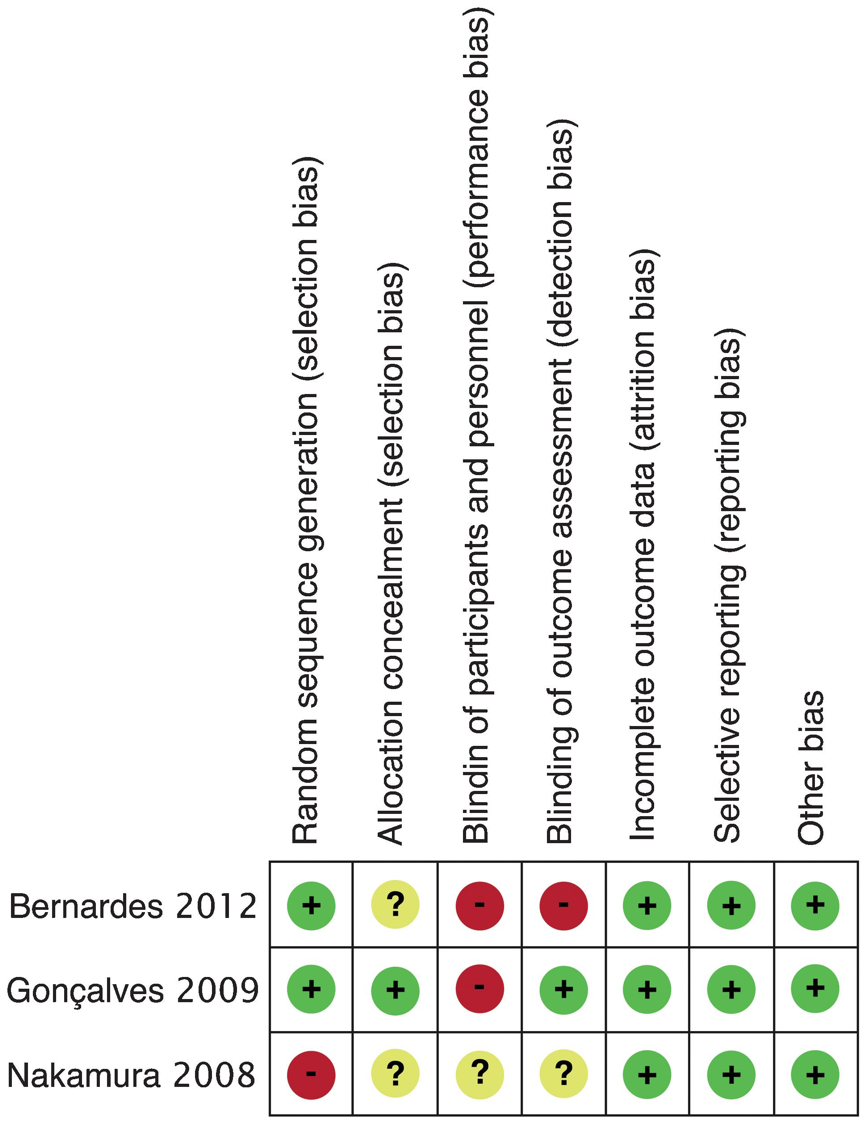 | 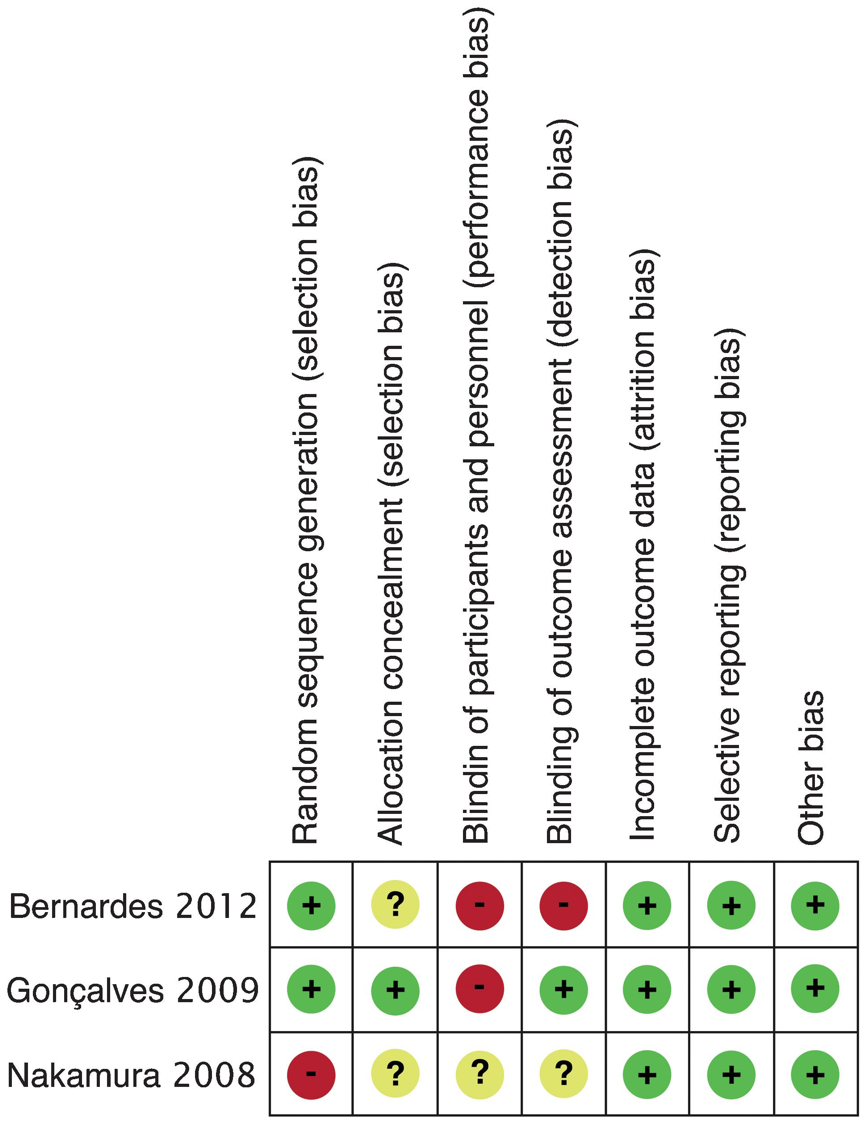 | 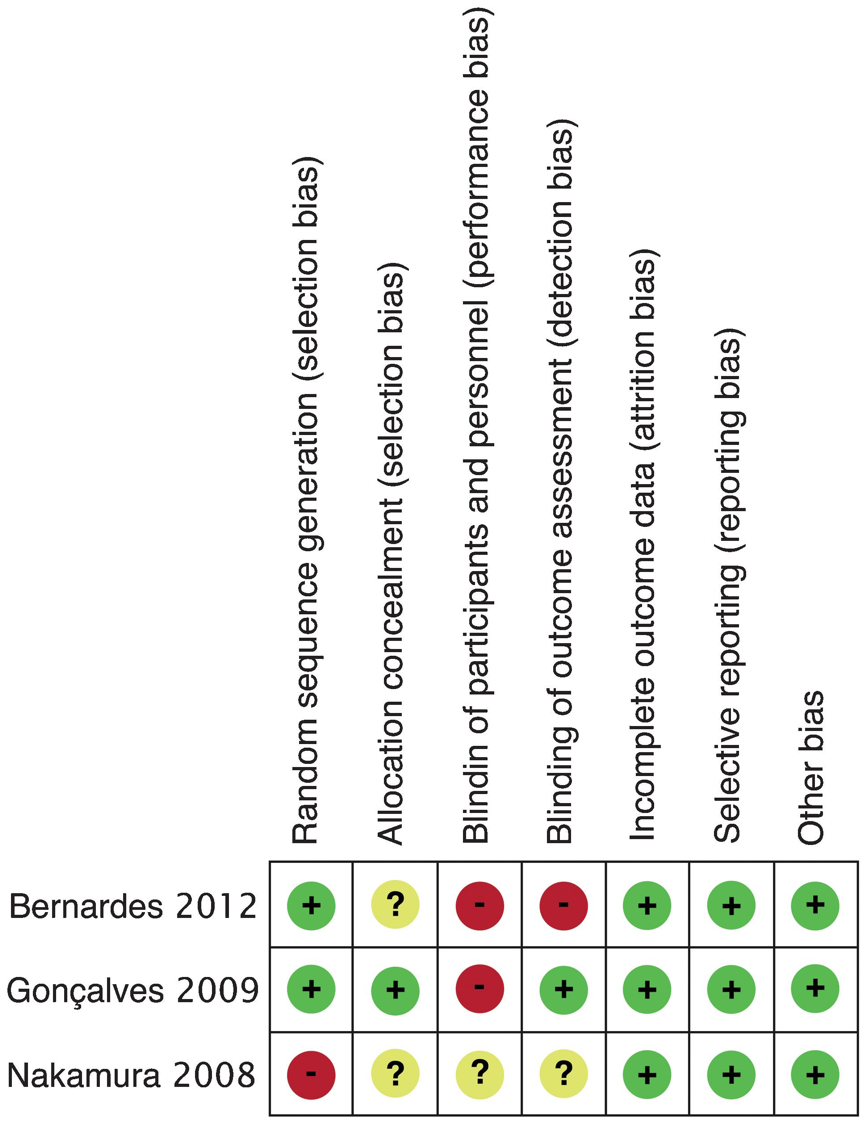 | 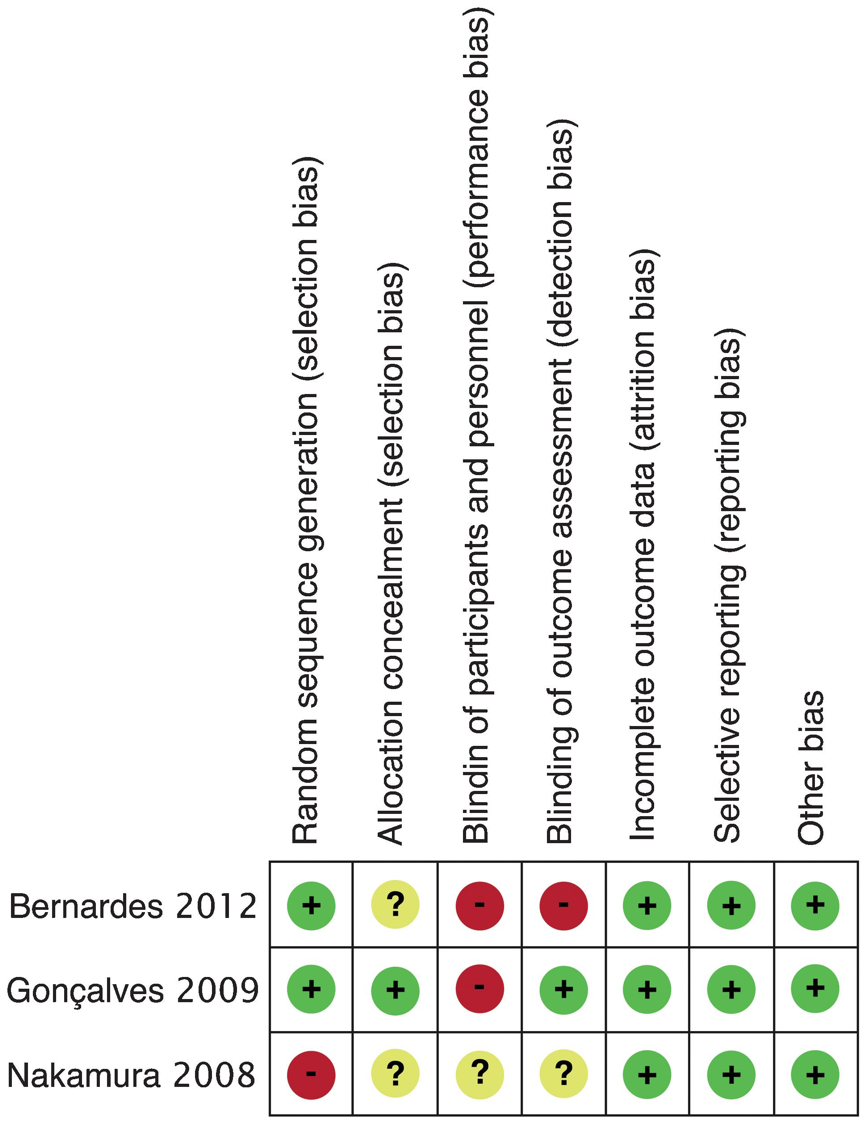 | 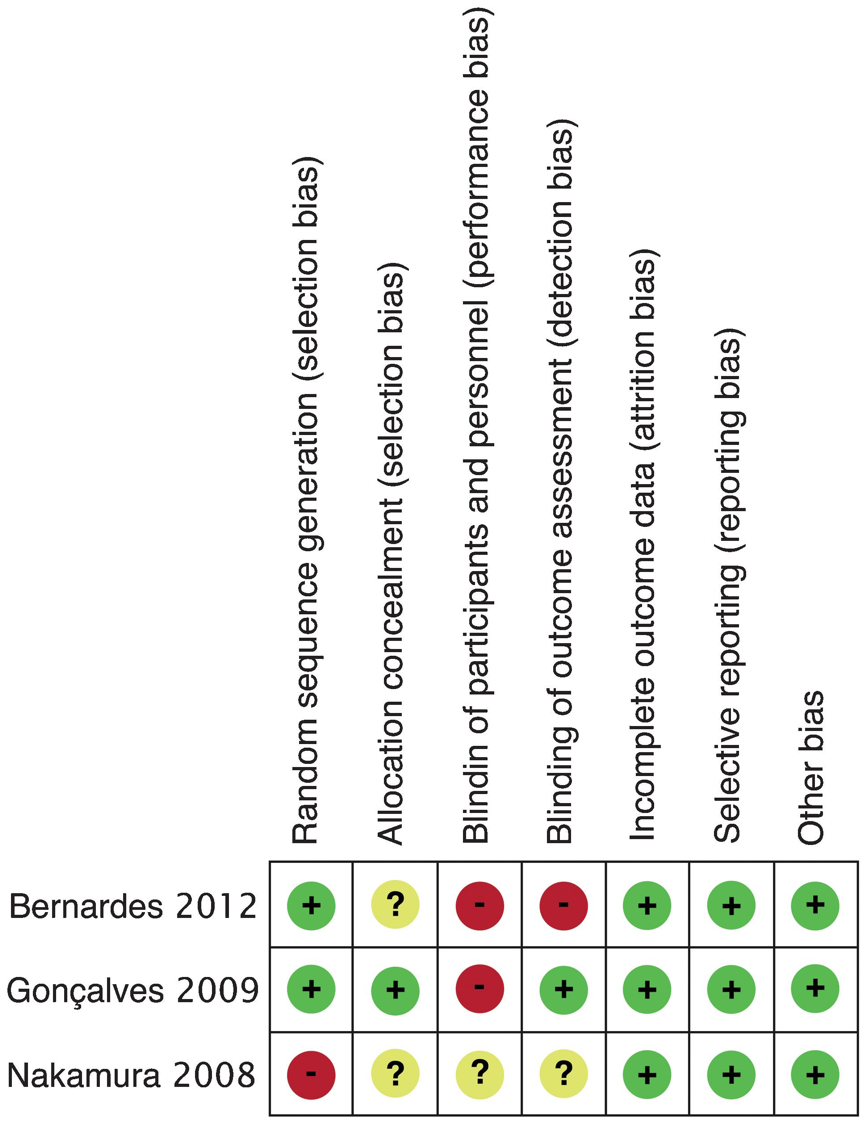 | 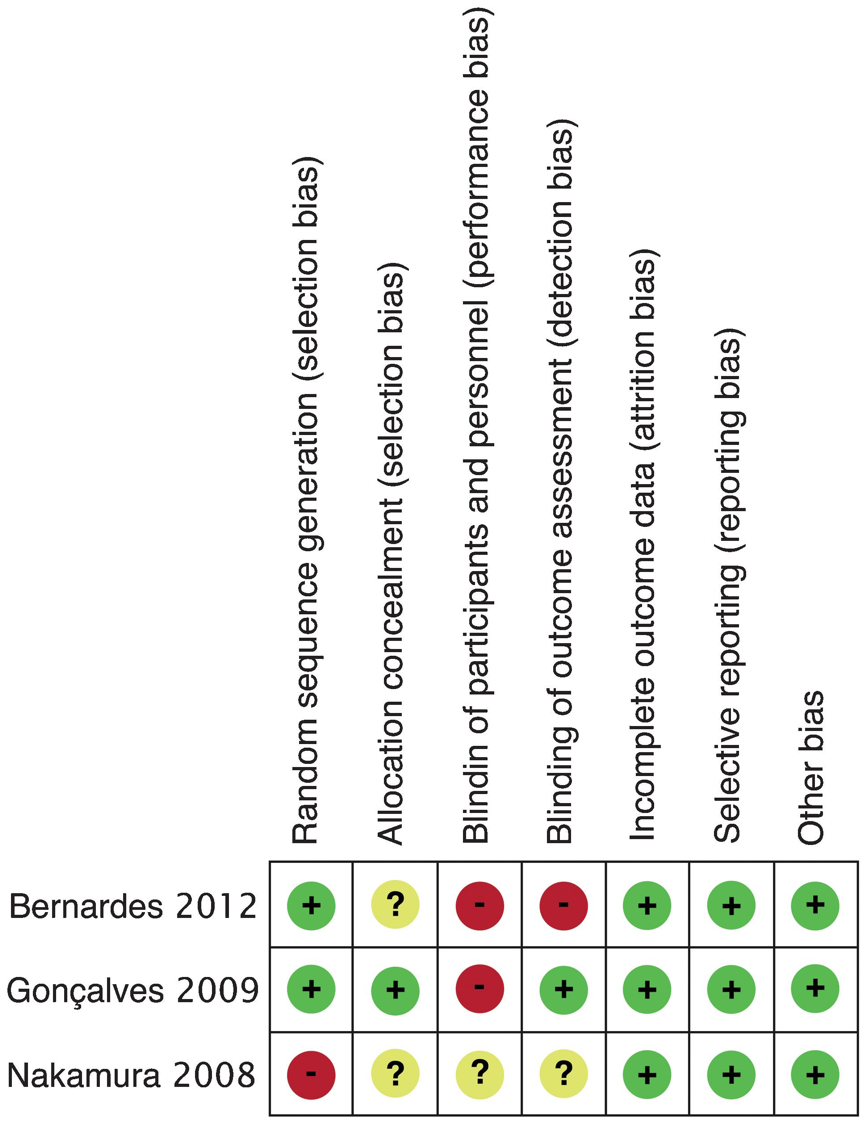 |
| Zungia et al. (2019) ^(36)^ | 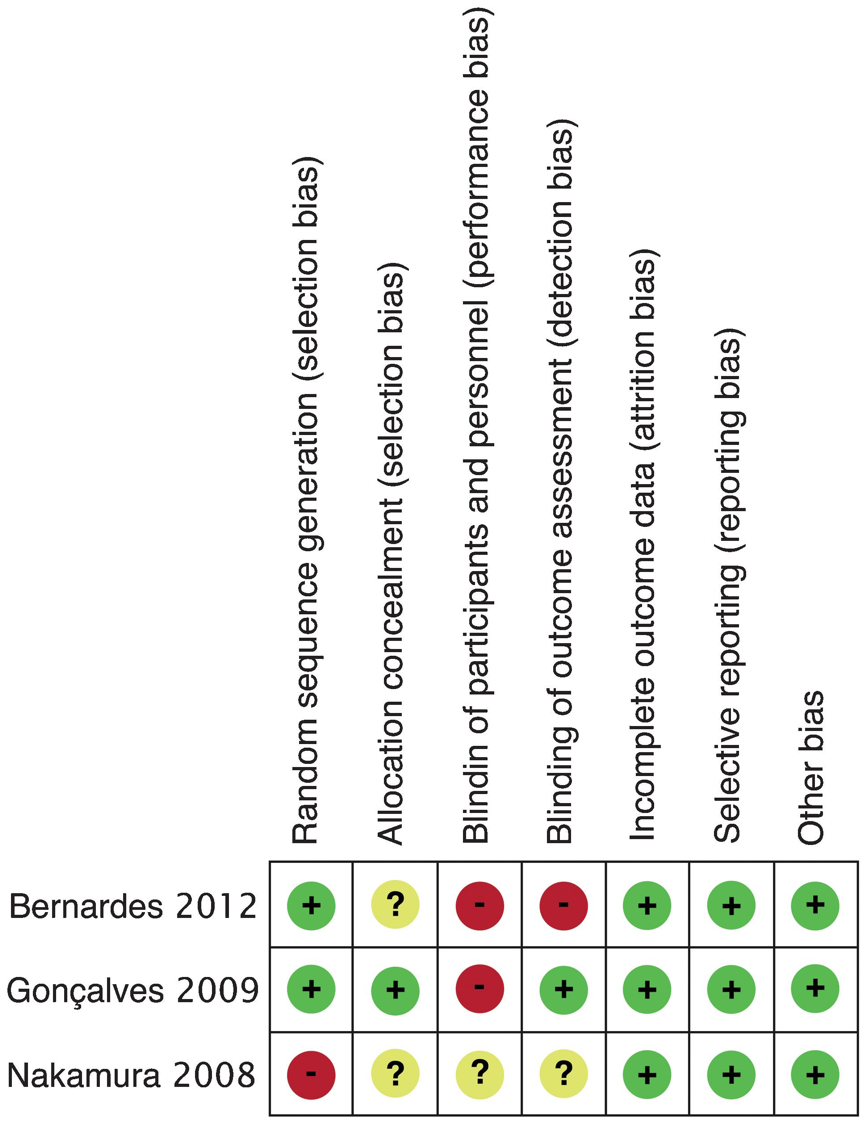 | 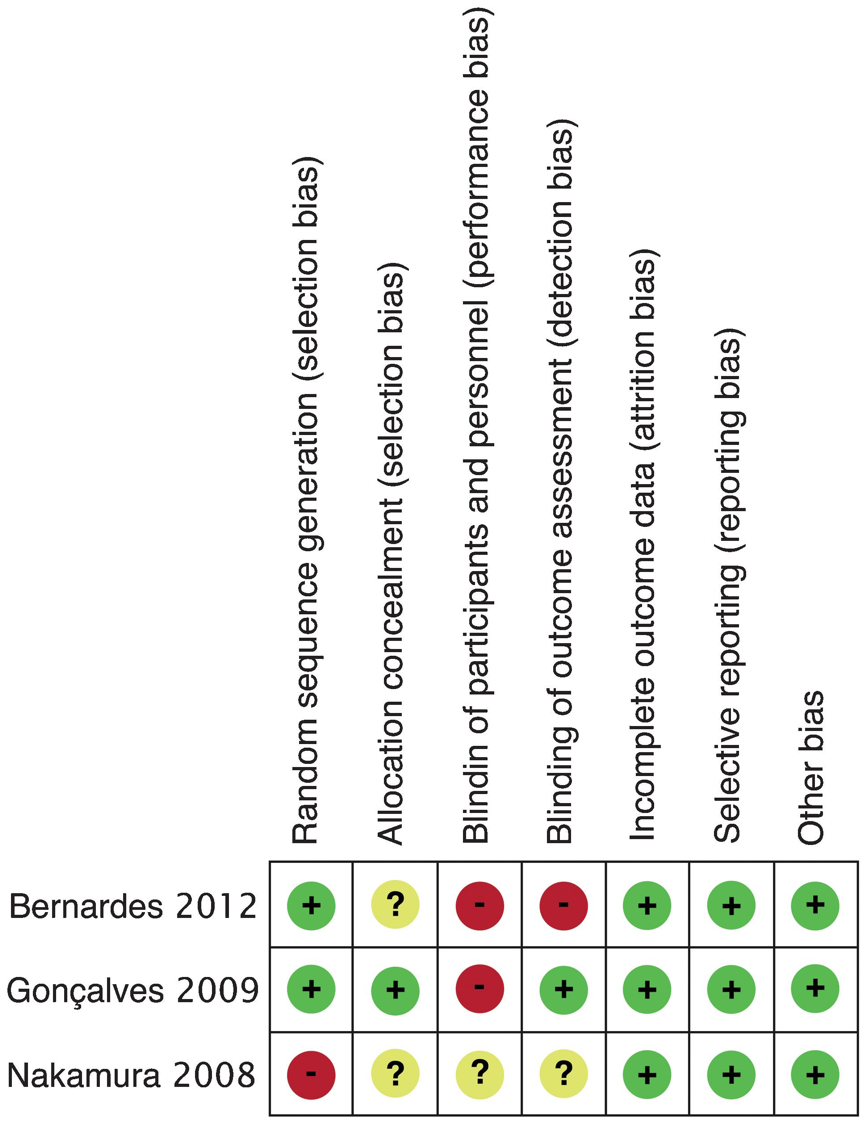 | 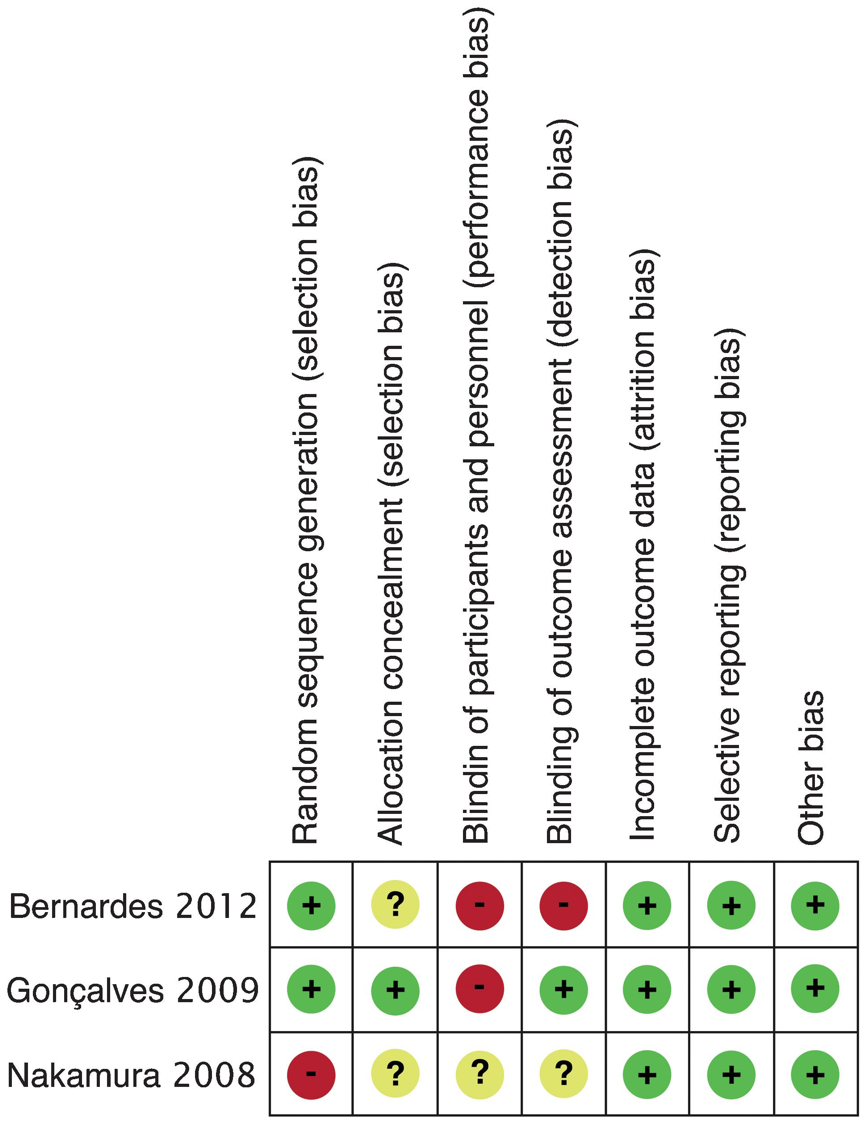 | 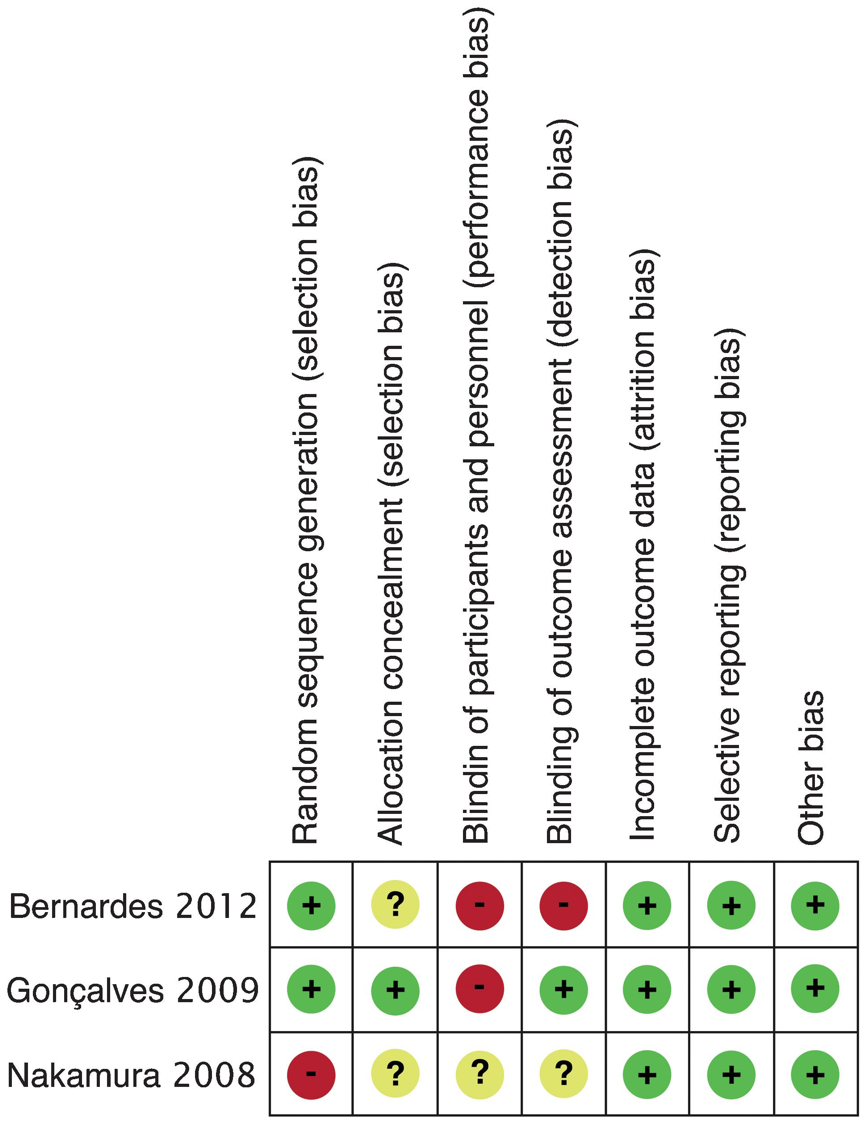 | 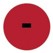 | 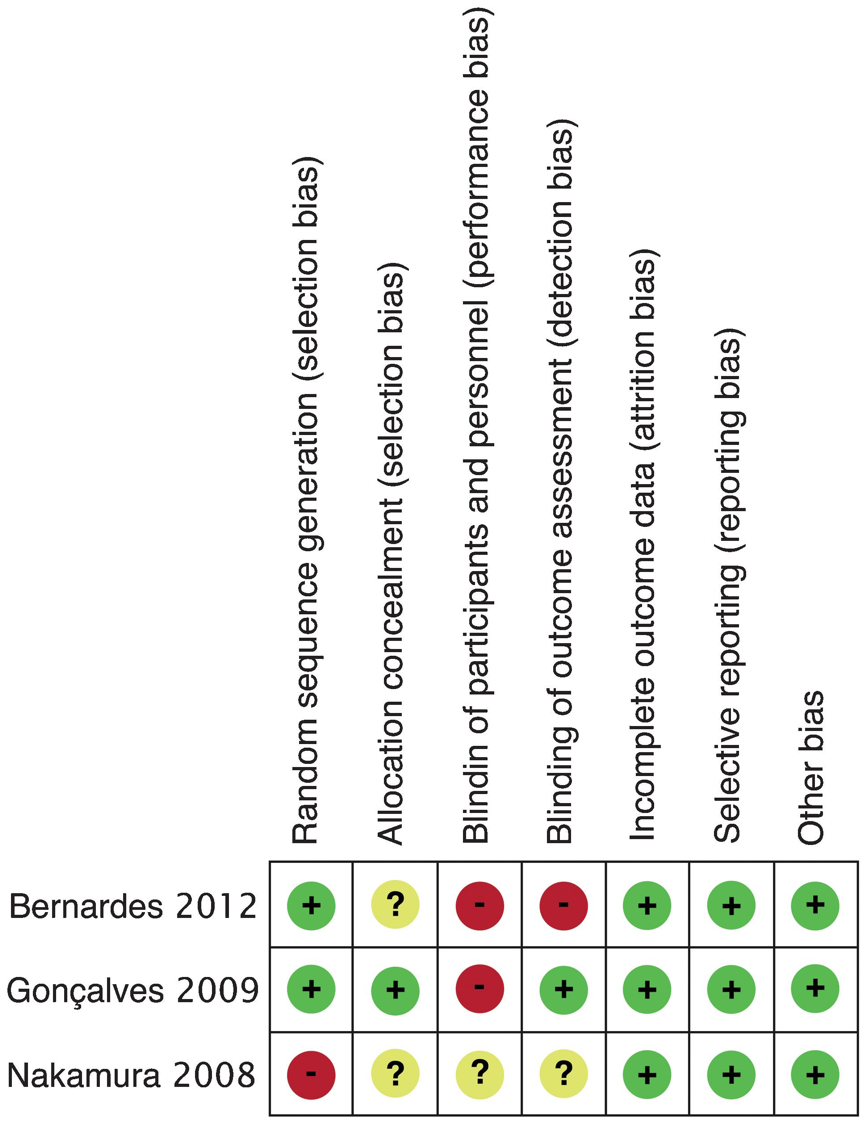 | 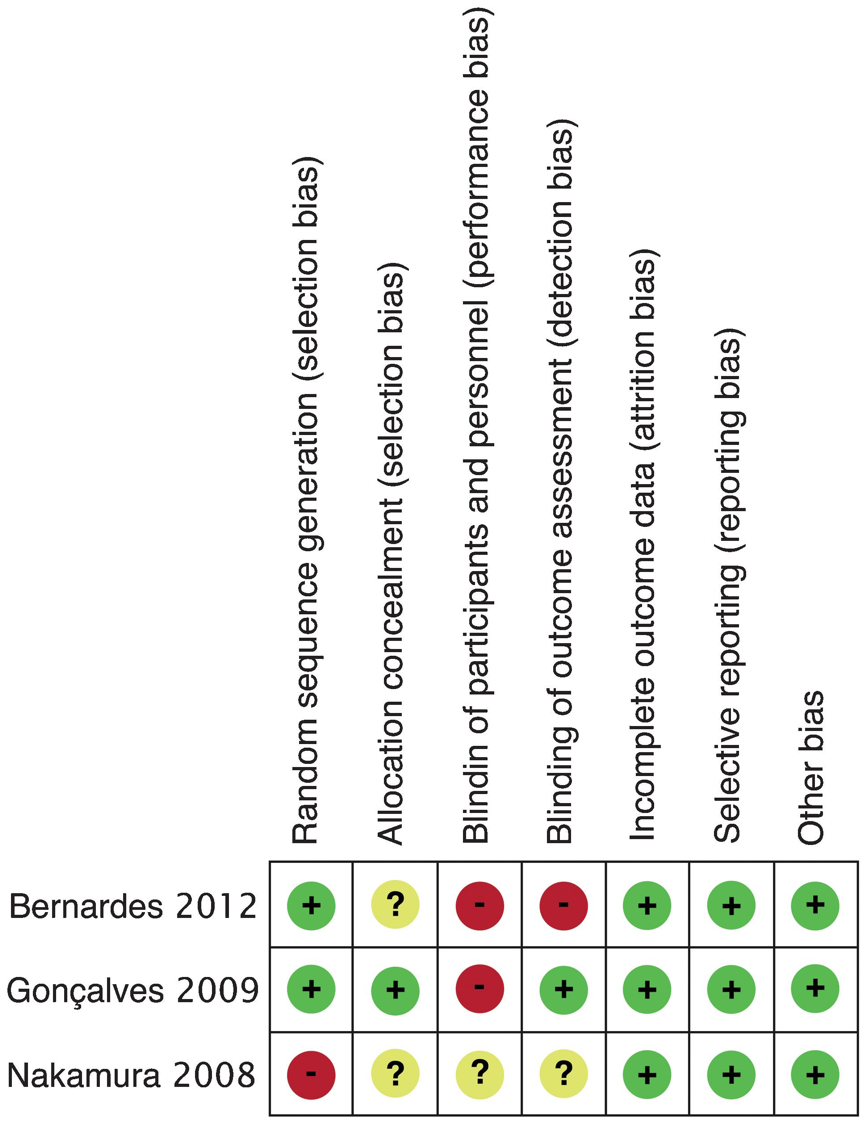 |
|  |  |  |  |  |  |  |  |

**McHugh et al., Mediterranean-style dietary interventions in adults with cancer: A systematic review of the methodological approaches, feasibility, and preliminary efficacy**

**Suppl. Material 2:** Cochrane Risk of Bias Assessment

‘+’ low risk of bias, ‘-’ high risk of bias, ‘?’ unclear risk of bias
